# Temperature-Dependent Nonlinear Calibration of Glass pH Electrodes for Negative pH Applications

**DOI:** 10.1021/acsomega.6c01903

**Published:** 2026-07-02

**Authors:** Sarawud Saleesongsom, Dominik Weiss, Yves Plancherel

**Affiliations:** Department of Earth Science and Engineering, 4615 Imperial College London, Royal School of Mines, Prince Consort Road, London SW7 2AZ, United Kingdom

## Abstract

Glass pH electrodes are widely used to estimate proton activity (*a*_H^+^_) in aqueous solutions. However, at high proton activity, conventional linear calibration using standard NIST/DIN buffers breaks down due to nonlinear electrode response and strong nonideality, leading to severe pH overestimation. To alleviate this limitation, this study develops and validates a nonlinear, temperature-corrected calibration protocol that extends the measurable and reliable range of pH using glass electrodes down to (pH = −log *a*_H^+^_) = −5 (proton activity of 10^5^) over a temperature range between 5 and 60 °C. The calibration is done using NIST buffers (pH 1.68 to 10.01) and sulfuric acid standards (concentrations ranging between 0.16 mmol·L^–1^ and 9.69 mol·L^–1^ H_2_SO_4_). Proton activities are calculated using the Pitzer model with the MacInnes assumption implemented in PHREEQC. At a given temperature, the response curve between pH and the electrode electromotive force in the low to negative pH range is modeled best with a logistic equation. Incorporation of temperature-dependent parameters enables construction of continuous nonlinear calibration curves across the studied temperature range. Application of this calibration protocol reduces systematic pH overestimation by up to >3 pH units and yields accurate proton activity under strongly acidic conditions. Sensitivity analysis demonstrates that meaningful measurements are achievable down to approximately pH ≈ −5, below which signal-to-noise constraints dominate. This work establishes a transferable and practical methodology that extends the operational range of conventional glass electrodes by several orders of magnitude in proton activity, enabling more reliable pH measurements in extreme natural and industrial environments.

## Graphical abstract

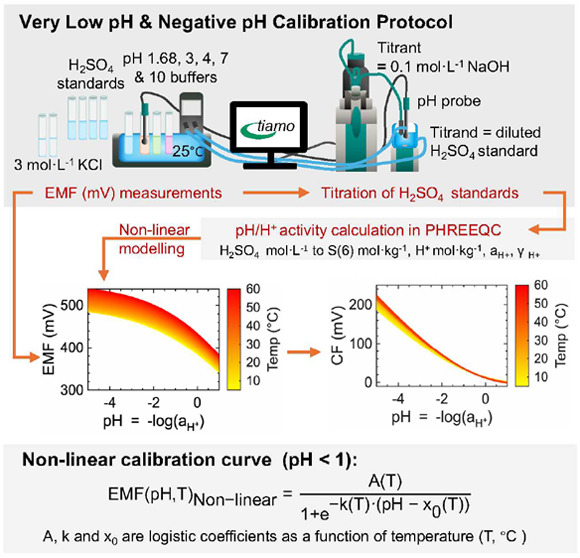

## 1 Introduction

Very low and negative pH conditions are encountered in a wide range of natural and industrial systems, including acid mine drainage, volcanic environments, atmospheric aerosols, and concentrated industrial acids.^1–10^ However, accurate determination of such pH values (defined as −log­(*a*_H^+^_), where *a*_H^+^_ is the proton activity) using glass pH electrodes remains challenging, as these glass electrodes severely underestimate proton activity at high proton concentrations.^1,2^ The main cause of this limitation is rooted in the fact that the electromotive force response of glass electrodes becomes increasingly nonlinear as conditions become more acidic, requiring a novel type of calibration protocol that can model that nonlinear behavior.^1^

Nordstrom et al. introduced a nonlinear pH calibration protocol suitable for glass electrodes and applied it to measure very acidic waters (pH = −3.6) in mining environments.^1^ While their method included measurements at different temperatures, it relied on empirical calibration rather than a generalized model describing electrode response across different pH and temperature conditions. In practice, temperature-dependent calibration is often performed by measuring electrode response in solutions at different temperatures; however, such calibrations are not transferable and cannot be reliably applied outside the specific temperature at which they were obtained. The ability to perform accurate low pH measurements across a temperature range is particularly relevant to natural and industrial systems, but development of accurate temperature-dependent calibration is difficult because both electrode response and ion activity coefficients are inherently temperature-dependent.^11,12^ At present, no general, transferable calibration model exists for accurately determining very low pH across a range of temperatures with glass electrodes, limiting the use of standard pH glass electrodes in extremely acidic environments.

Glass pH electrodes are widely recognized for their reliable performance over the pH range from 1 to 13.^11^ However, in highly acidic solutions, the electrode response deviates from ideal Nernstian behavior due to strong nonideality in proton activity and saturation effects in the glass membrane at high proton concentrations, leading to systematic measurement inaccuracies.^1,13–15^ Some commercial pH meters have built-in calibration algorithms that extrapolate the linear calibration curve beyond standard buffer points (e.g., pH 1.68, 2.00, or 4.00), with advertised operating ranges down to pH = −2 (see Supporting Information Table S2). However, these approaches assume that the electrode response remains ideal (linear, Nernstian) even in highly acidic solutions, neglecting acid error and causing the measured pH to be systematically higher than actual values.^16^

The underlying mechanism of acid error remains poorly understood, but three key factors (Figure 1) are thought to contribute most to the overall nonlinear electrode electromotive force (EMF), each arising from distinct locations within the electrode system.

**Figure 1:**
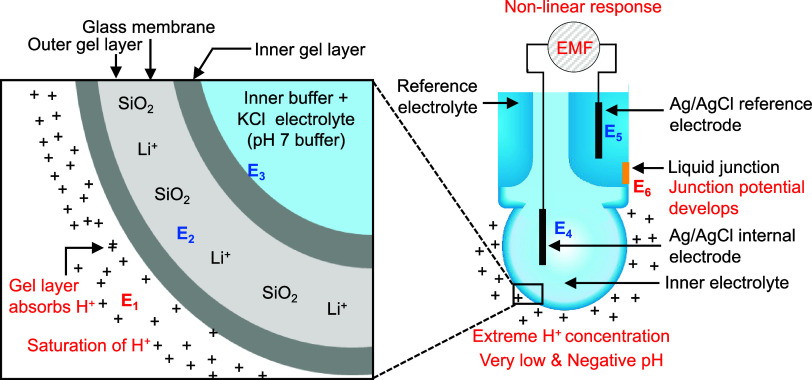
Schematic illustration of different sources leading to acid error in a combined glass pH electrode. The left panel shows the glass membrane structure with outer and inner gel layers, where proton absorption and saturation occur and affect electrode potential E_1_. The right panel shows the electrode assembly with the Ag/AgCl reference system with 3 mol·L^–1^ KCl electrolyte, highlighting the junction potential (E_6_) that developed at the liquid junction contributes to lowering the total electrode potential (EMF = E_1_ + E_2_ + E_3_ + E_4_ + E_5_ + E_6_).

1. Glass membrane surface effects (E_1_): These occur at the outer hydrated layer of the glass electrode in contact with the sample solution. At very high proton concentrations, (i) saturation of surface sites limits further proton exchange,^12^ and partial adsorption of protons into the outer membrane boundary reduces the effective proton activity at the membrane boundary.^17^ Both effects lead to deviations from ideal Nernstian behavior, lowering the measured potential.^12,17^

2. Liquid junction potential, LJP (E_6_): This LJP occurs due to the differences in ion mobilities at the interface between the reference electrolyte and the strong acid solution generate a junction potential.^16^ For solutions with ionic strength between 0.1 and 0.5 mol·L^–1^ at 25 °C, this LJP contribution is approximately 0.03 ± 0.02 pH units.^18^

In this study, acid error is treated as an overall effect manifested as an observed potential drift from the ideal Nernstian response. Rather than correcting for individual sources of error, the cumulative impact of the three contributing factors on the electrode potential is considered a single, overall acid error.

To date, two calibration methods have been developed to address the nonlinear electrode response of glass electrodes under highly acidic conditions. Nordstrom et al. applied multiple linear regression to capture electrode responses, calibrated down to pH −4 in H_2_SO_4_–FeSO_4_ solutions under temperature conditions that mimic environments of the Richmond Mine water (30 to 47 °C).^1^ Nordstrom et al. demonstrated that electrode response varies with temperature and that a separate calibration curve is required for each temperature.^1^ However, the temperature dependence of the regression coefficients was not evaluated, and the analysis was limited to the presentation of calibration curves without reporting the corresponding regression equations.

A second approach introduces a junction potential correction term directly into the Nernst equation to account for nonlinear electrode response (eq 1).^19^

1 $$\mathrm{EMF}=E_{0}+S\cdot \log\left(C_{H^{+}}\right)+J_{p}\cdot C_{H^{+}}$$

where *E*_0_ is the reference electrode potential, *S* is the Nernstian slope (temperature-dependent), and the last term represents a junction potential correction calculated as the product of a constant junction coefficient (*J*_p_, mV·L·mol^–1^) and the proton concentration (*C*_H^+^_, mol·L^–1^). Note that this correction term relies on knowing the concentration of protons, as *C*_H^+^_ is explicitly used in eq 1. The activity coefficient of proton (γ_H^+^_) is approximately unity for dilute solutions.^20^ In highly acidic solutions γ_H^+^_deviates from unity. Calculations using the Pitzer model in PHREEQC indicate that γ_H^+^_values can be as high as 1000 at 10 mol·kg^–1^ H^+^. The error introduced by assuming γ_H^+^_= 1 can be understood from the definition of pH, where pH = −log­(γ_H^+^_·*C*_H^+^_). Neglecting variations in γ_H^+^_ leads to pH deviation of magnitude ΔpH = −log­(γ_H^+^_), corresponding to an error of +3 pH units when γ_H^+^_ = 1000 at 10 mol·kg^–1^ H^+^. Therefore, activity-based calibration methods, where the electrode response is related to proton activity (*a*_H^+^_ = γ_H^+^_·*C*_H^+^_) rather than proton concentration alone, are required under highly acidic conditions to account for variations in γ_H^+^_.

The aim of this study is to develop a temperature-dependent, nonlinear mathematical model that enables accurate determination of proton activity over a wide proton concentration over the temperature range from 5 to 60 °C. The derived model extends the applicability of glass pH electrodes beyond standard limits and builds on the work of Nordstrom et al. (2000) by providing a temperature-dependent calibration across *a*_H^+^_ = 0.1 to 1·10^5^ and over the temperature range 5–60 °C. This study is structured as follows. The methodology outlines the calibration protocol and provides the analytical details of the experimental procedures and measurements. The study then introduces the negative pH calibration curve and associated correction factor at 25 °C, followed by incorporation of temperature effects on electrode response. This is extended to a comparison of nonlinear calibration behavior across different glass electrodes and an evaluation of their performance limits under extreme acidity. The breakdown of linear calibration and the resulting acid error in the negative pH range are subsequently examined, alongside a thermodynamic interpretation of the observed nonlinearity. Finally, the applicability of the developed approach is demonstrated using real-world acidic systems with synthetic acid mine drainage.

## 2 Methodology

### 2.1 Preparation of Negative pH Calibration Standards Using H_2_SO_4_ Solutions Down to pH −7.4

A series of H_2_SO_4_ calibration standards were prepared by diluting concentrated ≥95% sulfuric acid (≈17.8 mol·L^–1^ H_2_SO_4_ VWR) with Milli-Q water to achieve final concentrations ranging from 1.0·10^–4^ to 9.0 mol·L^–1^. Preparation volumes for each concentration are provided in Supporting Information S3 Table S3.

The exact concentration of each H_2_SO_4_ standard solution was determined by potentiometric titration against 0.1 mol·L^–1^ NaOH using the Tiamo 3.0 software (Metrohm AG).^21^ Prior to titration, the glass vessel, magnetic stirrer, dosing unit tube, and pH electrode were rinsed thoroughly with deionized water. Samples above 0.1 mol·L^–1^ H_2_SO_4_ were prediluted with Milli-Q water to minimize acid error and improve pH measurement accuracy, since potentiometric titration relies on reliable pH electrode response that follows Nernstian behavior. The actual concentration was then back-calculated from the applied dilution factor. A combined pH electrode with an Ag/AgCl reference and 3.0 mol·L^–1^ KCl electrolyte (Metrohm Primatrode I or Unitrode N) was used and fully submerged by adding at least 15 mL of combined Milli-Q water and H_2_SO_4_ solutions to the vessel. The exact Milli-Q water and H_2_SO_4_ standard volumes used in each titration are summarized in Supporting Information S4 Table S4.

### 2.2 Proton Activity Calculations Using the Pitzer Model and the MacInnes Convention

The theoretical pH values used in calibration were estimated from proton activities calculated by the PHREEQC Version 3 program^22^ using the Pitzer database, which incorporates the Pitzer aqueous model^23^ and the MacInnes assumption for scaling individual ion activity coefficients.^24^ The Pitzer model allows calculation of activity coefficients for ions in concentrated electrolyte solutions initially developed for high salinity waters. In PHREEQC, each model run was set with a neutral solution (pH 7.00) and maintained charge balance at a fixed temperature. Each molarity concentration of H_2_SO_4_ (in mol·L^–1^) was determined experimentally by potentiometric titration and was used as input for calculating proton activities in PHREEQC. Density values corresponding to each H_2_SO_4_ concentration, ranging from 1.0 g·cm^–3^ at 1.6·10^–4^ mol·L^–1^ to 1.7 g·cm^–3^ at 7.68 mol·L^–1^, were applied to convert units between molarity and molality. PHREEQC internally adjusts solution density and performs charge balance based on the input of H_2_SO_4_ concentration, subsequently computing proton activity, pH, chemical speciation, molality-based concentrations, MacInnes activities, and MacInnes logarithm of activities,^22^ see Supporting Information Section S5 Table S8 for detail. The uncertainty in pH values estimated from the ± SD of the experimentally determined H_2_SO_4_ concentrations ranges from 0.00 to 0.09 pH units, with an average error of 0.01 ± 0.02 pH units.

For H_2_SO_4_ solutions approaching the upper working limit of the Pitzer model of 8.50 mol·L^–1^ H_2_SO_4_, PHREEQC is unable to reliably compute density and convert the unit from molarity to molality at such high concentration of H_2_SO_4_. In such cases, the concentration was manually converted from molarity (mol·L^–1^) to molality (mol·kg^–1^) prior to input into PHREEQC using tabulated density data.^25^

### 2.3 EMF Measurement Procedure and Construction of a Calibration Curve for Negative pH at 25 °C

A Metrohm combined glass pH electrode H (Primatrode, 6.0228.020), equipped with an integrated NTC temperature sensor and an Ag/AgCl reference electrode in 3 mol·L^–1^ KCl electrolyte, was used to measure the EMF response of Hanna commercial pH buffers and H_2_SO_4_ calibration standards. The electrode was connected to a Metrohm 867 pH module and operated through the Tiamo software.

At the beginning of each experimental session, a standard 3-point pH calibration was carried out at 25 °C in pH calibration mode using the Tiamo software and buffer solutions of pH 4.01, 7.01, and 10.01. Electrode potentials were automatically corrected for temperature effects. Calibration parameters, including the standard potential (*E*_0_) and slope (*S*), were determined based on the linear Nernst eq (eq 2). Electrode performance was verified and deemed acceptable when the slope was in the range from 95% to 102% of the theoretical Nernstian value at 25 °C, which is 59.1 mV·pH^–1^, and the zero-point pH (pH_0_), defined as the pH at which the working electrode potential equals that of the reference electrode potential (i.e., zero net glass electrode response), fell within the range pH 6.75 and 7.25.

2 $${\mathrm{EMF}\left(\mathrm{pH}\right)}_{\mathrm{Nernst}}=E_{0}-S\cdot \mathrm{pH}$$

Following standard calibration, EMF responses were measured in Hanna pH buffer solutions (pH 3.00 and 1.68) and subsequently in the H_2_SO_4_ standard solutions. For each buffer and H_2_SO_4_ solution, 2 mL was dispensed into a 15 mL test tube and equilibrated in a water bath at 25 °C. Measurements were carried out in order of increasing concentration using the potentiometric voltage (donated as U) measurement mode in the Tiamo software. The maximum reading time was set to 1000 s to ensure that electrode potentials reached a stable value. In practice, stable readings were typically obtained within 300 s. A stability criterion was applied whereby readings were considered stable when potential drift was below 0.5 mV·min^–1^; EMF readings were logged every 2 s. After each measurement, the electrode was rinsed with deionized water for 10 s, blotted dry with a soft tissue, and conditioned in 2 mL of 3 mol·L^–1^ KCl. The KCl solution was replaced whenever its pH changed by >0.5 units to avoid cross-contamination and to allow the hydrated gel layer of the glass membrane to re-equilibrate. Measurements were repeated over multiple days to avoid prolonged exposure of the electrode to strong acids, allow the electrode to recover between runs, and assess reproducibility. All H_2_SO_4_ standards were measured in triplicate.

A negative pH calibration curve was constructed by fitting a logistic function (eq 3) to the EMF-pH data obtained from buffer solutions (pH 1.68, 3.00, and 4.01) and sulfuric acid (H_2_SO_4_) standard solutions. The fitted parameters are defined as follows: *A* (mV) is the amplitude parameter, representing the total EMF change; *k* (pH^–1^) is the slope factor, describing the steepness of the response; and *x*_0_ (pH units) is the inflection point, corresponding to the pH at which the EMF reaches half of its maximum value.

3 $${\mathrm{EMF}\left(\mathrm{pH}\right)}_{\mathrm{Non}-\mathrm{linear}}=\frac{A}{1+e^{-k\cdot \left(\mathrm{pH}-x_{0}\right)}}$$

To evaluate the transferability of the calibration, the entire calibration protocol was independently repeated using three additional combined glass electrodes (Metrohm Unitrode N, Orion Ross Sure-Flow, and Orion Ross Ultra pH/ATC Triode), each equipped with an Ag/AgCl reference electrode in 3 mol·L^–1^ KCl electrolyte. All measurements were conducted under identical conditions, enabling direct comparison of calibration responses across different electrode types

### 2.4 Derivation and Determination of a Temperature-Dependent Correction Factor (CF) for Negative pH Calibration

The negative pH calibration curve constructed using the logistic function defined in eq 3 and the extrapolated linear Nernstian calibration were used to derive a correction factor (CF) that varies with pH. CF is defined as the difference between ideal (linear) and observed nonlinear (logistic) EMF values (eq 4)­

4 $${\mathrm{CF}\left(\mathrm{pH}\right)=\mathrm{EMF}\left(\mathrm{pH}\right)}_{\mathrm{Nernst}}-{\mathrm{EMF}\left(\mathrm{pH}\right)}_{\mathrm{Non}-\mathrm{linear}}$$

where CF is expressed in mV. A CF value of zero indicates ideal Nernstian behavior, while nonzero values quantify the deviation of the measured EMF from the ideal Nernstian response. The CF therefore provides a quantitative measure of nonlinearity (acid error) as a function of pH. The effect of temperature on the correction factor is investigated by repeating the experiments across the temperature range from 5 to 60 °C. The temperature-dependent, practical form of the Nernst equation is described by eq 5.^26^

5 $${\mathrm{EMF}\left(T\right)}_{\mathrm{Nernst}}=E_{0\;\mathrm{at}\;25{}^{\circ}C}+\frac{{dE}_{0}}{dT}\cdot \left(T-25\right)-K_{s}\cdot (T+273.15)\cdot \mathrm{pH}$$

where

$E_{0\;\mathrm{at}\;25{}^{\circ}C}$ = standard potential at 25 °C,

$\frac{dE_{0}}{dT}$ = a rate of change of the standard potential as a function of temperature,

*K*_s_ = temperature coefficient of Nernstian slope (S).

Experimental *E*_0_ and *S* values were extracted from Nernst plots obtained at different temperatures. The value of *E*_0 at 25 °C_ and $\frac{{dE}_{0}}{dT}$ were determined by a linear regression of *E*_0_ versus temperature (*T* in °C), while *K*_s_ was obtained by fitting a linear regression of *S* versus *T*.

6 $$\mathrm{EMF}{\left(\mathrm{pH},T\right)}_{\mathrm{Non}‐\mathrm{linear}}=\frac{A\left(T\right)}{1+e^{-k\left(T\right)\cdot \left(\mathrm{pH}-X_{0\left(T\right)}\right)}}$$

In eq 6, *A*, *k*, and *x*_0_ are logistic function coefficients that vary with temperature (*T* in °C). At each calibration temperature, a logistic function was fitted to experimental EMF-pH data to determine the coefficients *A*(*T*), k­(*T*), and *x*_0_(*T*). The temperature dependence of each coefficient was then parametrized using polynomial functions (eqs 7–9); the degree of each polynomial was selected according to the AICc criterion^27^ (see Supporting Information Section S10). Here, *n* represents the order of the polynomial function required to adequately describe the temperature dependence of each parameter. The coefficients *C_i_* are the fitted polynomial parameters (with *i* = 0, 1,..., *n*) that quantify the contribution of each temperature term *T^i^* to the overall polynomial function. These temperature-dependent functions can subsequently be used in eq 6 to calculate the EMF for any pH and *T* combination within the experimental bounds of the experiments.

7 $$A{\left(T\right)}_{n}=C_{\mathrm{nA}}T^{n}+C_{\left(n-1\right)A}T^{\left(n-1\right)}+C_{\left(n-2\right)A}T^{\left(n-2\right)}+...+C_{0A}$$

8 $$k{\left(T\right)}_{n}=C_{\mathrm{nk}}T^{n}+C_{\left(n-1\right)k}T^{\left(n-1\right)}C_{\left(n-2\right)k}T^{\left(n-2\right)}+...+C_{0k}$$

9 $${x_{0}}_{\left(T\right)n}=C_{\mathrm{nx}0}T^{n}+C_{\left(n-1\right)x0}T^{\left(n-1\right)}+C_{\left(n-2\right)x0}T^{\left(n-2\right)}+...+C_{0x0}$$

Finally, the temperature-dependent expressions for EMF­(*T*,pH)_Nernst_ and EMF­(*T*,pH)_Nonlinear_ were combined in the CF equation with the addition of temperature dependence (eq 10). This formulation enables quantification of the deviation of the measured EMF from ideal Nernstian behavior as a function of both pH and temperature.

10 $${\mathrm{CF}\left(\mathrm{pH},T\right)=\mathrm{EMF}\left(\mathrm{pH},T\right)}_{\mathrm{Nernst}}-{\mathrm{EMF}\left(\mathrm{pH},T\right)}_{\mathrm{Non}-\mathrm{linear}}$$

### 2.5 Validation of the pH Calibration Model Using Synthetic Acid Mine Drainage Samples

Synthetic acid mine drainage (AMD) solutions were prepared by mixing concentrated sulfuric acid with iron­(II) chloride tetrahydrate (FeCl_2_·4H_2_O, ReagentPlus, ≥98%, Sigma-Aldrich). These solutions are used to validate negative pH measurements using the nonlinear calibration model in eq 6 in comparison to values derived from a standard linear Nernstian pH calibration.

A series of 30 mL H_2_SO_4_ solutions were prepared with concentrations ranging from 1.00 to 6.00 mol·L^–1^, and each was combined with FeCl_2_ to achieve final concentrations of FeCl_2_ 0, 0.1, 0.5, and 1.0 mol·L^–1^. Two mL aliquots of each mixture were transferred into 15 mL test tubes. EMF measurements were obtained using Metrohm Primatrode H electrode and converted to pH values using both the classical linear Nernstian calibration approach (eq 2), and the new negative pH calibration, EMF­(pH, *T*)_Nonlinear_ model in eq 6. The resulting pH measurements were compared with theoretical pH values calculated in PHREEQC, with sulfur (S­(VI)), iron (Fe), and chloride (Cl^–^) specified as solution components.

## 3 Results and Discussion

### 3.1 Negative pH Calibration Curve and the Correction Factor at 25 °C

The negative pH calibration curve for the Metrohm Primatrode electrode H at 24.0 ± 0.3 °C (mean ± SD, *n* = 24), spanning a standard buffer range from pH 10 to 1.68 and extending down to pH −7.4 using H_2_SO_4_ standards, is shown in Figure 2a (red line). The logistic fit, as described in Section 2.4, achieved an *R* ^2^ value of 1.00, with amplitude (*A*) = 517.9 ± 4.8, growth rate (*k*) = −0.4659 ± 0.0167, and midpoint (*x*_0_) = 2.734 ± 0.056 (mean ± 95% CI) (eq 11).

11 $${\mathrm{EMF}}_{\mathrm{non}‐\mathrm{linear}}=\frac{517\pm 4.8}{1+e^{\left(0.4659\pm 0.0167\right)\cdot \left(\mathrm{pH}-\left(2.734\pm 0.056\right)\right)}}$$

5000 Monte Carlo simulations were performed using the experimental uncertainties (EMF SD = 2 ± 2 mV; pH SD = 0.02 ± 0.03 pH units) to propagate measurement error on the fitting parameters. The resulting distributions of the logistic coefficients were effectively identical to those obtained from the original fit, indicating that measurement uncertainties do not influence the parameter estimates (see Supporting Information, Section S10, Table S31).

**Figure 2:**
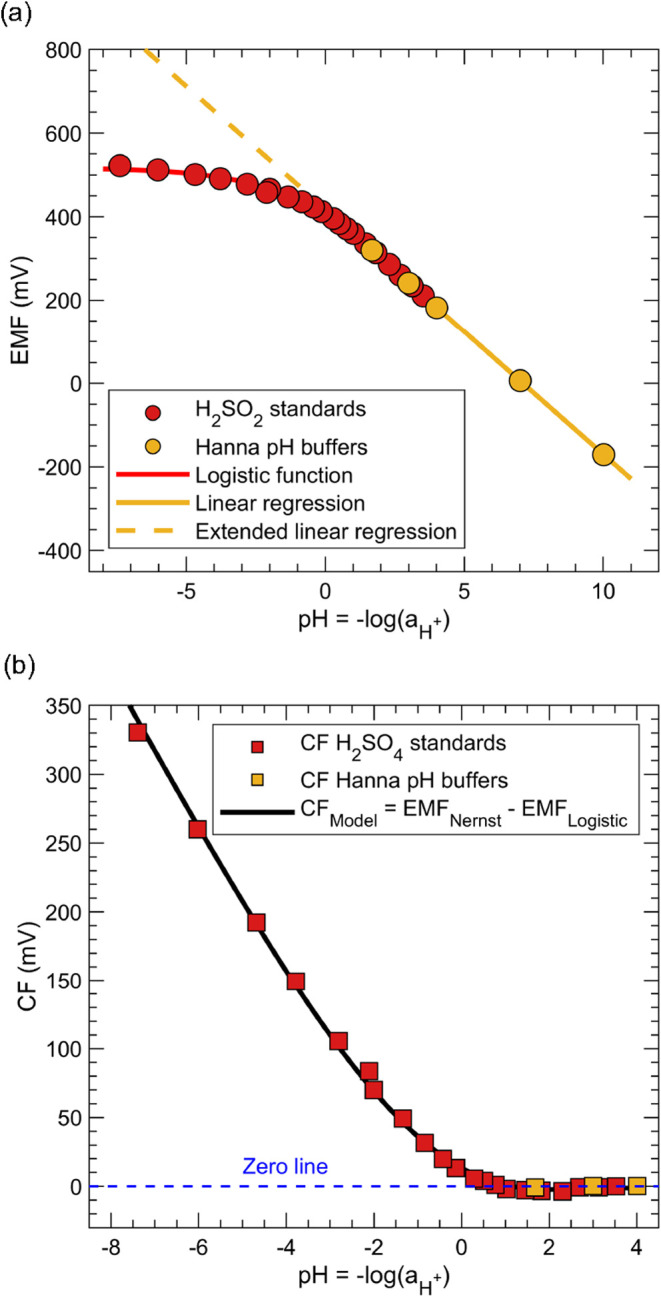
Calibrations of electrode response in the range of pH −7.4 to 10.01 and corresponding correction factor. (a) pH vs EMF plot, comparing measured EMF values from H_2_SO_4_ standards and Hanna pH buffers to the standard pH calibration (EMF_Nernst_, solid orange line) and the negative pH calibration derived from the nonlinear logistic fit (EMF_Nonlinear_, solid red line). The Nernstian response was extrapolated from the standard pH calibration between pH 4 and 10. Error bars are not shown due to small variability of their standard deviation (SD) compared to the size of the plotted data set: Each average EMF measurements had SDs of 0.2 to 2.5 mV (average 0.7 mV), and pH measurement uncertainties ranged from 0.00 to 0.09 pH units (average 0.01). (b) pH vs correction factor (CF) plot, calculated as the difference between EMF_Nernst_ and EMF_Nonlinear_.

The standard linear pH calibration curve (eq 12) at 24.4 ± 0.1 °C (mean ± SD, *n* = 3) has an intercept (*E*_0_) of 418.0 ± 11.1 mV and a slope *S* = 58.8 ± 1.5 mV pH^–1^ (mean ± 95% CI), with *R* ^2^ = 1.00 (Figure 2a) corresponding to 99.58% of the theoretical Nernstian slope at 24.4 ± 0.1 °C.

12 $${\mathrm{EMF}}_{\mathrm{Nernst}}=\left(418.0\pm 11.1\right)-\left(58.8\pm 1.5\right)\cdot \mathrm{pH}$$

Two types of CF values at 25 °C are calculated as the difference between either the measured EMF or EMF_Non–linear_ model (eq 11) and the experimental Nernstian electrode response (eq 12). Between pH 1 and 4, both modeled and measured CF values (Figure 2b) remain close to zero, with only minor offset, e.g., CF = −2.4 mV at pH 2.0, corresponding to a deviation of ∼0.04 pH unit. This minor offset indicates that, in this range, the electrode response is still linear with respect to pH. The electrode response becomes increasingly nonlinear at pH < 1, as indicated by a rapid increase in the CF. This observation is consistent with previous studies reporting the onset of acid error and initiation of non-Nernstian behavior at similarly low pH values, including pH < 0.5,^1^ pH < 1.8,^28^ and pH < 2.^17^ The CF rises to +36.2 mV at pH −1, +156.8 mV at pH −4.0 and is projected to exceed +373.4 mV at pH −8.0. Without applying this CF correction, the electrode would overestimate the pH by approximately +0.6 pH units at a true pH of −1 and by as much as +2.7 pH units at a true pH of −4.

### 3.2 Implementation of Temperature Effect on pH Glass Electrode Response and Correction Factor

The glass pH electrode response is strongly temperature dependent across both the conventional and negative pH ranges (Figure 3). The temperature dependence of the Nernstian slope and the standard potential was quantified using linear regression (illustrated in Supporting Information Figure S12), yielding the following relationships (eqs 13 and 14)­

13 $$\begin{array}{l} \begin{array}{ll} S\left(T\right)=\left(1.99\pm 0.10\cdot 10^{-1}\right)\cdot \left(T+273.15\right)+\left(-0.54\pm 3.21\right) & \left(R^{2}=1.00\right) \end{array} \end{array}$$

14 $$\begin{array}{ll} E_{0}\left(T\right)=\left(418.9\pm 1.4\right)+\left(1.13\pm 0.08\right)\cdot \left(T-25\right) & \left(R^{2}=0.99\right) \end{array}$$

where *T* is temperature (°C), *S*(*T*) is the temperature-dependent electrode slope (mV·pH^–1^), and *E*_0_(*T*) is the temperature-dependent standard potential (mV). Reported values are presented with their corresponding 95% confidence intervals.

**Figure 3:**
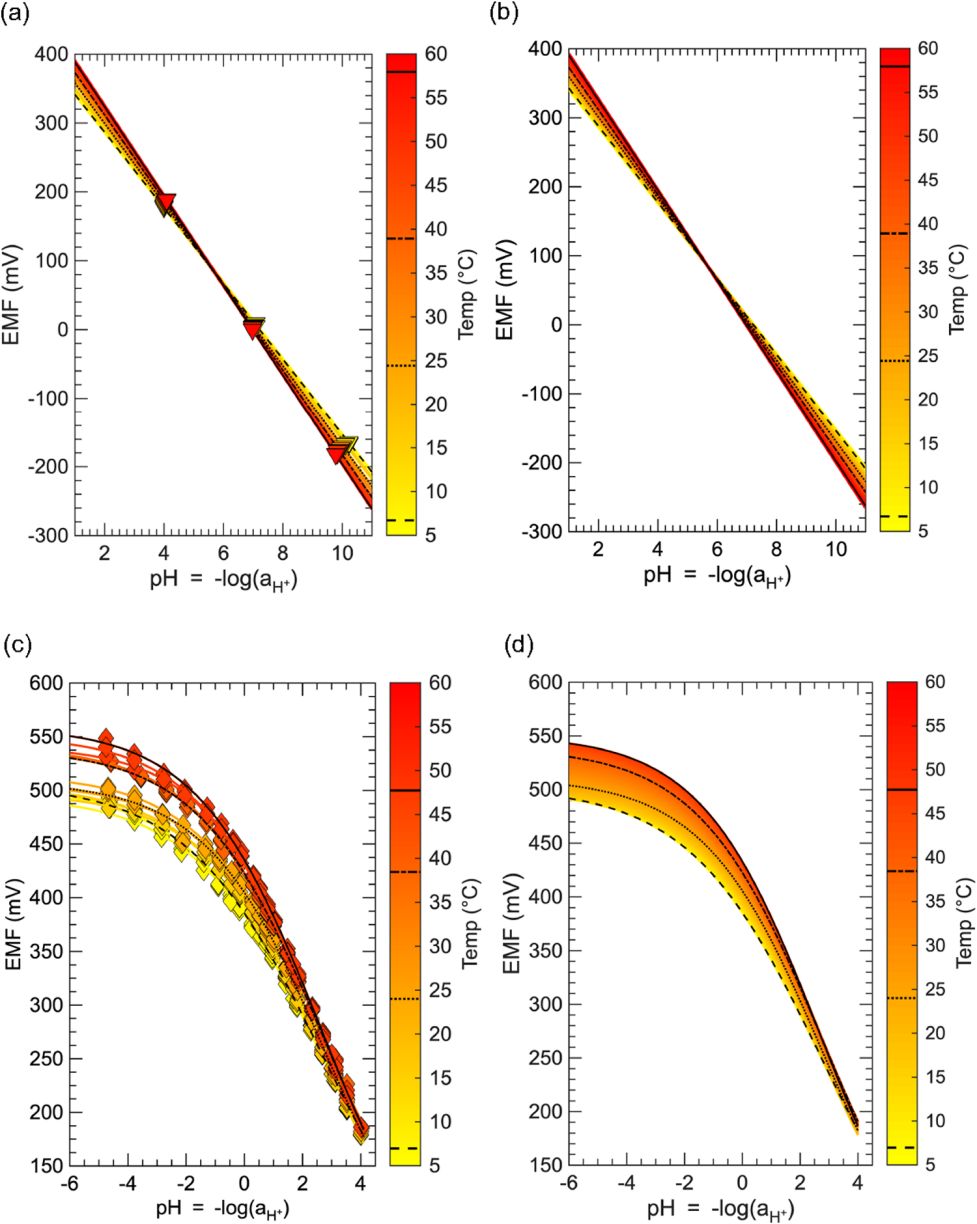
Temperature dependence of EMF-pH response of a pH glass electrode. (a) Standard pH calibrations derived from 3-point calibration at pH 4.01, 7.01 and 10.01, measured between 5 and 60 °C, fitted with linear regression. (b) Modeled temperature dependence of standard linear pH calibrations in the pH range 1 to 11. (c) Negative pH calibrations obtained from logistic function fitting of pH buffer and H_2_SO_4_ standards in the pH range −5 to 4, measured between 5 and 50 °C. pH electrode response. (d) Modeled temperature dependence of negative pH calibrations in the pH range −5 to 1. In all panels, calibration curves are color-coded by temperature (5 to 60 °C). Black curves indicate selected experimental temperature conditions, including the minimum and maximum experimental temperatures and two intermediate temperatures.

The standard calibration curves obtained between 6.6 and 57.8 °C (Figure 3a) show a systematic increase in electrode sensitivity with increasing temperature, consistent with the Nernst equation. Linear regression of the slope S against temperature gave a temperature coefficient of the Nernstian slope (*k*) = (1.99 ± 0.10)·10^–1^ mV·C^–1^·pH^–1^ with an offset (*C*) of −0.54 ± 3.21 mV·pH^–1^. Similarly, linear regression of the standard potential (*E*_0_) against temperature indicates a value of *E*_0_ at 25 °C of 418.9 ± 1.4 mV, with an experimentally determined temperature dependence of standard potential, $\frac{{dE}_{0}}{dT}$ = 1.13 ± 0.08 mV·C^–1^. eqs 13 and 14 were combined to generate a temperature-dependent calibration model for pH 1 to 10 (Figure 3b).

The negative pH calibration curves (Figure 3c) display a nonlinear response, with electrode potentials increasing systematically as temperature rises. Logistic functions fitted to the EMF-pH data at each temperature reveal that at higher temperatures, the curves plateau at progressively higher EMF values. To incorporate temperature effects, the logistic parameters (LP) were expressed as polynomial functions of temperature that were selected based on AICc (eqs 7 to 9), and the resulting coefficient values are summarized in Table 1.

**Table 1:** Values of Temperature-Dependent Polynomial Coefficients for the Logistic Parameters (LP) Used in the Negative pH Calibration Model^a^

LP	Logistic coefficient values	*R* ^2^
*A*(*T*)_3rd‑order_	*C*_3A_ = −1.87 ± 0.75·10^–3^, *C*_2A_ = 1.76 ± 0.62·10^–1^, *C*_1A_ = −3.49 ± 1.55, *C*_0A_ = 519 ± 12	0.95
*k*(*T*)_3rd‑order_	*C*_3k_ = 5.99 ± 2.01·10^–6^, *C*_2k_ = −5.32 ± 1.67·10^–4^, *C*_1k_ = 1.45 ± 0.42·10^–2^, *C*_0k_ = 3.61 ± 0.32·10^–1^	0.81
*x*_0_(*T*)_2nd‑order_	*C*_2x0_ = −2.41 ± 0.92·10^–4^, *C*_1x0_ = 1.14 ± 0.56·10^–2^, *C*_0x0_ = 2.65 ± 0.07	0.66

a Reported values are presented with their corresponding 95% confidence intervals.

Substitution of these temperature-dependent parameters into the logistic function yields a unified calibration model accounting for both temperature and pH (Figure 3d). Together, the Nernst-based and logistic calibration models can be applied to determine pH at any temperature within the experimental ranges (5 to 60 °C for the standard pH range and 5 to 50 °C for the negative pH range).

Figure 4 shows how CF changes with pH and with temperature. The CF decreases nonlinearly with increasing pH. At pH −5, CF values are highest, ranging from ∼190 to 220 mV, but decline steadily toward zero as pH approaches 1. This decreasing trend with pH is consistent across all temperatures. CF values are higher at higher temperatures. For example, at pH −5, the CF value at 48 °C exceeds 220 mV, whereas at 6 °C, it is only 188 mV, a difference of 32 mV. This temperature-dependent offset diminishes progressively with increasing pH, approaching nearly zero at pH 1.

**Figure 4:**
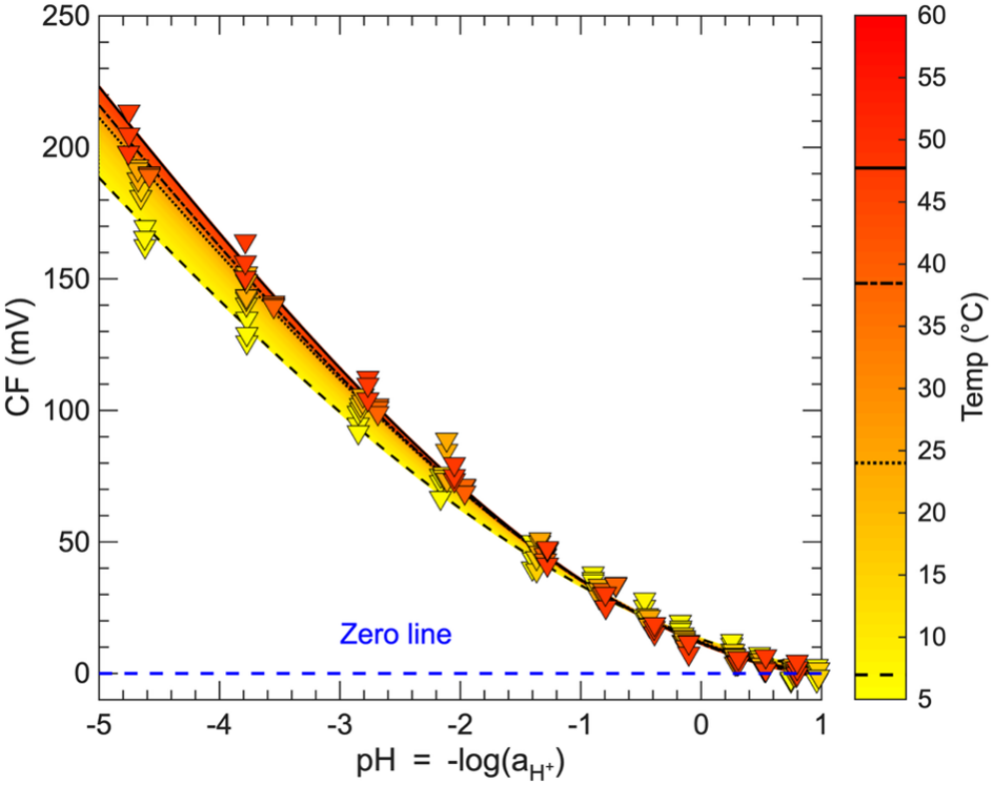
Correction factor (CF) for negative pH calibration as a function of pH (−5 to 1) and temperature (5 to 50 °C). Experimental data (▼, colored by temperature) are compared with modeled curves (shaded gradient). The dashed blue line indicates the zero CF reference level. Black curves indicate selected temperature conditions at 6.98, 24.01, 38.45, and 47.73 °C, similar to the selected experimental temperature conditions shown in negative pH calibration curves (Figure 3c,d).

### 3.3 Comparison of Nonlinear pH Calibration Curves for Different pH Glass Electrodes

The calibration curves for negative pH using the Metrohm Primatrode, Metrohm Unitrode, Orion Ross Sure-Flow, and Orion Ross Ultra pH/ATC Triode electrodes developed in this study, together with the Orion Ross pH electrode data from Nordstrom et al.,^1^ are presented in Figure 5. All calibration curves exhibit the characteristic nonlinear electrode response below pH 1. Most electrodes (Metrohm electrodes and the Orion Ross Sure-Flow electrode) retain sensitivity and continue to respond to proton activity as a measurable EMF signal down to pH < −4. Only the Orion Ross Ultra pH/ATC Triode electrode loses sensitivity below approximately pH −0.5.

**Figure 5:**
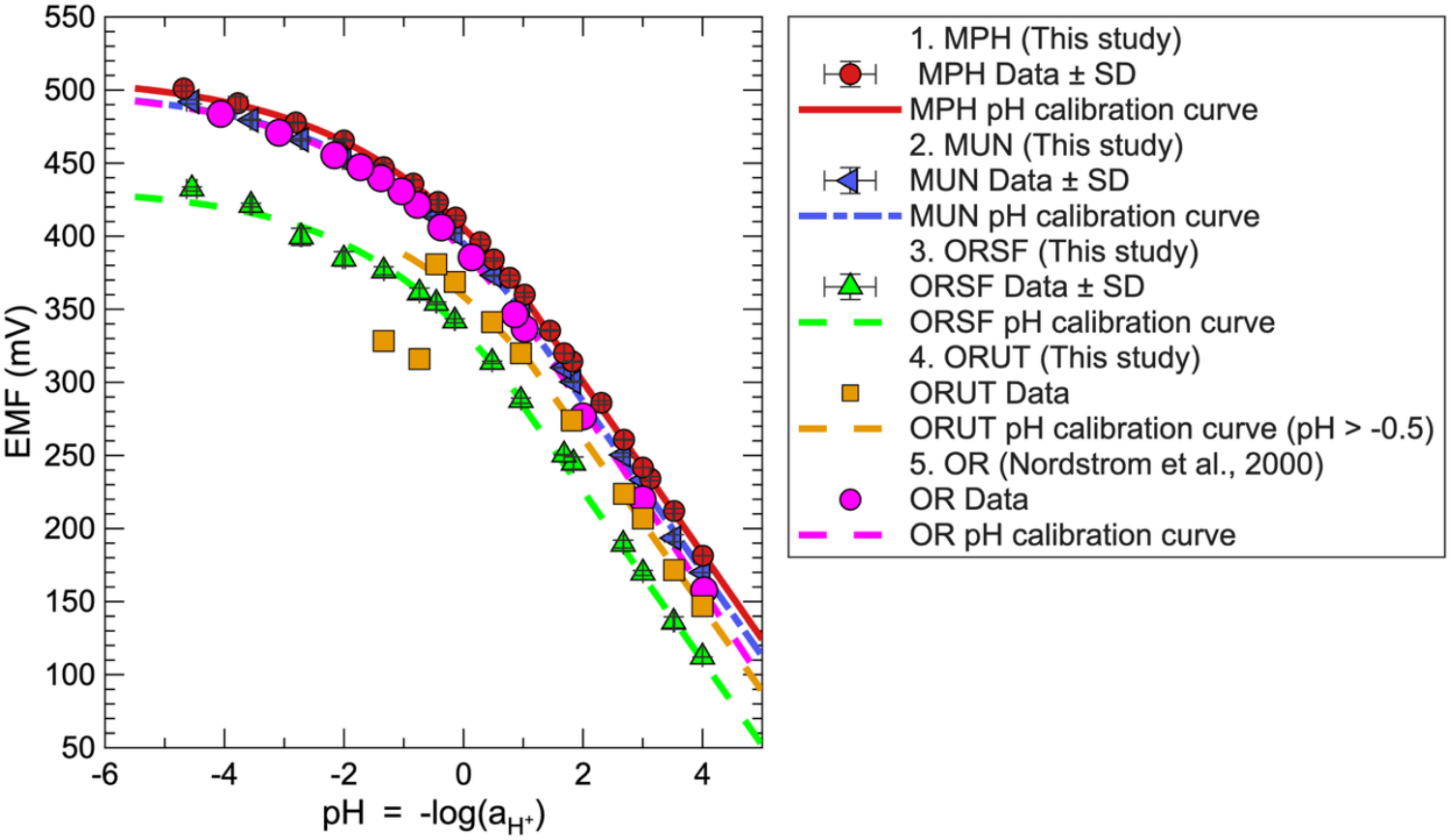
Comparison of pH electrode responses from this study and literature in the pH range −5 to 4. EMF-pH calibrations of 4 electrodes from this study, Metrohm Primatrode H = MPH (red circles, solid line), Metrohm Unitrode *N* = MUN (blue triangles, dashed line), Orion Ross Sure-Flow = ORSF (green triangles, dashed line) and Orion Ross Ultra pH/ATC Triode = ORUT (orange square), compared with Orion Ross = O data from Nordstrom et al. (2000) (magenta, circles, dashed line). Data points represent average EMF-pH values with standard deviations (SD) where available, and lines indicate the fitted calibration curves (logistic fit applied for pH < 1 and linear regression applied for pH ≥ 1). The negative pH calibration curve for the Orion Ross Ultra pH/ATC electrode was constructed excluding the data points at pH = −0.74 and −1.33, as the electrode had lost sensitivity in that range.

Comparison of the data from Nordstrom et al. (2000) with the calibration curve of the Primatrode electrode at equivalent pH values shows differences ranging from 8.8 to 21.7 mV. Differences with the Unitrode data are smaller, ranging from 4.1 to 12.5 mV (Figure 5). These differences in EMF measurements are consistent with standard pH calibration behavior described by the Nernst equation of each electrode, as summarized in Table 2, where different electrodes exhibit distinct *E*_0_ and slope values. In the same way that Nernstian calibration parameters differ between electrodes, negative pH calibration curves must also be determined individually for each electrode.

**Table 2:** Standard pH Calibration from 5 Different pH Glass Electrodes.^a^

Glass pH electrodes	EMF_Nernst_ pH calibration	*T* (°C)	% slope
Metrohm Primatrode (this study)	(418.0 ± 0.9) – (58.79 ± 0.12)·pH	24.4	99.6
Metrohm Unitrode (this study)	(403.2 ± 1.4) – (58.13 ± 0.18)·pH	24.6	98.4
Orion Ross Sure-Flow (this study)	(341.7 ± 10.2) – (57.98 ± 1.38)·pH	23.6	107.0
Orion Ross Ultra pH/ATC Triode (this study)	(377.6 ± 2.4) – (57.80 ± 0.33)·pH	24.3	97.9
Orion Ross (Nordstrom et al., 2000)	(401.6 ± 0.0) – (60.49 ± 0.00)·pH	25.0	102.3

a ± represents standard error (SE) estimated from LINEST function in Excel. For Orion Ross electrode, the standard calibration was derived from 2 data points at pH 4.03 & 3.00.

Variations in the proton activity coefficients (γ_H^+^_) have only a minor effect on the EMF-pH relationships compared to temperature and acid error. Nordstrom et al. reported a γ_H^+^_ value of 1200 for 9.85 mol·kg^–1^ H_2_SO_4_,^1^ whereas this study calculated a γ_H^+^_ value of 1095 for the same molality. Both studies used the Pitzer model with MacInnes scaling to determine proton activity coefficients in H_2_SO_4_ solutions.^29,30^ The difference between reported values (1200 vs 1095 at mol·kg^–1^ H_2_SO_4_) corresponds to only ∼0.04 pH units, indicating a negligible impact.

The Pitzer model is generally reliable for concentrations up to 6 mol·kg^–1^; beyond this range, calculations increasingly rely on extrapolated thermodynamic parameters, introducing additional uncertainty.^21,30^ As demonstrated by Spitzer et al., uncertainty in Pitzer coefficients alone contributes approximately ±0.03 pH units (95% confidence interval) in controlled systems such as seawater and a 0.05 mol·kg^–1^ acetic acid in KNO_3_.^31^ The difference in γ_H^+^_ values (i.e., 1200 and 1095) corresponds to a pH difference of approximately 0.04 units for a 10.11 mol·kg^–1^ H^+^ concentration in 9.85 mol·kg^–1^ H_2_SO_4_ solution. This 0.04 pH unit offset is minor compared to the observed 0.5 pH unit difference between the two calibration curves.

Temperature variations can also affect EMF measurements, but in this case, the effect is negligible because the measurements were conducted over a narrow temperature range. EMF measurements from Nordstrom et al. were reported at exactly 25.0 °C,^1^ whereas this study recorded EMF values over a temperature range of 22.8 to 24.5 °C, with an average temperature of 23.9 ± 0.4 °C. The slight deviation from 25 °C results from a temperature gradient between the room environment and the experimental setup. The pH electrode was rinsed with deionized water at room temperature and exposed to ambient air (16 °C) before being reimmersed in the test solution within a 25 °C water bath. The calculated impact of 0.5 to 2.2 °C difference on EMF measurements is only 0.11 to 0.43 mV per pH unit, based on the Nernst slope eq (2.303RT/F where *R* = 8.314 J·K^–1^·mol^–1^, *T* is temperature in K, and *F* = 96,485 C·mol^–1^). The small temperature difference (25 °C vs 23.9 ± 0.4 °C) cannot alone explain the observed EMF deviations, given the minor influence of temperature on electrode response.

The main factor influencing the differences between the 25 °C calibration curve from Nordstrom et al. and the 23.9 ± 0.4 °C calibration curve in this study is likely due to differences in electrode standard potential (*E*_0_) due to differences in electrode composition. This study used a Metrohm combined pH glass electrode (6.0228.020) with a 3 mol·L^–1^ KCl inner electrolyte, whereas Nordstrom et al. used an Orion Ross combined glass electrode with a 3.5 mol·L^–1^ KCl inner electrolyte and a Sargent-Welch combined glass electrode with a saturated 4.8 mol·L^–1^ KCl inner electrolyte.^1^ An *E*_0_ value of approximately 401 mV was estimated from Nordstrom et al.^1^ using two data points (pH 3 and 4) and a linear Nernst equation, whereas this study determined an *E*_0_ of 413 mV, indicating a 12 mV difference. Since calibration curves are electrode-specific, applying a calibration derived from one electrode to another can lead to pH inaccuracies. In this case, a 12 mV difference corresponds to an error of approximately 0.2 pH units, assuming a Nernstian slope of ∼59 mV·pH^–1^ at 25 °C. This highlights the importance of using electrode-specific calibration to ensure reliable pH measurements.

Only a single negative pH calibration could be completed for the Orion Ross Ultra pH/ATC triode electrode. The electrode showed a marked loss of sensitivity after EMF-pH measurements at pH −0.74 and −1.33, with EMF values substantially lower than expected. These data points were, therefore, excluded from the calibration curve. This loss of sensitivity behavior highlights that electrode performance in the negative pH range can be limited by a practical operating range.

Clear EMF-pH differences are observed among electrode types. EMF values of the Orion Ross Sure-Flow electrode differ from Primatrode data by approximately 63.7 to 80.3 mV, while EMF values of the Orion Ross Ultra pH/ATC Triode differ from the Primatrode by 41.7 to 43.5 mV. The lower measured potentials of the Orion Ross electrodes are attributed to electrode degradation caused by prolonged exposure to strong acids (up to 5 mol·L^–1^ H_2_SO_4_) and the absence of postacid conditioning in 3 mol·L^–1^ KCl, leading to progressive deterioration of the glass membrane.

Degradation of the Orion Ross electrode is supported by analysis of three diagnostic parameters: % slope, pH(0), and offset potential (dU) (Figure 6). Data from the Metrohm Primatrode electrode (October 2024 to May 2025), which was regularly conditioned in 3 mol·L^–1^ KCl, were compared with unconditioned data sets from the Orion Ross Sure-Flow (June 2023 to April 2025) and Orion Ross Ultra pH/ATC Triode (November 2022 to March 2025) electrodes, provided by Sun et al.^32^ The Primatrode exhibited stable performance, with % slope values of 98.2 ± 0.2% prior to calibration cycle number 18 and near-ideal values thereafter (99.3 ± 0.3%). The pH(0) and dU values (7.12 ± 0.02 and 7.1 ± 1.3 mV) showed no evidence of drift or loss of sensitivity. In contrast, both Orion Ross electrodes displayed substantial slope variability (94.5 to 101.2% and 93.2 to 98.5%), with several values outside acceptable limits, and progressive deviations in pH(0) and dU despite replacement of the inner electrolyte. This indicates that the observed drift primarily reflects irreversible electrode degradation rather than reference electrolyte contamination.

**Figure 6:**
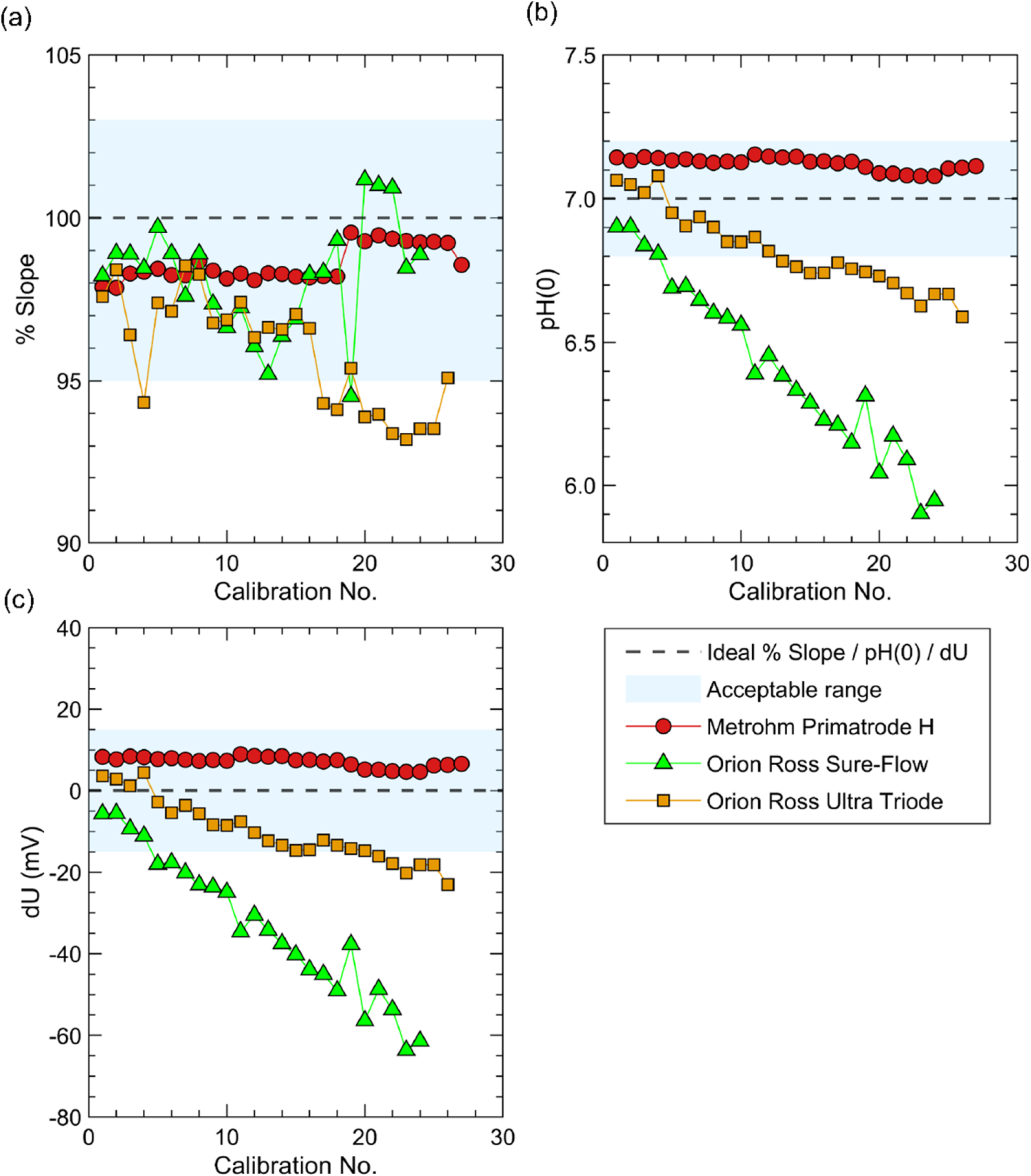
Monitoring the glass electrode performance using three diagnostic parameters: (a) % slope, (b) pH(0), and (c) offset potential (dU). The data are shown for the Metrohm Primatrode H (red circles), Orion Ross Sure-Flow (green triangles), and Orion Ross Ultra pH/ATC Triode (orange squares).

### 3.4 Performance Limit of pH Glass Electrodes under Extreme Acidity

In Section 3.3, it was shown that most electrodes (Metrohm electrodes and Orion Ross Sure-Flow electrode) retain the ability to detect proton activity as an EMF signal at negative pH values without losing sensitivity. In this section, the specific negative pH range in which the electrode can reliably measure EMF within the bounds of experimental error is evaluated. The theoretical sensitivity of the electrode is estimated as the derivative of EMF_Non–linear_ with respect to pH, representing how responsive the electrode potential is to changes in pH. This d­(EMF)/dpH value is compared with the experimental measurement uncertainty, expressed as the standard deviation (SD) of the EMF-pH measurements (mV). As shown in Figure 7, the SD of the measured data at each pH value (*n* = 3) for the Primatrode generally remains around 1.8 mV and less than 4 mV, but also higher at selected negative pH points (e.g., 8.5 mV at pH −2.11, 4.4 mV at pH −3.9, and 4.7 mV at pH −7.4). Between pH −2 and 0, the electrode maintains a theoretical sensitivity exceeding 20 mV·pH^–1^, comfortably above the level of experimental variability. At more extreme acidity (pH < −5), electrode sensitivity decreases, with |d­(EMF)/dpH| < 7 mV·pH^–1^ approaching the magnitude of the measurement uncertainty.

**Figure 7:**
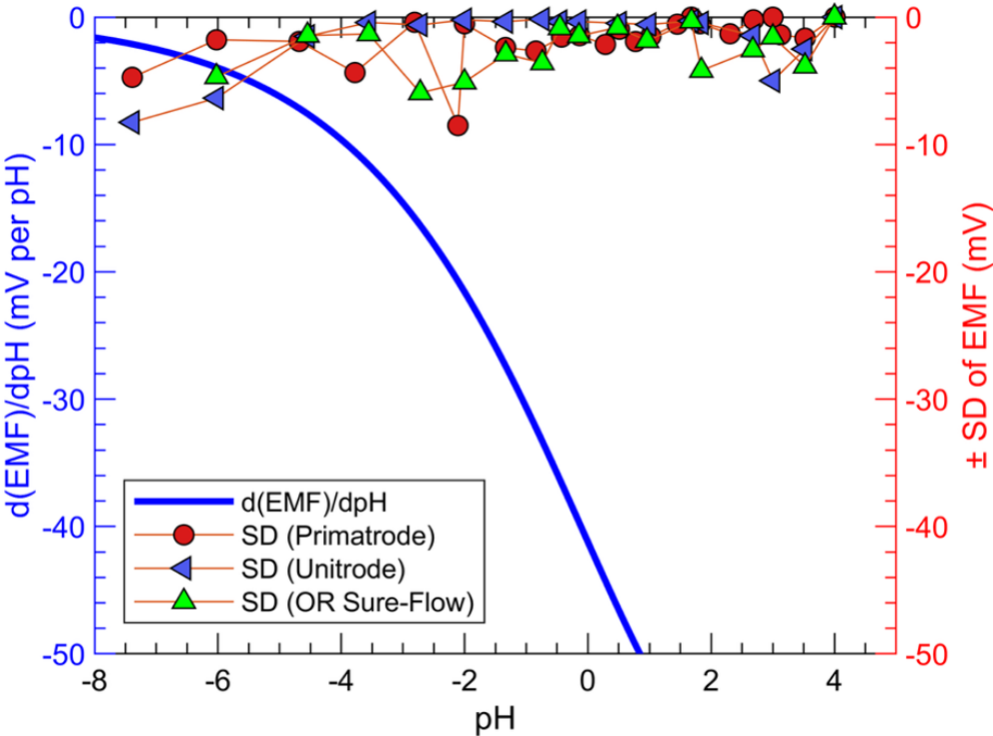
Comparison of theoretical electrode sensitivity and experimental measurement uncertainty. The derivative of EMF_Non–linear_ with respect to pH, shown as the solid blue line, represents the theoretical electrode sensitivity, while experimental measurement uncertainty is represented by the standard deviation (SD) of EMF measurements at each pH value (*n* = 3) for the electrodes from Primatrode (red circles), Unitrode (blue triangles), and Orion Ross Sure-Flow (green triangles) electrodes.

Similar results are obtained for the Unitrode electrode and the Orion Ross Sure-Flow electrode with mean EMF standard deviations of around 1.6 and 2.5 mV, respectively. The variability in SD of EMF measurements increases at lower pH values, reaching 6.4 mV at pH −6.0 and 8.3 mV at pH −7.4 for the Unitrode. These findings indicate that, as pH decreases, electrode sensitivity becomes small and of similar magnitude to measurement error below a pH of around −5. Below pH −5 to −7.4, the electrode continues to produce a measurable EMF response; however, its sensitivity to changes in pH decreases with increasing acidity. As a result, calibration in this region is not recommended due to the large associated uncertainties in pH determination.

### 3.5 Breakdown of Linear Calibration and Acid Error Estimation in the Negative pH Range

The calculated acid error resulting from the application of the standard linear pH calibration method to the nonlinear electrode response in H_2_SO_4_ solutions is shown in Figure 8. Traditional pH calibration assumes a linear relationship between the EMF and pH. However, in highly acidic solutions (pH < 1), reliance on the standard linear calibration leads to a consistent overestimation of the true pH values. At pH −1, the acid error is +0.6 pH units, and at pH −5, the error exceeds +3.5 pH units.

**Figure 8:**
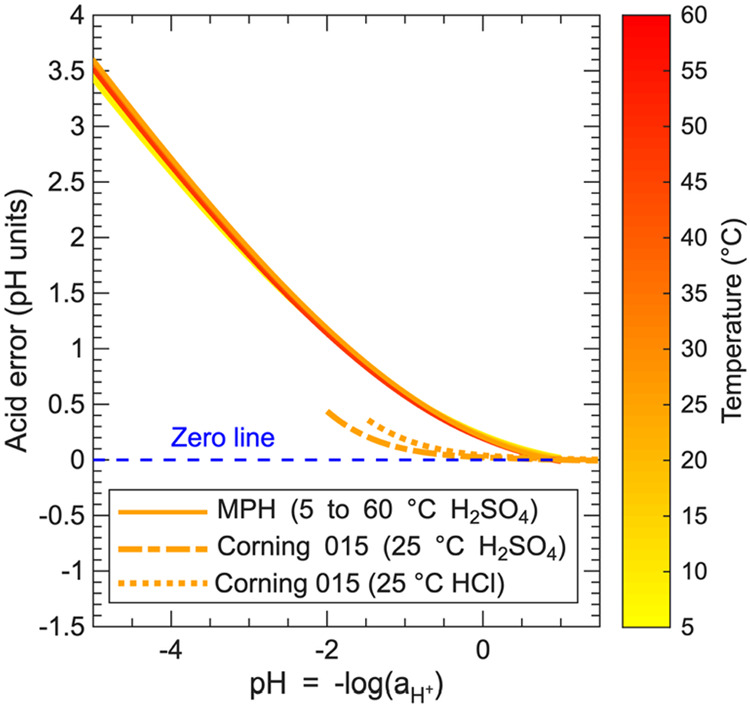
Comparison of acid error across different pH electrodes in the negative pH range. The acid error (pH units) is shown for the Metrohm Primatrode H (MPH) in H_2_SO_4_ between 5 and 65 °C (color-gradient lines, this study), and compared with literature data for the Corning 015 electrode in H_2_SO_4_ (orange dash-dot line) and HCl (orange dotted line) at 25 °C.

Earlier studies reported acid errors of approximately 0.5 pH units at pH −2 for pH measurements conducted using the Corning 015 glass electrode in H_2_SO_4_.^12,16,33^ In these studies, acid error was determined by direct comparison to a hydrogen electrode, which serves as a thermodynamic reference for proton activity.^34,35^ In this present study, the acid error is defined by comparing linear and nonlinear calibrations obtained from the same electrode, thereby isolating deviations of the measured electrode potential from ideal Nernstian behavior. Despite this methodological difference, the Metrohm combined glass electrode used here exhibited an acid error exceeding +1 pH unit at pH −2, more than double the value reported for the Corning 015 electrode under the same conditions at 25 °C in H_2_SO_4_ standards. The onset of acid error also varied across studies: Bates (1973) observed it at pH < 0, Nordstrom et al. at pH < 0.5, and the results presented in Figure 8 suggest an onset at pH < 1.^1,16^ These discrepancies suggest that differences in glass electrode type may influence both the magnitude and the onset of acid error.

While alternative electrodes could be used to measure proton activity, studies show that acid error at very low pH is not limited to glass membranes.^36^ Nonideal behavior has also been reported for alternative proton-sensing electrodes based on hydrogen-storage palladium (Pd).^36^ The equilibrium potentiometric pH measurements from the Pd electrode are also challenged under strongly acidic conditions.^36^ Since glass electrodes remain the most widely used and cost-effective option, implementing robust, calibration-based corrections for acid error is essential.

### 3.6 Thermodynamic Origins of Nonlinearity in Negative pH Measurements

The observed nonlinearity of the EMF response curve in the negative pH range reflects the combined influence of electrode considerations and solution thermodynamics on the pH scale. Because pH is defined in terms of proton activity rather than concentration, and because the proton activity coefficient (γ_H^+^_) deviates substantially from unity at high ionic strength, the pH scale becomes inherently nonlinear at high proton concentrations.^23,29^ Although the nonlinear electrode response can be corrected empirically using a logistic calibration function (as outlined in Sections 3.1 to 3.3), it fundamentally arises from thermodynamic effects governing proton activity under highly acidic conditions as expressed in eq 15

15 $$\mathrm{pH}=-\log{(a}_{H^{+}})=-\log\left(\gamma_{H^{+}}{\cdot m}_{H^{+}}\right)$$

which can be decomposed as

16 $$\mathrm{pH}=-\log\left(\gamma_{H^{+}}\right)-\log\left(m_{H^{+}}\right)$$

The dependence of proton activity and its activity coefficient on proton concentration in pure H_2_SO_4_ and mixed H_2_SO_4_–FeCl_2_ systems was investigated using thermodynamic modeling. In dilute solutions, where γ_H^+^_ approaches unity, the first term in eq 16 is negligible and proton activity scales approximately linearly with concentration. However, at high ionic strength, significant deviations from ideality occur. As shown in Figure 9a, γ_H^+^_ increases nonlinearly with increasing proton molality (*m*_H^+^_). This behavior reflects the growing importance of short-range ion–ion interactions and ion pairing at high concentrations, effects that are not adequately captured by classical Debye–Hückel theory, which is generally valid only below ionic strengths of ∼0.1 mol·kg^–1^.^29,37^ In contrast, the Pitzer ion-interaction model explicitly accounts for these higher-order interactions through virial-type coefficients and has been validated for concentrated electrolyte systems including H_2_SO_4_ solutions up to 6 mol·kg^–1^.^23,38^ In pure H_2_SO_4_ systems, the ion interactions are further complicated by the equilibrium among H^+^, SO_4_^2–^, and HSO_4_^–^, which reduces the effective free proton population and modifies the activity coefficient relative to a fully dissociated acid of equivalent molality.^23,39^

**Figure 9:**
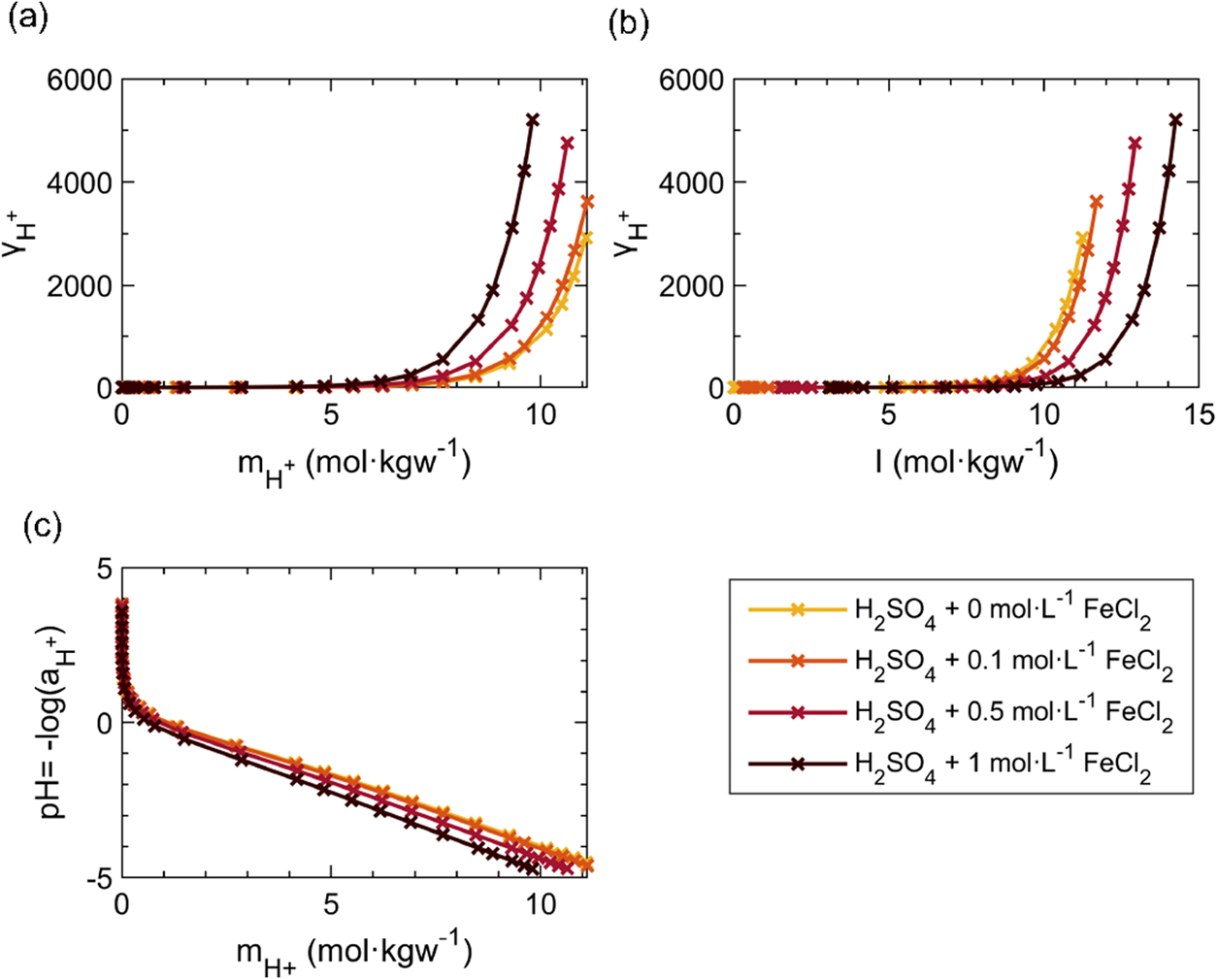
Dependence of proton activity and its proton activity coefficient on high ionic strength in pure H_2_SO_4_ and mixed H_2_SO_4_–FeCl_2_ systems. Results from PHREEQC (Pitzer.dat) calculations showing the proton activity coefficient (γ_H^+^_), proton molality (*m*_H^+^_, mol·kg^–1^), ionic strength (*I*, mol·kg^–1^), and pH = −log in H_2_SO_4_ solutions containing 0, 0.1, 0.5, and 1 mol·L^–1^ FeCl_2_. (a) γ_H^+^_vs *m*_H^+^_. (b) γ_H^+^_vs I. (c) pH vs *m*_H^+^_.

In Figure 9b, the strong increase in γ_H^+^_ with high ionic strength highlights the nonideal behavior of concentrated electrolyte solutions, in which electrostatic screening and specific ion interactions significantly alter thermodynamic properties.^29,38^ The addition of FeCl_2_ further enhances this effect by increasing the total ionic strength and introducing divalent Fe^2+^ ions, which contribute disproportionately to interionic interactions due to their higher charge.^40^ As a result, systems containing higher FeCl_2_ concentrations exhibit systematically higher proton activity coefficients at a given proton molality.

The combined effects of these interactions that lead to nonideal behavior is illustrated in Figure 9c, which shows the relationship between pH = −log (*a*_H^+^_) and proton molality. Unlike the linear relationship expected under ideal conditions, the pH–*m*_H^+^_ relationship is nonlinear. This deviation arises because pH depends on both concentration and activity coefficient (eqs 15 and 16): the decomposition in eq 16 explicitly separates the nonideal contribution, log (γ_H^+^_), from the concentration contribution, log (*m*_H^+^_). At high acid concentrations, the rapid increase in γ_H^+^_ offsets the increase in *m*_H^+^_, resulting in a nonlinear pH scale and reducing sensitivity of pH to changes in proton concentration.^2,38^ This effect becomes increasingly pronounced at higher ionic strengths and in the presence of FeCl_2_, consistent with the trends in Figure 9a–c.

The thermodynamic results demonstrate that in highly concentrated sulfuric acid systems, proton activity is governed not only by proton concentration but also, critically, by nonideal interactions captured in the activity coefficient. This nonideal behavior is reflected in both the nonlinear response of glass pH electrodes and the reduced EMF sensitivity at low pH, consistent with observations in highly acidic natural and industrial systems.^1,41^

### 3.7 Application of Negative pH Calibration to Real-World Acidic Systems

The application of negative pH calibration to extremely acidic solutions, compared with standard linear Nernst calibration, is shown in Figure 10. pH values calculated using the standard linear Nernst equation increasingly deviate as pH becomes more negative, consistent with the acid error trends in Figure 8. For example, a true pH of −3.5 in 6 mol·L^–1^ H_2_SO_4_ is measured as −1.3, corresponding to an overestimation of approximately +2.3 pH units.

**Figure 10:**
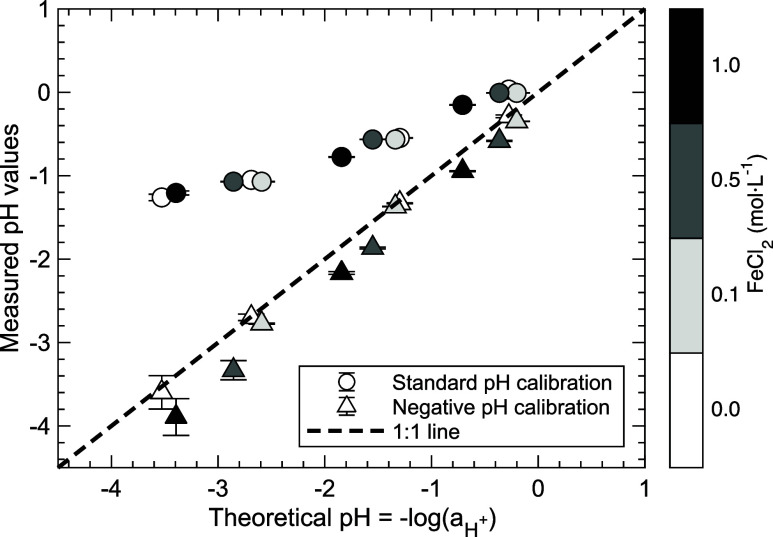
Comparison of measured pH values obtained using standard pH calibration and negative pH calibration following Sections 3.1 and 3.2. Measured pH is plotted against theoretical pH in FeCl_2_ solutions across different concentrations (color-coded, 0.1, 0.5 and 1.0 mol·L^–1^). The dashed line represents the 1:1 relationship between theoretical and measured pH. Circles denote standard calibration and triangles denote negative calibration. Data represent averages of triplicate measurements, with error bars showing standard deviations (SD, *n* = 3).

This acid error is substantially reduced after applying a nonlinear negative pH calibration based on a logistic function. In H_2_SO_4_ solutions (white markers in Figure 10), measured pH values remain close to the true values, although uncertainties increase at more negative pH, as reflected by larger errors. The pH error in 5 mol·L^–1^ H_2_SO_4_ is reduced from +1.6 to 0.0 pH units when using the logistic calibration compared to linear calibration, and in 6 mol·L^–1^ H_2_SO_4_, the pH error is reduced from +2.3 pH units to −0.5 units. These results demonstrate that pH measurements with glass electrodes can be significantly improved under extremely acidic conditions when paired with an adequate calibration protocol (standards + calibration model).

When the negative pH calibration was applied to synthetic AMD samples (H_2_SO_4_ + FeCl_2_), deviations from the 1:1 line increase systematically with FeCl_2_ concentration (gray to black points = lower to higher FeCl_2_ concentrations in Figure 10). For example, in a 1 mol·L^–1^ H_2_SO_4_, solution deviations from the 1:1 line are 0.01, 0.15, 0.22, and 0.24 pH units for 0, 0.1, 0.5, and 1.0 mol·L^–1^ FeCl_2_, respectively. Similarly, in 5 mol·L^–1^ H_2_SO_4_ solution, deviations are 0.01, 0.18, 0.48, and 0.50 pH units for 0, 0.1, 0.5, and 1.0 mol·L^–1^ FeCl_2_. These trends are not attributed to limitations of the H_2_SO_4_-based calibration itself but instead arise from matrix-dependent effects. Differences in ionic strength and solution composition between calibration standards and mixed solutions alter proton activity coefficients, leading to higher H^+^ activity in FeCl_2_-containing systems compared to pure H_2_SO_4_ solution.^23,40^ Pitzer-MacInnes calculations confirm that the addition of FeCl_2_ increases ionic strength, proton activity coefficients, and proton activity, which results in lower (more negative pH) pH values at the same given total proton concentration compared with H_2_SO_4_ standards (Figure 9).

In pure H_2_SO_4_ solutions, liquid junction potential (LJP) effects are inherently accounted for within the nonlinear H_2_SO_4_-based calibration. However, because LJP depends on solution composition and ionic strength, different LJP values are expected in mixed matrices such as the mixed H_2_SO_4_ + FeCl_2_ solutions where ionic strength and ionic composition differ from those of the calibration standards.^43,44^ As a result, LJPs affecting calibration and sample measurement are different, leading to pH-EMF offsets.^44,45^ While the magnitude of LJP in the negative pH range is currently unknown, studies at pH 3.5 demonstrate that LJP effects can contribute uncertainties of approximately 0.10 to 0.14 pH units.^42,44^ Other studies estimated LJP of +0.03 pH units at pH > 0.5 and −0.2 pH units for more acidic samples.^1^ Matrix-specific calibration approaches may be required for a more accurate pH determination in chemically complex acidic solutions.^41,46^ Further studies examining the combined effects of ionic strength and proton activity on glass electrode EMF responses across a wider range of solution compositions are needed to improve and generalize negative pH calibrations.

Overall, calibration standards using H_2_SO_4_ is a robust and practical starting point for pH measurements in extremely acidic systems, as demonstrated in Figure 10.. Compared with conventional linear Nernst calibration,, this approach significantly improves accuracy compared with conventional NIST or DIN buffer-based calibrations, which are limited to minimum pH values of 1.679 (NIST, 25 °C) and 1.09 (DIN, 25 °C).^46^

## 4 Conclusions

This study presents an experimental protocol for measuring proton activities using glass electrodes, extending a calibration to negative pH values down to pH −7.4 at 25 °C and providing a continuous and reliable calibration model from pH 1 to −5 across a temperature range of 5–60 °C. It establishes a temperature-dependent, nonlinear calibration strategy that corrects for acid error and improves the accuracy of glass electrode measurements in strong sulfuric acid systems.

The results show that glass electrode behavior remains close to Nernstian above pH 1. Below this pH threshold (pH < 1), the electrode response becomes progressively nonlinear and the correction factor increases rapidly, leading to large systematic overestimation of pH when conventional calibration is applied. At pH −1, the error is approximately +0.6 pH units and exceeds +3 pH units by pH −5.

A negative pH calibration protocol was therefore developed using a four-parameter logistic model to describe the electrode potential as a function of pH and temperature (eq 17)­

17 $${\mathrm{EMF}\left(\mathrm{pH},T\right)}_{\mathrm{Non}-\mathrm{linear}}=\frac{{A\left(T\right)}_{3\mathrm{rd}-\mathrm{order}}}{1+e^{-{k\left(T\right)}_{3\mathrm{rd}-\mathrm{order}}\cdot \left(\mathrm{pH}-{x_{0}\left(T\right)}_{2\mathrm{nd}-\mathrm{order}}\right)}}$$

The temperature dependence of the logistic parameters was expressed using polynomial functions, with coefficients reported together with their 95% confidence intervals as follows:

- *A*(*T*)_3rd‑order_ = *C*_3A_·*T* ^3^ + *C*_2A_·*T* ^2^ + *C*_1A_·*T* + *C*_0A_ and parameter coefficients are *C*_3A_ = −1.87 ± 0.75·10^–3^, *C*_2A_ = 1.76 ± 0.62·10^–1^, *C*_1A_ = −3.49 ± 1.55, *C*_0A_ = 519 ± 12.
- *k*(*T*)_3rd‑order_= *C*_3k_·*T* ^3^ + C_2k_·*T* ^2^ + C_1k_·*T* + *C*_0k_ and parameter coefficients are *C*_3k_ = 5.99 ± 2.01·10^–6^, *C*_2k_ = −5.32 ± 1.67·10^–4^, *C*_1k_ = 1.45 ± 0.42·10^–2^, *C*_0k_ = 3.61 ± 0.32·10^–1^.
- *x*_0_(*T*)_2nd‑order_ = *C*_2 x0_·*T* ^2^ + *C*_1x0_·*T* + *C*_0x0_ and parameter coefficients are *C*_2x0_ = −2.41 ± 0.92·10^–4^, *C*_1x0_ = 1.14 ± 0.56·10^–2^, *C*_0x0_ = 2.65 ± 0.07

The unified logistic model provides a practical pH calibration that captures the nonlinear electrode response and improves pH measurement accuracy in the negative pH region, where traditional linear Nernstian calibration becomes unreliable. It reduces acid error from +1.6 to 0.0 pH units in 5 mol·L^–1^ H_2_SO_4_.

While the model was developed using H_2_SO_4_ standards at different temperatures, the results demonstrate the broader importance of accounting for ionic strength and solution composition when performing pH measurements under highly acidic conditions. Rather than limiting the applicability, these findings establish the foundation for extending the approach to other strongly acidic systems through targeted matrix-specific calibration. Future work should therefore focus on quantifying matrix effects to further generalize and expand reliable negative pH measurement across diverse chemical environments.

## Supplementary Material



## Notes

Descriptions of the pH glass electrodes used in this study, calibration procedures, and conditioning protocols for negative pH measurements. It includes preparation and standardization of H_2_SO_4_ solutions, potentiometric titration data, and theoretical pH calculations based on proton activities using the PHREEQC program with the Pitzer model. Additional EMF-pH data sets, electrode performance diagnostics, nonlinear calibration models, temperature corrections, and uncertainty analyses are also presented (PDF). All underlying data and the analysis scripts used to generate the calibration models are freely available in the GitHub repository at https://github.com/SarawudTH/NegativePH
