# Supplementary material for: Temperature-Dependent Nonlinear Calibration of Glass pH Electrodes for Negative pH Applications
Source: ACS Omega. 2026 Jul 2;11(27):40120–33. doi: 10.1021/acsomega.6c01903 (PMC13382843; doi:10.1021/acsomega.6c01903)
Supplement: Supplementary file 1 [file ao6c01903_si_001.pdf]

Supplementary information

Temperature-Dependent Nonlinear Calibration of Glass pH Electrodes for Negative pH Applications

Sarawud Saleesongsom, Dominik Weiss*, Yves Plancherel
Department of Earth Science and Engineering, Imperial College London, Royal School of Mines, Prince Consort Road, London SW7 2AZ, United Kingdom
*Corresponding author: d.weiss@imperial.ac.uk

Table of Contents

		Page
S1	Combined pH glass electrodes used in the study	2
S2	Commercial pH buffers and temperature correction	6
S3	Experimental protocol for negative pH calibration of a glass pH electrode	7
S4	Preparation of H_2SO_4 Standard Solutions	9
S5	Potentiometric Titrations of H_2SO_4 Standard Solutions Against 0.1 mol·L^{-1} NaOH	10
S6	Proton Activity Calculations Using the Pitzer Model and the MacInnes Assumption in PHREEQC	13
S7	EMF Measurements of pH standards (H_2SO_4 solutions and Hanna pH buffers)	18
S8	pH glass electrode performance monitoring	34
S9	Negative pH Calibration Curve fitting Using a Logistic Function	41
S10	Logistic fit uncertainties analysis using Monte Carlo simulation	42
S11	Effect of Temperature on Electrode Response	43
S12	Different non-linear calibration from different electrodes	48

S1 Combined pH glass electrodes used in the study

6 combined pH glass electrodes were used in this study (Fig. S1 and Table S1). 4 Metrohm electrodes are sourced from the Environmental Geochemistry Laboratory at Imperial College London, South Kensington campus, and 2 Orion Ross electrodes from Kucernak Group at Molecular Sciences Research Hub, White City Campus. Each electrode has slightly different specifications, mainly in temperature range, type of temperature sensor, and electrode body. All 6 electrodes use the same reference system (Ag/AgCl) and reference electrolyte in KCl 3 mol·L^{-1}.

Electrodes H and I were Metrohm Primatrode models (now discontinued). Electrode H was the primary probe used across different acid concentrations and temperatures, but after losing sensitivity on 6 June 2025, it was replaced by electrode I for negative pH experiments at 40 °C and 65 °C. Electrode N was used for potentiometric titration of H_2SO_4 until it broke on 8 November 2024. Electrode I was then used as its replacement. Electrode R, with the same specifications as N, was later used only for negative pH studies. The two Orion Ross electrodes were only used for negative pH calibration at 25 °C.

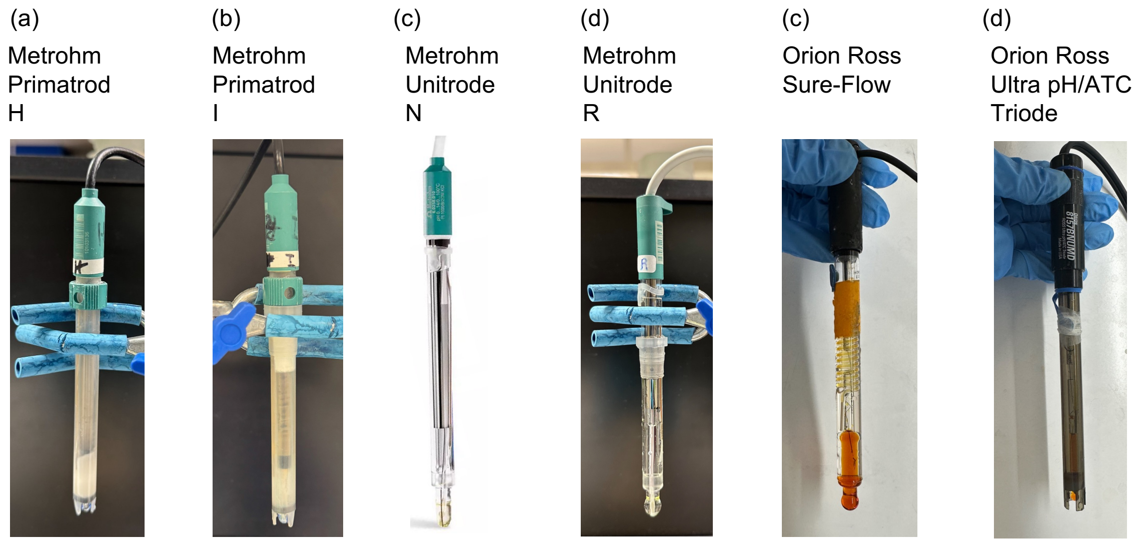

Figure S1 *Metrohm and Orion Ross combined pH glass electrodes used in this study. The image of Electrode N was sourced from www.metrohm.com, accessed on 29 July 2025.*

When using a combined pH glass electrode with very low and negative pH solutions, additional care is required. the electrode should be conditioned in 3 mol·L^{-1} KCl after the measurement to restore its performance. Exposure to highly acidic environments can alter the hydration layer on the glass membrane and affect the reference junction, leading to unstable responses in subsequent measurements. After removing the electrode from the

negative pH solution, it should be rinsed thoroughly with distilled water and then soaked in fresh 3 mol·L^{-1} KCl. The conditioning process may need to be repeated, replacing the KCl solution multiple times, until the electrode consistently measures the pH of the 3 mol·L^{-1} KCl solution at the same value as before exposure to the negative pH solution. This procedure ensures that the electrode has fully recovered and is ready for accurate use again (Fig. S2).

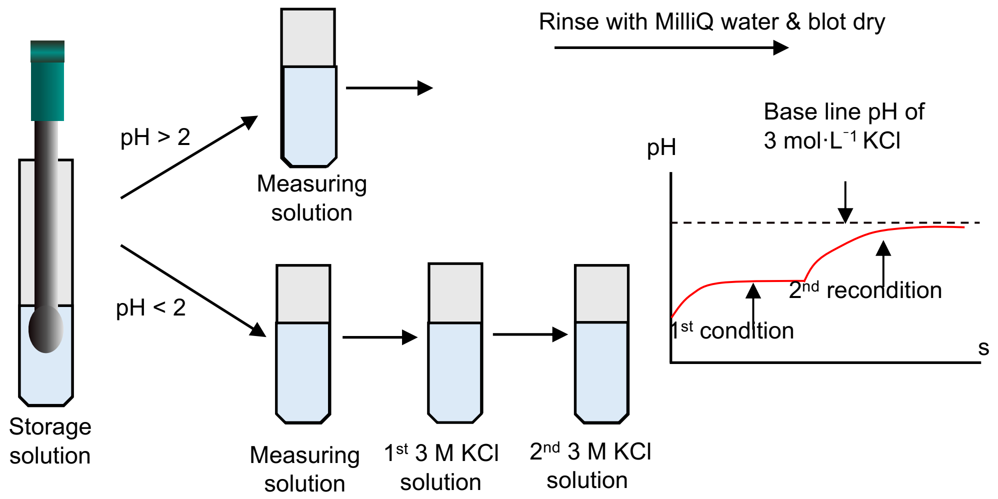

Figure S2 *Schematic illustration of the additional conditioning step for negative pH calibration. pH electrodes used for very low and negative pH measurements must be stored in fresh 3 mol·L^{-1} KCl and repeated as needed to avoid cross contamination for subsequent measurements.*

Table S1 *Specification and experimental application of Metrohm combined pH glass electrodes used in this study. The manufacturer specifies a working pH range of 0 to 14 for these electrodes. While operation outside this range is possible, it requires additional procedures and corrections which are described in this study.*

Electrode	Metrohm				Orion Ross	
	Primatrode H	Primatrode I	Unitrode N	Unitrode R	Sure-Flow	Ultra pH/ATC Triode
1. Specification						
Catalog number	6.0228.020	6.0228.020	6.0258.010	6.0258.010	8172BNWP	8157BNUMD
pH Range	0-14	0-14	0-14	0-14	0-14	0-14
Operating temperature (°C)	0-80	0-80	0-100	0-100	0-100	0-100
Temperature sensor	NTC	NTC	Pt1000	Pt1000	No	No
Diaphragm/Junction	Ceramic	Ceramic	Fixed ground-joint	Fixed ground-joint	Na	Glass Fiber
Reference electrolyte	KCl 3 mol·L^{-1}	KCl 3 mol·L^{-1}	KCl 3 mol·L^{-1}	KCl 3 mol·L^{-1}	KCl 3 mol·L^{-1}	KCl 3 mol·L^{-1}
Body material	Polypropylene	Polypropylene	Glass	Glass	Glass	Epoxy
2. Experimental Application						
Negative pH calibration	Yes	Yes (40 & 60°C)	No	Yes, only at 25°C	Yes, only at 25°C	Yes, only at 25°C
Potentiometric Titration	No	Yes	Yes	No	No	No

Table S2 *Comparison of pH meters and electrodes reported to measure negative pH and pH > 14. Most of these pH electrodes are calibrated using 3-point or 5-point calibration methods, typically based on standard pH buffers provided by the National Institute of Standards and Technology (NIST), USA, with values such as pH 1.68, 4.01, 6.87, 7.01, 9.18, 10.01, & 12.45. Note that all significant figures reported in this table come from the company report.*

Company	pH meter model	pH electrode model	pH range	pH accuracy
Hanna Instruments	HI-2020 Edge Hybrid Multiparameter pH, EC, DO Meter	HI 11310	-2.00 to 16.00	±0.01
	HI-2002 Edge pH Meter	HI-11310	-2.00 to 16.00	±0.01
	HI-98190 Professional Waterproof pH/ORP Meter	HI-12963	-2.0 to 20.0	±0.1
Apera Instruments	SX723 Portable pH/Conductivity Meter	201T-S	-2.00 to 19.99	±0.01
	ZenTest PCO60-Z Smart Multi-Parameter	PC60-DE	-2.00 to 16.00	±0.01
	PH8500-HT Portable pH Meter	LabSen 863	-2.00 to 16.00	±0.01
	PH60 Pocket pH Tester pH Meter	PH60-E	-2.00 to 16.00	±0.01
Horiba	LAQUA PH1500	9625-10D	-2.00 to 16.00	±0.01
Oakton	pH 700 pH/mV/°C/°F Bench Meter	ECFG7370101B	-2.00 to 16.00	±0.01
Mettler Toledo	SevenExcellence pH meter S400	InLab® Expert Pro-ISM	-2.000 to 20.000	±0.002
	Seven2Go pH /mV meter S8	InLab® Expert Pro-ISM-IP67	-2 to 20	±0.01
Thermo Scientific	Orion Star A211 pH Benchtop Meter	8172BNWP	-2.000 to 20.000	±0.002
Jenway	3510 Standard Digital pH Meter	924 005	-2.000 to 19.999	±0.003
Xylem	inoLab pH 7110	SenTix® 41	-2.000 to 19.999	±0.005
Ohaus	Starter 3100 pH Bench	ST310	-2.00 to 16.00	±0.01
Crison	Multimeter MM 40	5048	-2.0 to 16.00	±0.01

S2 Commercial pH buffers and temperature correction

In this study, commercial Hanna buffer solutions were used for pH calibration. All buffer solutions were used according to the manufacturer's specifications and were traceable to NIST standards. For standard 3-point calibration, the following buffer solutions were used: pH 4.01 (HI-7004L/C), pH 7.01 (HI-7007L/C), and pH 10.01 (HI-7010L/C). These buffers were selected to ensure accurate calibration across a wide pH range and to allow extension of the calibration down to pH 2 and up to pH 12. For very low and negative pH calibration, Hanna buffer solutions with pH values of 1.68 (HI-6001) and 3.00 (HI-6002) were used and compared against standard sulfuric acid (H_2SO_4) solutions prepared in this study. Buffer solutions at pH 12 and 13 were only used for calibration in strongly alkaline conditions (pH > 12). While this part of the work is not the main focus of the negative pH study, high-alkaline calibration and how logistic functions can be applied to both negative and very high (pH > 14) values are included on the main discussion. This is meant to support and encourage future research in this area.

The actual pH of commercial buffer solutions varies with temperature, and this effect must be accounted for during calibration to ensure accurate pH determination. Each Hanna buffer solution has a known temperature-pH dependence and failure to correct for this variation can lead to systematic errors, particularly when measurements are performed at temperatures significantly different from 25 °C. During calibration, temperature compensation was applied using manufacturer-provided correction data, with the temperature dependence of each buffer solution.

S3 Experimental protocol for negative pH calibration of a glass pH electrode

Fig. S3 illustrates the overall experimental workflow used to establish a negative pH calibration for a glass electrode. Concentrated sulphuric acid was diluted to generate a series of acidic standards spanning positive to strongly negative pH values, complemented by conventional buffer solutions for cross-calibration. The molar concentration of each acid standard was determined independently by titration against sodium hydroxide. Electrode potentials were measured under tightly controlled thermal conditions, ensuring signal stability prior to data acquisition. Measured EMF values and corresponding temperatures were combined with acid concentrations in PHREEQC to compute thermodynamic pH values using charge-balance constraints. These data were subsequently integrated to derive a continuous EMF-pH-temperature calibration, parameterised using a logistic function to account for non-linear electrode response at negative pH. Full methodological details for each step are provided starting from Section S4: Preparation of H_2SO_4 Standard Solutions.

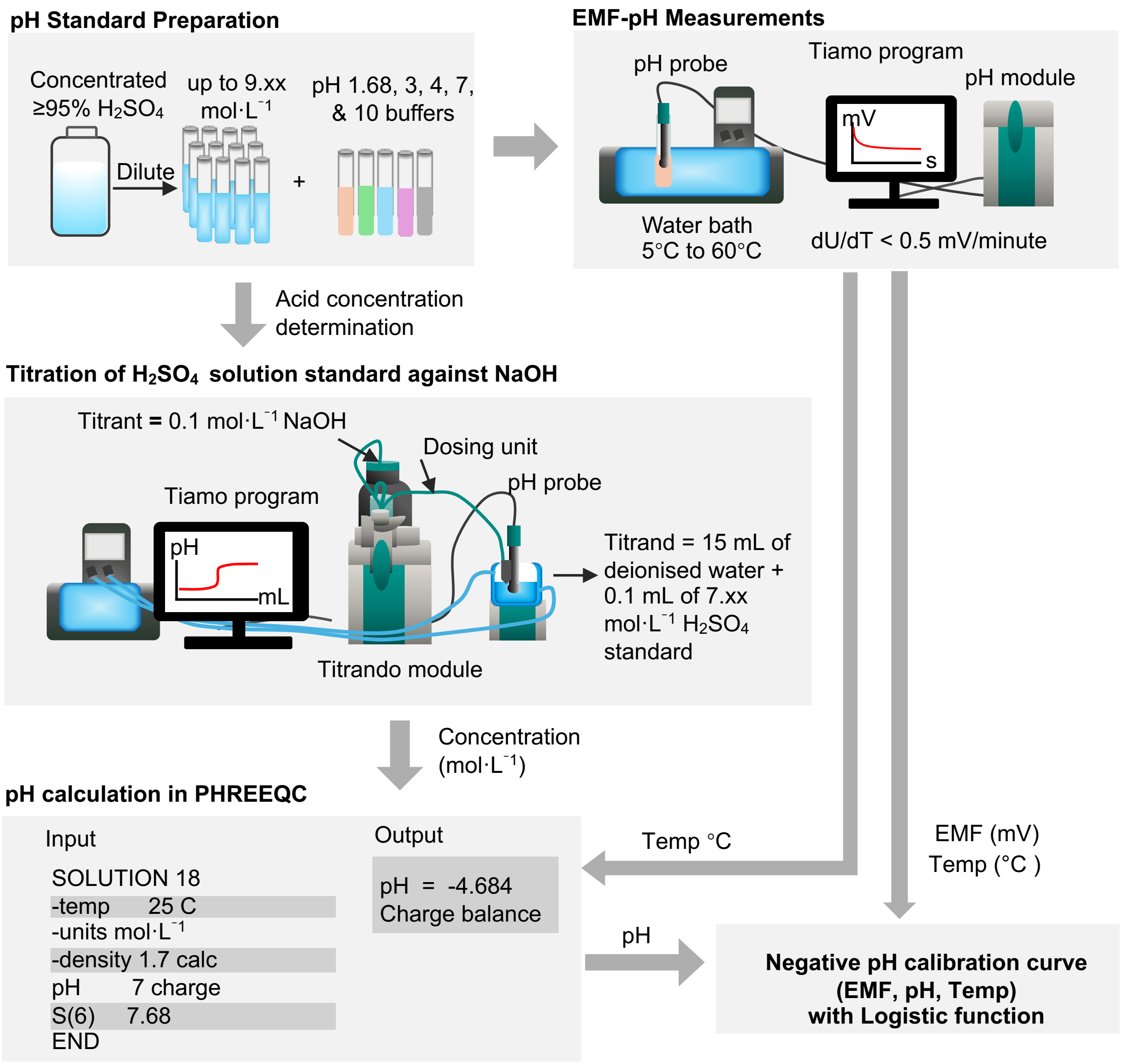

Figure S3 *Experimental protocol for negative pH calibration of a glass pH electrode.*

S4 Preparation of H_2SO_4 Standard Solutions

Sulfuric acid calibration standards were prepared by diluting concentrated H_2SO_4 (≥95%, ≈17.82 mol·L^{-1}, VWR) with Milli-Q water (Table S3). Due to the high viscosity of concentrated H_2SO_4, pipetting can be imprecise, especially when transferring larger volumes with a 5 mL pipette, which may result in higher-than-intended concentrations.

Table S3 *Preparation of diluted H_2SO_4 solutions from concentrated 95% H_2SO_4.*

ID	Expected H_2SO_4 mol·L^{-1}	Volume Milli-Q water (mL)	Volume 95% H_2SO_4 (17.8 mol·L^{-1}, mL)	Total volume (mL)
1_0.0001M	0.0001	499.997	0.003	500
1_0.00032M	0.00032	499.991	0.009	500
1_0.001M	0.001	499.972	0.028	500
1_0.0032M	0.0032	499.910	0.090	500
1_0.01M	0.01	29.983	0.017	30
1_0.032M	0.032	99.820	0.180	100
1_0.1M	0.1	29.831	0.169	30
1_0.18M	0.18	98.989	1.011	100
1_0.32M	0.32	98.202	1.798	100
1_0.5M	0.5	29.157	0.843	30
1_1M	1	28.315	1.685	30
1_1.5M	1.5	27.472	2.528	30
1_2M	2	26.629	3.371	30
1_3M	3	24.944	5.056	30
1_4M	4	23.258	6.742	30
1_5M	5	21.573	8.427	30
1_6M	6	19.888	10.112	30
1_7M	7	18.202	11.798	30
1_8M	8	16.517	13.483	30
1_7M	9	14.831	15.169	30
2_0.01M	0.01	29.980	0.017	30
2_0.1M	0.1	29.830	0.169	30
3_1M	1	28.315	1.685	30
3_1.5M	1.5	27.475	2.530	30
3_2M	2	26.630	3.370	30
3_3M	3	24.940	5.060	30
3_4M	4	23.260	6.740	30
3_5M	5	21.570	8.430	30
3_6M	6	19.890	10.110	30
3_7M	7	18.200	11.800	30

S5 Potentiometric Titrations of H_2SO_4 Standard Solutions Against 0.1 mol·L^{-1} NaOH

An acid-base titration is required to determine the exact concentration of the prepared H_2SO_4 solution against 0.1 mol·L^{-1} NaOH. The experimental set is shown in Fig. S4. Before titration, the glass vessel, magnetic stirrer, dosing unit tube, and pH probe were rinsed thoroughly with deionised water. For H_2SO_4 solutions with concentrations exceeding 0.1 mol·L^{-1}, the samples were diluted with Milli-Q water to minimise acid error in pH measurements. The actual concentration was then determined using the dilution factor. To ensure the pH probe was fully submerged, at least 15 mL of Milli-Q water and H_2SO_4 solutions were added to the vessel. Titration was performed using the Tiamo software's titration method, with dosing parameters set to deliver 0.1 mol·L^{-1} NaOH. The amounts of H_2SO_4 solution and Milli-Q water used are listed in Table S4, and each titration was performed in triplicate. Results of each titration and average concentrations are reported in Table S5.

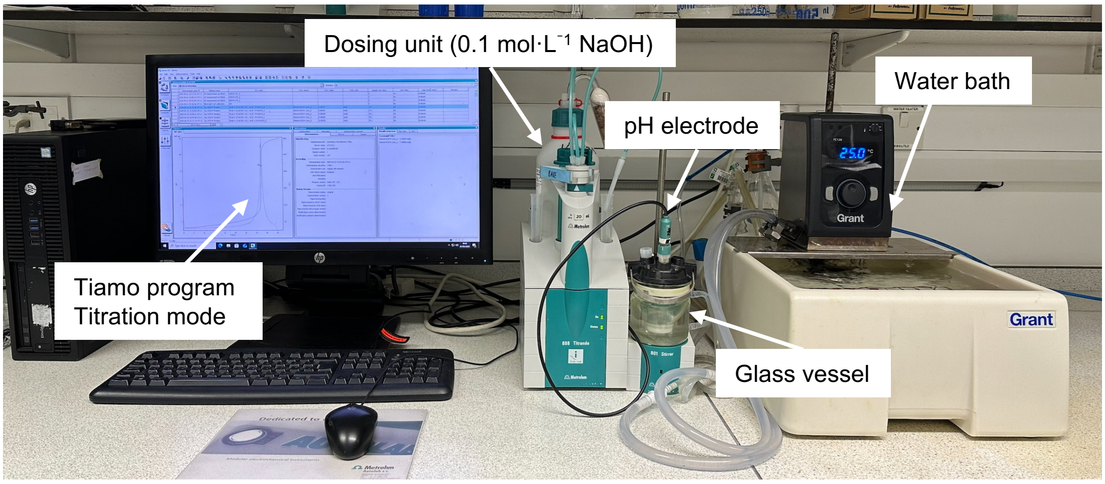

Figure S4 *Experimental setup used for acid-base potentiometric titration.*

Table S4 *Preparation of diluted H_2SO_4 solutions for titration against 0.1 mol·L^{-1} NaOH. Details of the volumes of Milli-Q water and H_2SO_4solutions used in the titration process to determine the concentration of H_2SO_4 are presented. This includes the precise amounts of each component required for accurate titration measurements.*

No.	Expected [H_2SO_4] mol·L^{-1}	H_2SO_4 solution added (mL)	Water added (mL)	Titration sample (mL)
1	0.0001	50	0	50
2	0.00032	50	0	50
3	0.001	50	0	50
4	0.0032	25	0	25
5	0.01	5.1	10	15.1
6	0.032	5.1	10	15.1
7	0.1	5.1	10	15.1
8	0.18	1.1	14	15.1
9	0.32	1.1	14	15.1
10	0.5	0.1	15	15.1
11	1	0.1	15	15.1
12	1.5	0.1	15	15.1
13	2	0.1	15	15.1
14	3	0.1	15	15.1
15	4	0.1	15	15.1
16	5	0.1	15	15.1
17	6	0.1	15	15.1
18	7	0.1	15	15.1
19	8	0.1	15	15.1
20	9	0.1	15	15.1

***Table S5** Titration results of H_2SO_4 concentrations ($mol \cdot L^{-1}$) against 0.1 $mol \cdot L^{-1}$ NaOH.*

ID	Titration 1	Titration 2	Titration 3	Average	SD
1_0.0001M	1.57E-04	1.59E-04	1.51E-04	1.56E-04	3.98E-06
1_0.00032M	4.04E-04	3.98E-04	3.96E-04	3.99E-04	4.50E-06
1_0.001M	1.18E-03	1.17E-03	1.19E-03	1.18E-03	1.13E-05
1_0.0032M	2.70E-03	3.36E-03	3.34E-03	3.13E-03	3.73E-04
1_0.01M	1.14E-02	1.15E-02	1.15E-02	1.15E-02	5.77E-05
1_0.032M	3.16E-02	3.11E-02	3.13E-02	3.13E-02	2.55E-04
1_0.1M	9.69E-02	9.54E-02	9.68E-02	9.64E-02	8.39E-04
1_0.18M	1.75E-01	1.77E-01	1.75E-01	1.76E-01	1.33E-03
1_0.32M	3.17E-01	3.19E-01	3.16E-01	3.18E-01	1.46E-03
1_0.5M	5.03E-01	5.03E-01	5.06E-01	5.04E-01	1.53E-03
1_1M	1.02E+00	1.02E+00	1.02E+00	1.02E+00	7.00E-04
1_1.5M	1.47E+00	1.47E+00	1.52E+00	1.49E+00	2.53E-02
1_2M	2.19E+00	2.21E+00	2.23E+00	2.21E+00	1.64E-02
1_3M	3.03E+00	3.08E+00	3.10E+00	3.07E+00	3.64E-02
1_4M	4.29E+00	4.34E+00	4.39E+00	4.34E+00	5.22E-02
1_5M	5.33E+00	5.42E+00	5.42E+00	5.39E+00	4.89E-02
1_6M	6.64E+00	6.65E+00	6.72E+00	6.67E+00	4.45E-02
1_7M	7.64E+00	7.70E+00	7.70E+00	7.68E+00	3.66E-02
1_8M	8.79E+00	8.74E+00	8.81E+00	8.78E+00	3.34E-02
1_9M	9.71E+00	9.62E+00	9.74E+00	9.69E+00	5.93E-02
2_0.01M	1.09E-02	-	-	1.09E-02	
2_0.1M	1.04E-01	-	-	1.04E-01	
3_1M	1.03E+00	1.03E+00	1.04E+00	1.03E+00	1.68E-03
3_1.5M	1.54E+00	1.53E+00	1.53E+00	1.53E+00	4.10E-03
3_2M	2.03E+00	2.03E+00	2.03E+00	2.03E+00	2.83E-03
3_3M	3.06E+00	3.07E+00	3.06E+00	3.06E+00	2.96E-03
3_4M	4.21E+00	4.14E+00	4.16E+00	4.17E+00	3.52E-02
3_5M	5.26E+00	5.26E+00	5.23E+00	5.25E+00	1.50E-02
3_6M	6.41E+00	6.38E+00	6.39E+00	6.39E+00	1.74E-02
3_7M	7.52E+00	7.64E+00	7.46E+00	7.54E+00	9.20E-02

S6 Proton Activity Calculations Using the Pitzer Model and the MacInnes Assumption in PHREEQC

The pH values of H_2SO_4 calibration solutions were calculated from proton activities using the PHREEQC program with the Pitzer model and the MacInnes assumption. The input code (Fig. S5) defines the solution blocks, including initial solution parameters (set at pH 7.0 with charge balance maintained), temperature, units in molarity, densities, and sulphate concentrations determined from titration experiments.

PHREEQC automatically converts between molality and molarity, adjusting densities to ensure consistency with its calculated values. For example, the output for solution 18 (Fig. S6) corresponds to a theoretical pH for around 7.68 $mol \cdot L^{-1}$ H_2SO_4. Key results include an adjusted density of 1.42 $g \cdot cm^{-3}$ and a sulphate molality of 11.32 $mol \cdot kg^{-1}$. At this concentration, the MacInnes activity reaches the program's upper limit of 1000, which occurs for H_2SO_4 > 5.7 $mol \cdot L^{-1}$. This MacInnes activity differs from proton activity coefficient, which must be determined using Eq. S1.

$$\gamma_{H^+} = \frac{10^{-pH}}{[H^+]} \qquad \text{Eq. S1}$$

Results of pH calculation for H_2SO_4 concentration < 8.5 $mol \cdot L^{-1}$ are shown in Table S6. For the ID_8M and ID_9M solutions, PHREEQC was unable to compute density due to the high concentration of H_2SO_4, which exceeds its operable limit of around 8.5 $mol \cdot L^{-1}$. To estimate theoretical pH, the concentration of H_2SO_4 must first be converted from molarity ($mol \cdot L^{-1}$) to molality ($mol \cdot kgw^{-1}$). While PHREEQC cannot perform this conversion automatically for these concentrated solutions, it can be calculated manually using known density values using density data from the CRC Handbook of Chemistry and Physics (Lide, 2000). For H_2SO_4 at 8.78 ± 0.03 $mol \cdot L^{-1}$, with an estimated density of 1.48 $g \cdot mL^{-1}$, the molality is 14.20 ± 0.13 $mol \cdot kgw^{-1}$. For H_2SO_4 at 9.69 ± 0.06 $mol \cdot L^{-1}$, with a density of 1.52 $g \cdot mL^{-1}$, the molality is 16.90 ± 0.27 $mol \cdot kgw^{-1}$ (Table S7). Details of each theoretical pH value with chemical speciation, and proton activity coefficient calculated from PHREEQC using Pitzer and MacInnes are shown in Table S8.

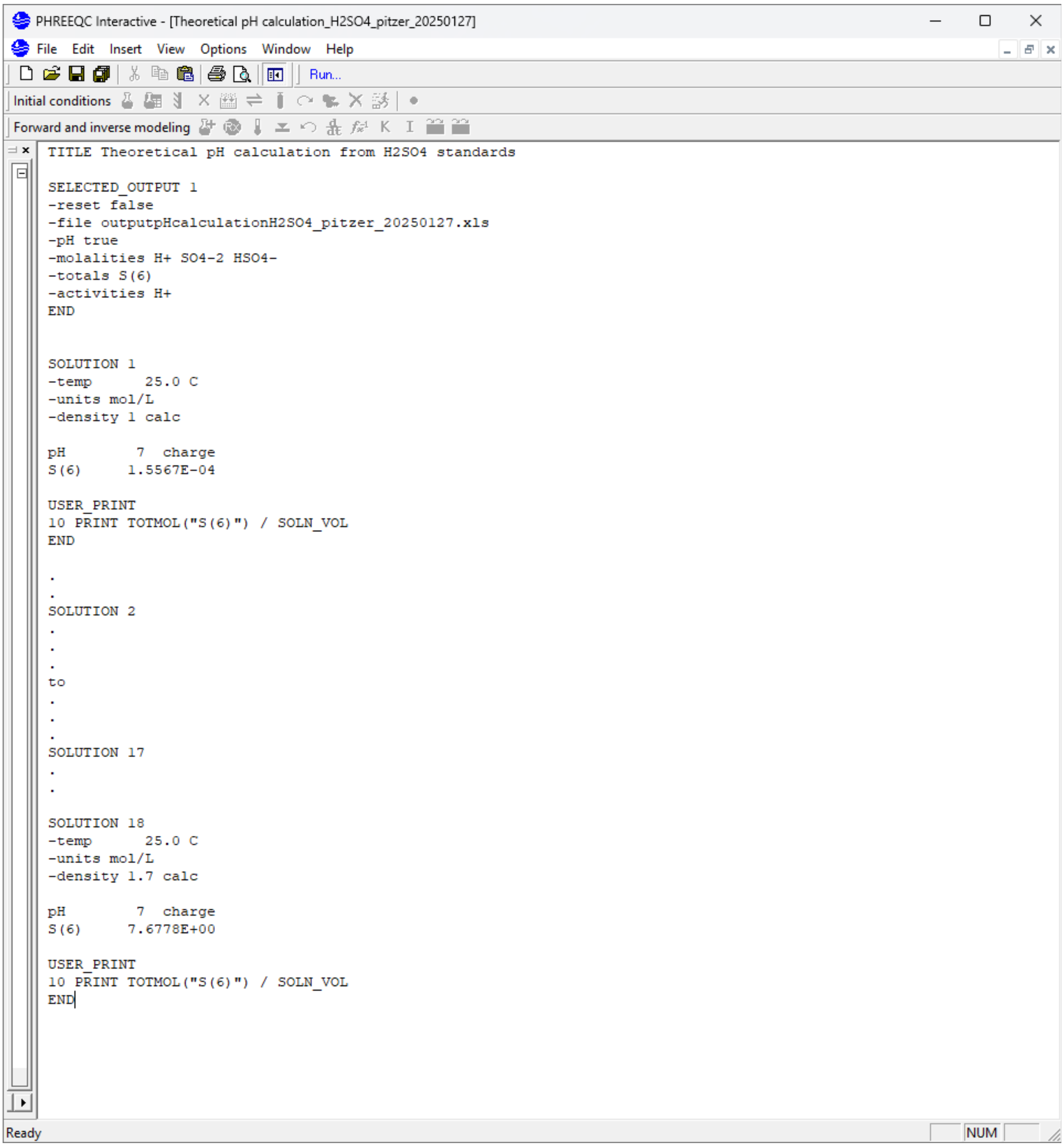


```
TITLE Theoretical pH calculation from H2SO4 standards

SELECTED_OUTPUT 1
-reset false
-file outputpHcalculationH2SO4_pitzer_20250127.xls
-pH true
-molalities H+ SO4-2 HSO4-
-totals S(6)
-activities H+
END


SOLUTION 1
-temp      25.0 C
-units mol/L
-density 1 calc

pH         7  charge
S(6)     1.5567E-04

USER_PRINT
10 PRINT TOTMOL("S(6)") / SOLN_VOL
END

.
.
SOLUTION 2
.
.
.
to
.
.
.
SOLUTION 17
.
.

SOLUTION 18
-temp      25.0 C
-units mol/L
-density 1.7 calc

pH         7  charge
S(6)     7.6778E+00

USER_PRINT
10 PRINT TOTMOL("S(6)") / SOLN_VOL
END
```

Figure S5 *PHREEQC input for calculating the theoretical pH of H_2SO_4 standard solutions using the Pitzer and MacInnes model. SOLUTION blocks define solution parameters, including temperature (25°C), unit in molarity, density of H_2SO_4, and sulphate concentrations for various H_2SO_4 standards.*

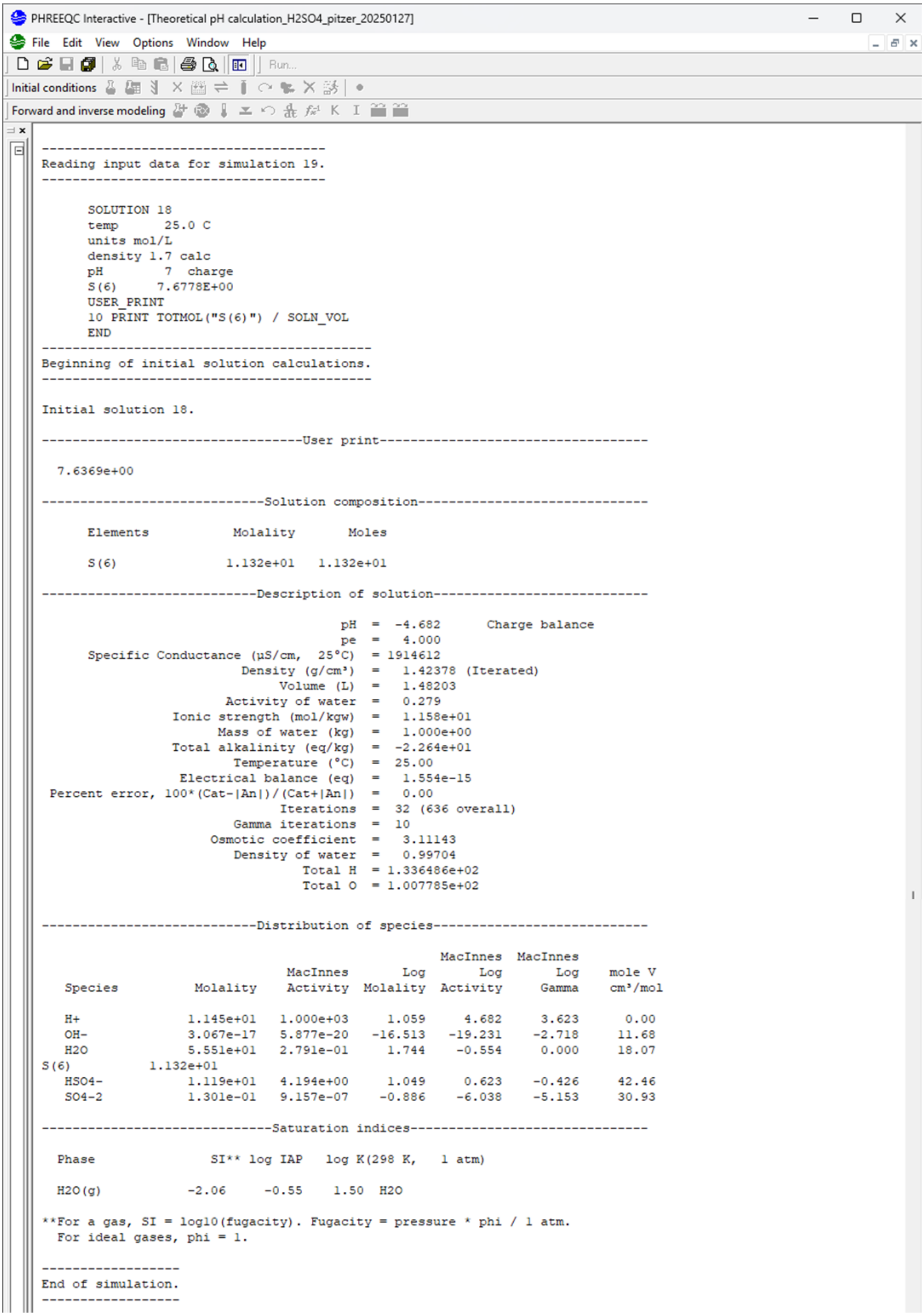

PHREEQC Interactive - [Theoretical pH calculation_H2SO4_pitzer_20250127]

File Edit View Options Window Help

Initial conditions

Forward and inverse modeling

```
------------------------------------
Reading input data for simulation 19.
------------------------------------

      SOLUTION 18
      temp      25.0 C
      units mol/L
      density 1.7 calc
      pH         7  charge
      S(6)      7.6778E+00
      USER_PRINT
      10 PRINT TOTMOL("S(6)") / SOLN_VOL
      END
-------------------------------------------
Beginning of initial solution calculations.
-------------------------------------------

Initial solution 18.

----------------------------------User print----------------------------------

  7.6369e+00

-----------------------------Solution composition------------------------------

      Elements           Molality       Moles

      S(6)              1.132e+01   1.132e+01

----------------------------Description of solution----------------------------

                                       pH  =  -4.682      Charge balance
                                       pe  =   4.000
      Specific Conductance (µS/cm,  25°C)  = 1914612
                          Density (g/cm³)  =   1.42378 (Iterated)
                               Volume (L)  =   1.48203
                        Activity of water  =   0.279
                 Ionic strength (mol/kgw)  =   1.158e+01
                       Mass of water (kg)  =   1.000e+00
                 Total alkalinity (eq/kg)  =  -2.264e+01
                         Temperature (°C)  =  25.00
                   Electrical balance (eq) =   1.554e-15
 Percent error, 100*(Cat-|An|)/(Cat+|An|)  =   0.00
                               Iterations  =  32 (636 overall)
                         Gamma iterations  =  10
                      Osmotic coefficient  =   3.11143
                         Density of water  =   0.99704
                                  Total H  = 1.336486e+02
                                  Total O  = 1.007785e+02

----------------------------Distribution of species----------------------------

                                                    MacInnes  MacInnes
                                MacInnes      Log       Log       Log    mole V
   Species          Molality    Activity  Molality  Activity     Gamma   cm³/mol

   H+              1.145e+01   1.000e+03     1.059     4.682     3.623      0.00
   OH-             3.067e-17   5.877e-20   -16.513   -19.231    -2.718     11.68
   H2O             5.551e+01   2.791e-01     1.744    -0.554     0.000     18.07
S(6)          1.132e+01
   HSO4-           1.119e+01   4.194e+00     1.049     0.623    -0.426     42.46
   SO4-2           1.301e-01   9.157e-07    -0.886    -6.038    -5.153     30.93

------------------------------Saturation indices-------------------------------

  Phase               SI** log IAP   log K(298 K,   1 atm)

  H2O(g)           -2.06    -0.55     1.50  H2O

**For a gas, SI = log10(fugacity). Fugacity = pressure * phi / 1 atm.
  For ideal gases, phi = 1.

------------------
End of simulation.
------------------
```

***Figure S6** An example of PHREEQC output for solution with 7.6778 mol·L^{-1} H_2SO_4.*

Table S6 *Theoretical pH± error calculated from PHREEQC using Pitzer and MacInnes.*

ID	Average H_2SO_4 mol·L^{-1}	±SD mol·L^{-1}	pH H_2SO_4 standards	-pH Error lower bound	+pH Error upper bound
1_0.0001M	1.56E-04	3.98E-06	3.52	0.01	0.01
1_0.00032M	3.99E-04	4.50E-06	3.13	0.01	0.00
1_0.001M	1.18E-03	1.13E-05	2.69	0.00	0.00
1_0.0032M	3.13E-03	3.73E-04	2.31	0.04	0.05
1_0.01M	1.15E-02	5.77E-05	1.82	0.00	0.00
1_0.032M	3.13E-02	2.55E-04	1.45	0.00	0.00
1_0.1M	9.64E-02	8.39E-04	1.02	0.00	0.00
1_0.18M	1.76E-01	1.33E-03	0.77	0.00	0.00
1_0.32M	3.18E-01	1.46E-03	0.51	0.00	0.00
1_0.5M	5.04E-01	1.53E-03	0.28	0.00	0.00
1_1M	1.02E+00	7.00E-04	-0.13	0.00	0.00
1_1.5M	1.49E+00	2.53E-02	-0.43	0.01	0.02
1_2M	2.21E+00	1.64E-02	-0.84	0.01	0.01
1_3M	3.07E+00	3.64E-02	-1.34	0.02	0.02
1_4M	4.34E+00	5.22E-02	-2.11	0.03	0.03
1_5M	5.39E+00	4.89E-02	-2.81	0.04	0.03
1_6M	6.67E+00	4.45E-02	-3.78	0.03	0.04
1_7M	7.68E+00	3.66E-02	-4.68	0.03	0.04
2_0.01M	1.09E-02	0.00E+00	1.84	0.00	0.00
2_0.1M	1.04E-01	0.00E+00	0.99	0.00	0.00
3_1M	1.03E+00	1.68E-03	-0.14	0.01	0.00
3_1.5M	1.53E+00	4.10E-03	-0.45	0.01	0.00
3_2M	2.03E+00	2.83E-03	-0.74	0.00	0.00
3_3M	3.06E+00	2.96E-03	-1.33	0.01	0.00
3_4M	4.17E+00	3.52E-02	-2.00	0.02	0.03
3_5M	5.25E+00	1.50E-02	-2.71	0.01	0.01
3_6M	6.39E+00	1.74E-02	-3.55	0.02	0.02
3_7M	7.54E+00	9.20E-02	-4.55	0.09	0.09

Table S7 *Theoretical pH ± error calculated from PHREEQC using Pitzer and MacInnes for H_2SO_4 higher than 8.5 mol·L^{-1}.*

ID	H_2SO_4 mol·kgw^{-1}	±SD mol·kgw^{-1}	pH H_2SO_4 standards	-pH Error lower bound	+pH Error upper bound
1_8M	14.20E+00	1.29E-02	-6.02	0.06	0.06
1_9M	16.90E+00	2.72E-02	-7.39	0.15	0.14

Table S8 *Theoretical pH, chemical speciation, and proton activity coefficient calculated from PHREEQC using Pitzer and MacInnes.*

Average H_2SO_4 mol·L-1	pH H_2SO_4 standards	S(6)	m_H$^+$	m_SO$_4^{-2}$	m_HSO$_4^-$	H^+ activity coefficient
1.56E-04	3.52	1.56E-04	3.09E-04	1.52E-04	4.06E-06	1.0
3.99E-04	3.13	4.00E-04	7.77E-04	3.76E-04	2.38E-05	1.0
1.18E-03	2.69	1.18E-03	2.20E-03	1.02E-03	1.65E-04	0.9
3.13E-03	2.31	3.14E-03	5.46E-03	2.32E-03	8.18E-04	0.9
1.15E-02	1.82	1.15E-02	1.77E-02	6.17E-03	5.37E-03	0.9
3.13E-02	1.45	3.14E-02	4.36E-02	1.22E-02	1.92E-02	0.8
9.64E-02	1.02	9.70E-02	1.24E-01	2.68E-02	7.02E-02	0.8
1.76E-01	0.77	1.78E-01	2.20E-01	4.27E-02	1.35E-01	0.8
3.18E-01	0.51	3.23E-01	3.95E-01	7.21E-02	2.50E-01	0.8
5.04E-01	0.28	5.15E-01	6.29E-01	1.14E-01	4.00E-01	0.8
1.02E+00	-0.13	1.06E+00	1.31E+00	2.52E-01	8.09E-01	1.0
1.49E+00	-0.43	1.58E+00	1.97E+00	3.93E-01	1.19E+00	1.4
2.21E+00	-0.84	2.41E+00	3.01E+00	5.94E-01	1.82E+00	2.3
3.07E+00	-1.34	3.48E+00	4.23E+00	7.52E-01	2.73E+00	5.1
4.34E+00	-2.11	5.25E+00	6.01E+00	7.61E-01	4.49E+00	21.4
5.39E+00	-2.81	6.91E+00	7.51E+00	6.06E-01	6.30E+00	85.9
6.67E+00	-3.78	9.23E+00	9.55E+00	3.25E-01	8.90E+00	629.0
7.68E+00	-4.68	1.13E+01	1.15E+01	1.30E-01	1.12E+01	4218.2
1.09E-02	1.84	1.09E-02	1.69E-02	5.94E-03	5.00E-03	0.9
1.04E-01	0.99	1.05E-01	1.33E-01	2.83E-02	7.64E-02	0.8
1.03E+00	-0.14	1.07E+00	1.33E+00	2.55E-01	8.17E-01	1.0
1.53E+00	-0.45	1.62E+00	2.03E+00	4.05E-01	1.22E+00	1.4
2.03E+00	-0.74	2.20E+00	2.75E+00	5.48E-01	1.65E+00	2.0
3.06E+00	-1.33	3.47E+00	4.22E+00	7.51E-01	2.72E+00	5.1
4.17E+00	-2.00	5.00E+00	5.77E+00	7.74E-01	4.22E+00	17.4
5.25E+00	-2.71	6.67E+00	7.31E+00	6.33E-01	6.04E+00	70.6
6.39E+00	-3.55	8.69E+00	9.08E+00	3.88E-01	8.30E+00	393.4
7.54E+00	-4.55	1.10E+01	1.12E+01	1.52E-01	1.09E+01	3167.0

S7 EMF Measurements of pH standards (H_2SO_4 solutions and Hanna pH buffers)

Fig. S7 shows the experimental setup used for negative pH calibration. For each measurement, the electrode was removed from its storage solution (Metrohm 6.2323.000 or Hanna HI-70300L), rinsed with Milli-Q water, and gently blotted dry. The electrode was then immersed in the sample, ensuring both the glass bulb and reference junction were fully submerged. Readings were taken once the EMF stabilised (dU/dt < 0.5 mV·min^{-1}). After recording, the electrode was rinsed and either reinserted into the next solution or returned to storage.

Although experiments were conducted under the same conditions of 25 °C, slight variations were recorded due to thermal gradients and equilibration times. For example, the negative pH calibration of the Metrohm Primatrode electrode H was measured at 24.0 ± 0.3 °C, while the standard linear pH calibration was obtained at 24.4 ± 0.1 °C. Results of EMF-pH data for Hanna pH buffer and H_2SO_4 standards for different electrodes and different temperature are shown in Table S9-S17.

The Orion Ross Ultra pH/ATC Triode electrode was tested for negative pH calibration but only a single experiment could be conducted, as the electrode showed a loss of sensitivity after the EMF-pH measurements at pH -0.74 and -1.33 (Table S16 and Fig. S10). At these two points, the recorded EMF values were substantially lower than expected, indicating that the electrode response was no longer reliable under such conditions. This highlight suggests that electrode performance in the negative pH range can vary between models, with each electrode type having a practical operating range for accurate measurements.

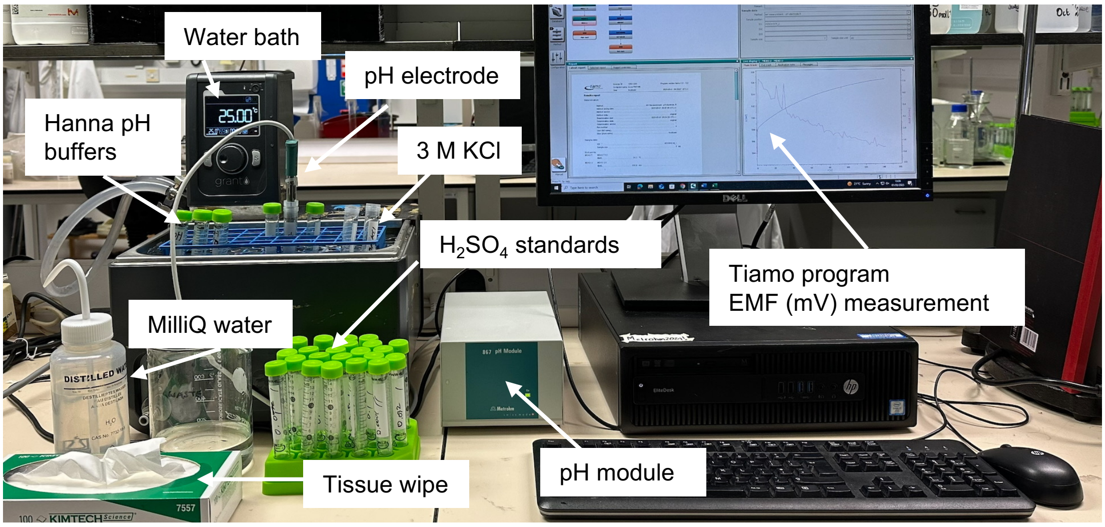

Figure S7 *Experimental setup used for EMF-pH measurements of pH standards.*

***Table S9** EMF measurements of different pH electrodes for the standard 3-point calibration using Hanna pH buffers at 25 °C.*

Metrohm Primatrode H			Metrohm Unitrode N		
pH	EMF (mV)	T (°C)	pH	EMF (mV)	T (°C)
4.01	182.1	24.5	4.01	169.8	24.6
7.01	6.1	24.4	7.01	-3.8	24.5
10.02	-171	24.3	10.01	-179.2	24.7
**Orion Ross-Sure Flow**			**Orion Ross- Ultra pH/ATC Triode**		
pH	EMF (mV)	T (°C)	pH	EMF (mV)	T (°C)
4.01	-235.80	23.8	4.01	147	24.4
7.01	-69.00	23.4	7.01	-28.1	24.2
10.01	112.1	23.6	10.01	-199.8	24.3

***Table S10** Summary statistics of the linear least-squares regression for the standard pH calibration of different electrodes, using the general model: EMF(pH) = E_0 + (s × pH), where E_0 is the intercept and s is the slope. For Orion Ross electrode, data is from the data point 4.03 & 3.00 in Table S12.*

LINEST Function (with standard error, se)							
Statistic	Value	Statistic	Value	Statistic	Value	Statistic	Value
1. Metrohm Unitrode N				2. Metrohm Primatrode H			
s	-58.13	E_0	403.2	s	-58.79	E_0	418.0
se s	0.18	se E_0	1.4	se s	0.12	se E_0	0.9
R^2	1	se EMF	0.8	R^2	1.00	se EMF	0.5
F	99510	d_f	1	F	252301	d_f	1
ss reg	60900	Ss resid	0.61	ss reg	62340	Ss resid	0.25
3. Orion Ross Sure-Flow				4. Orion Ross Ultra pH/ATC Triode			
s	-57.98	E_0	341.7	s	-57.80	E_0	377.6
se s	1.38	se E_0	10.2	se s	0.33	se E_0	2.4
R^2	1.00	se EMF	5.8	R^2	1.00	se EMF	1.4
F	1776	d_f	1	F	31212	d_f	1
ss reg	60517	Ss resid	34.08	ss reg	60135	Ss resid	1.93
5. Orion Ross							
s	-60.49	E_0	401.6				
se s	0.00	se E_0	0				
R^2	1.00	se EMF	0				
F	Na	d_f	0				
ss reg	1941	Ss resid	0				

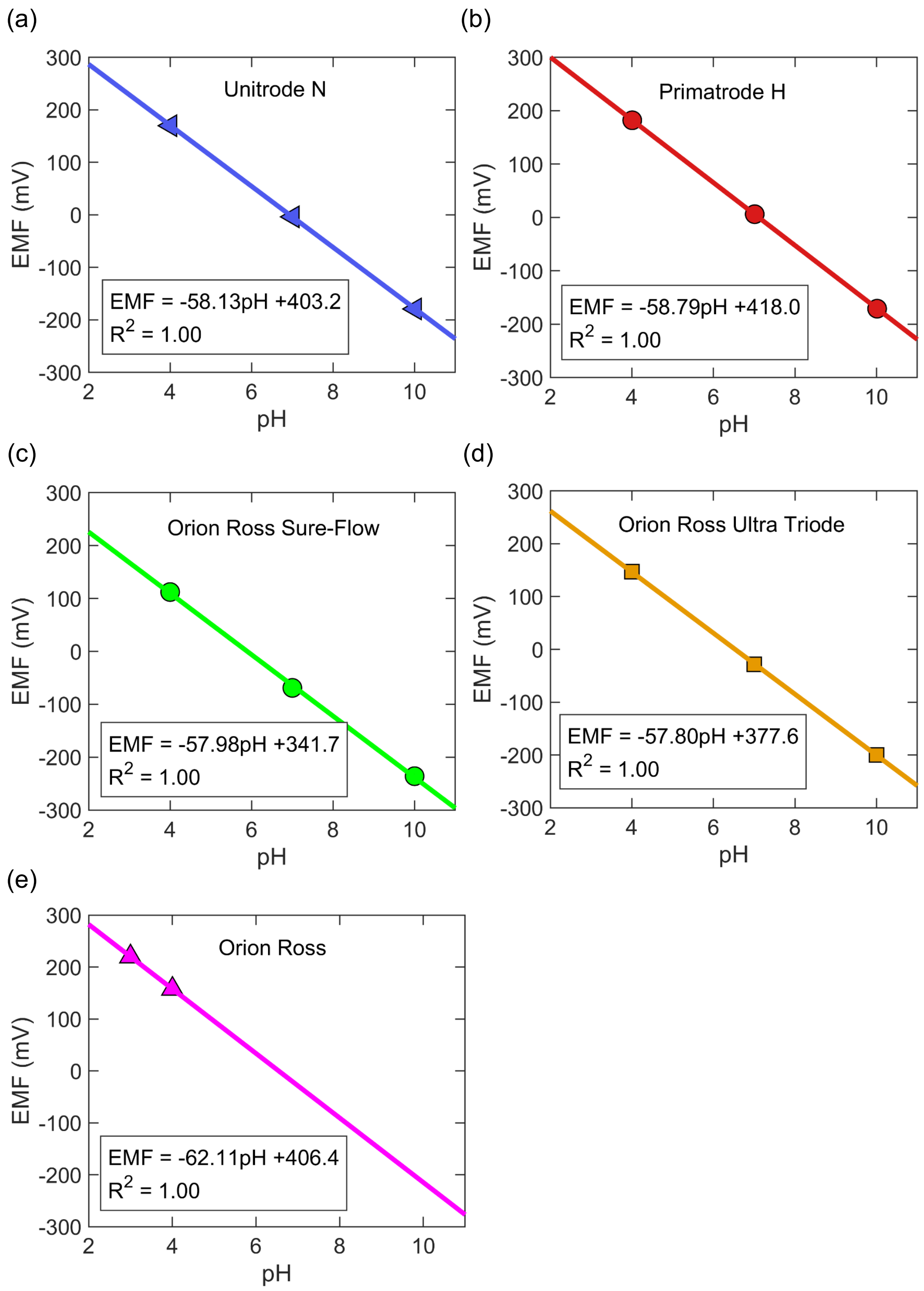

Figure S8 *The linear least-squares regression for the standard pH calibration of different electrodes.*

Table S11 *EMF measurements at 25°C of Orion Ross electrode for each H_2SO_4 standards (data extracted from Nordstrom et al., 2000) and estimated correction factors.*

pH	EMF Measurement (mV)	Estimated EMF Nernst (mV)	Estimated CF (mV)
4.03	157.8	157.8	0.0
3.00	220.1	220.1	0.0
2.00	276.6	280.2	3.6
1.02	336.7	339.3	2.6
0.86	346.5	349.1	2.6
0.14	385.5	392.8	7.3
-0.37	406.1	423.4	17.3
-0.77	421.4	447.5	26.1
-1.04	430.8	463.9	33.1
-1.38	439.7	484.6	44.9
-1.73	447.4	505.4	58.0
-2.16	455.4	531.6	76.2
-3.09	470.7	587.4	116.7

Table S12 *EMF measurements of Orion Ross electrode at different temperature (25, 35, 41, & 45 °C) for each H_2SO_4 standards (data extracted from Nordstrom et al., 2000).*

25 °C		35 °C		41 °C		45 °C	
pH	EMF (mV)	pH	EMF (mV)	pH	EMF (mV)	pH	EMF (mV)
4.03	157.8	2.02	286.5	0.15	405.7	0.19	412.0
3.00	220.1	0.99	334.4	-0.30	427.2	-0.66	448.3
2.00	276.6	0.15	396.7	-0.68	441.1	-1.98	487.3
1.02	336.7	-0.35	417.8	-2.00	481.0	-2.90	502.5
0.86	346.5	-0.73	431.7	-2.96	496.2	-3.88	514.1
0.14	385.5	-1.00	441.1	-3.88	504.3		
-0.37	406.1	-1.33	450.0				
-0.77	421.4	-2.05	468.0				
-1.04	430.8	-3.01	487.3				
-1.38	439.7	-3.99	499.8				
-1.73	447.4						
-2.16	455.4						
-3.09	470.7						
-4.06	483.7						

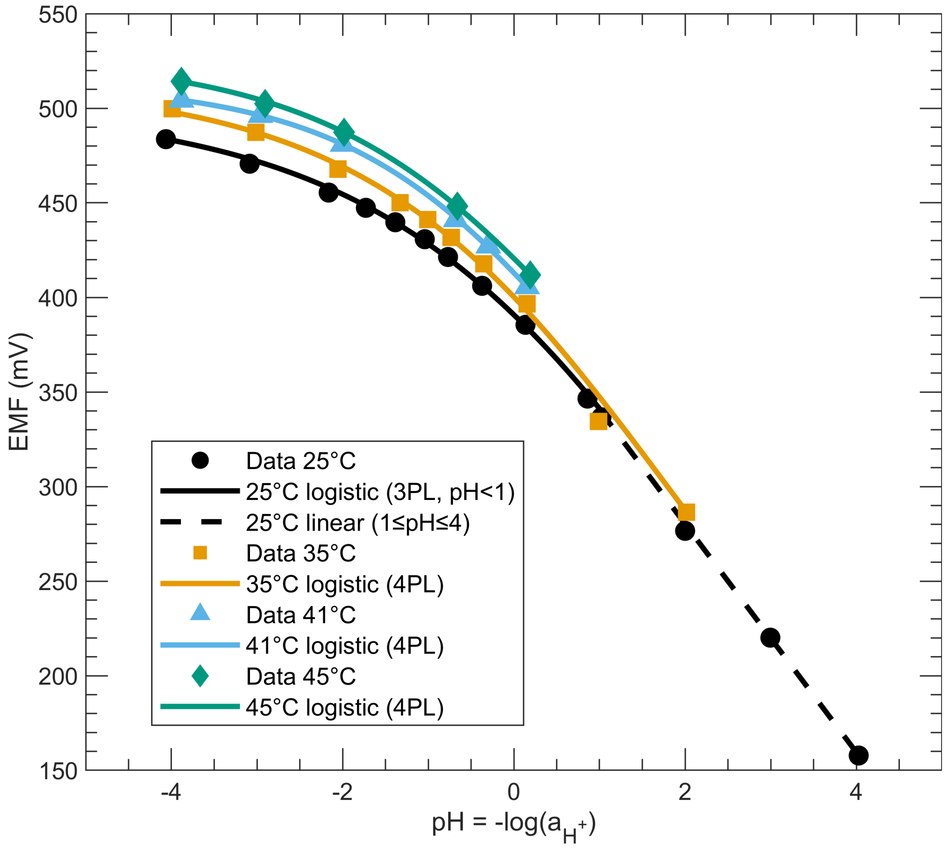

Figure S9 *Negative-pH calibrations for the Orion Ross electrode at 25, 35, 41, and 45 °C. Data were from Nordstrom et al. (2000) and are summarised in Table S12. At 25 ° C, the non-linear electrode response calibration follows the method used in this study: data points with pH ≤ 4 were fit with a 3-parameter logistic function with the lower asymptote fixed at 0. This negative pH calibration model is then used only for pH < 1; for pH > 1 the standard calibration curve is applied. At 35, 41, and 45 °C, a 4-parameter logistic function (including a free lower asymptote) was used to better predictions in the negative-pH region.*

Table S13 *EMF (mV) and temperature (T, °C) measurements of Primatrode H electrode at 25 °C for each pH standards and estimated correction factors.*

pH of H_2SO_4	Exp 1		Exp 2		Exp 3		EMF(mV)		T (°C)		CF
solution standard	EMF 1	T 1	EMF 2	T 2	EMF 3	T 3	Average	±SD	Average	±SD	(mV)
3.52	209.1	23.8	212.3	23.2	211.4	24.2	211.9	1.7	23.7	0.5	-0.9
3.13	236.3	23.6	234.4	23.8	233.6	23.6	234.0	1.4	23.7	0.1	0.2
3.00	241.6	24.1	241.0	24.5	241.4	24.8	241.4	0.3	24.5	0.4	0.2
2.69	260.4	23.3	260.8	23.8	260.7	23.7	260.8	0.2	23.6	0.3	-0.7
2.31	287.5	23.6	285.0	23.6	285.6	24.1	286.0	1.3	23.8	0.3	-3.6
1.82	314.3	24.1	314.6	24.2	313.6	24.1	314.2	0.5	24.1	0.1	-3.3
1.68	319.8	24.4	319.7	24.8	320.5	25.0	320.0	0.4	24.7	0.3	-0.8
1.45	336.0	24.1	335.5	24.0	334.9	23.8	335.5	0.6	24.0	0.2	-2.8
1.02	359.8	24.1	358.5	23.5	361.5	24.3	359.9	1.5	24.0	0.4	-1.9
0.77	369.4	23.9	371.7	23.8	373.2	24.2	371.4	1.9	24.0	0.2	1.1
0.51	383.1	24.3	385.0	23.9	384.0	23.4	384.0	1.0	23.9	0.5	4.0
0.28	396.3	24.4	397.7	24.3	393.5	23.0	395.8	2.1	23.9	0.8	5.5
-0.14	412.0	24.0	414.3	24.3	411.6	22.8	412.6	1.5	23.7	0.8	13.3
-0.43	423.1	22.9	421.9	24.5	425.0	24.3	423.3	1.6	23.9	0.9	19.8
-0.84	433.5	23.6	438.8	24.4	435.7	24.2	436.0	2.7	24.1	0.4	31.6
-1.34	446.2	23.9	450	23.8	445.6	24.3	447.3	2.4	24	0.3	49.3
-2.11	455.7	23.2	467.6	23.8	451.1	23	458.1	8.5	23.3	0.4	83.8
-2.81	477.7	24.5	478.2	23.3	477.4	24.4	477.8	0.4	24.1	0.7	105.4
-3.78	486.7	23.8	495.4	23.8	490.8	24.4	491	4.4	24	0.3	149.1
-4.68	499.0	23.9	502.8	23.4	501.2	24.4	501	1.9	23.9	0.5	192.3
-6.02	510.5	23.7	514.0	23.0	511.7	24.4	512.1	1.8	23.7	0.7	260.1
-7.39	518.7	23.0	527.5	24.4	520.1	24.3	522.1	4.7	23.9	0.8	330.4

Table S14 *EMF (mV) and temperature (°C) measurements of Unitrode N electrode at 25 °C for each pH standards and estimated correction factors.*

pH of H_2SO_4	Exp 1		Exp 2		Exp 3		EMF(mV)		T (°C)		CF
solution standard	EMF 1	T 1	EMF 2	T 2	EMF 3	T 3	Average	±SD	Average	±SD	(mV)
3.52	193.5	24.7	190.7	25.1	195.7	24.7	193.3	2.5	24.8	0.2	5.2
3.00	239.1	24.7	230.4	24.9	230.5	25.1	233.3	5.0	24.9	0.2	-4.6
2.68	252.0	25.0	249.5	24.7	249.5	24.7	250.3	1.4	24.8	0.2	-2.7
1.81	300.8	24.7	299.9	24.7	300.3	24.7	300.3	0.5	24.7	0.0	-2.5
1.68	310.3	24.6	310.3	25.0	309.8	25.1	310.1	0.3	24.9	0.3	-4.6
0.96	348.9	24.5	348.9	24.8	349.9	24.7	349.2	0.6	24.7	0.2	-1.8
0.48	372.6	24.8	373.3	24.8	373.5	24.6	373.1	0.5	24.7	0.1	2.1
-0.14	401.7	24.8	402.3	24.8	402.3	24.8	402.1	0.3	24.8	0.0	9.4
-0.40	412.9	24.8	413.6	24.8	413.3	24.8	413.3	0.4	24.8	0.0	16.3
-0.74	421.9	24.9	422.1	24.8	422.2	24.9	422.1	0.2	24.9	0.1	24.1
-1.33	437.8	24.9	438.4	24.9	438.4	24.9	438.2	0.3	24.9	0.0	42.4
-2.00	452.0	24.9	452.4	25.0	452.3	25.0	452.2	0.2	25.0	0.1	67.2
-2.72	465.2	24.9	466.3	25.0	466.2	25.0	465.9	0.6	25.0	0.1	95.3
-3.55	479.1	25.0	479.7	25.0	479.9	25.0	479.6	0.4	25.0	0.0	130.2
-4.55	492.1	25.0	490.4	25.0	493.4	25.0	492.0	1.5	25.0	0.0	175.5
-6.02	518.9	25.0	517.1	24.7	507.1	24.8	514.4	6.4	24.8	0.2	238.9
-7.39	518.4	24.8	524.2	25.1	507.9	24.8	516.8	8.3	24.9	0.2	315.9

Table S15 *EMF (mV) and temperature (°C) measurements of Orion Ross-Sure Flow electrode at 25 °C for each pH standards and estimated correction factors.*

pH of H_2SO_4	Exp 1		Exp 2		Exp 3		EMF(mV)		T (°C)		CF
solution standard	EMF 1	T 1	EMF 2	T 2	EMF 3	T 3	Average	±SD	Average	±SD	(mV)
3.52	132.9	23.8	140.2	24.4	134.4	24.5	135.8	3.9	24.2	0.4	1.6
3.00	168.5	24.4	168.9	24.4	171.4	24.3	169.6	1.6	24.4	0.1	-1.9
2.69	186.4	23.7	190.6	24.4	191.1	24.5	189.4	2.6	24.2	0.4	-3.5
1.81	249.4	23.7	243.0	24.4	241.5	24.4	244.6	4.2	24.2	0.4	-8.0
1.68	249.9	24.3	250.5	24.4	250.4	24.4	250.3	0.3	24.4	0.1	-6.0
0.96	289.1	23.6	285.5	24.6	288.1	24.6	287.6	1.9	24.3	0.6	-1.5
0.48	314.6	23.7	313.6	24.5	313.0	24.5	313.7	0.8	24.2	0.5	0.0
-0.14	342.8	23.8	342.9	24.5	340.3	24.5	342.0	1.5	24.3	0.4	8.0
-0.45	355.2	24.0	353.9	24.4	353.6	24.5	354.2	0.9	24.3	0.3	13.8
-0.74	356.9	23.8	362.7	24.2	363.5	24.4	361.0	3.6	24.1	0.3	23.5
-1.33	373.5	23.9	379.3	24.5	376.1	24.5	376.3	2.9	24.3	0.3	42.6
-2.00	384.2	23.8	379.5	24.5	389.7	24.5	384.5	5.1	24.3	0.4	73.2
-2.72	406.1	24.0	398.0	24.4	394.5	24.3	399.5	6.0	24.2	0.2	99.8
-3.55	422.7	24.0	420.1	24.4	421.0	24.4	421.3	1.3	24.3	0.2	126.5
-4.55	431.8	23.8	434.0	24.2	431.3	24.4	432.4	1.4	24.1	0.3	173.0
-6.02	448.4	23.9	447.8	24.4	440.0	24.4	445.4	4.7	24.2	0.3	245.5

Table S16 *EMF (mV) and temperature (°C) measurements of Orion Ross Ultra pH/ATC Triode electrode at 25 °C for each pH standards and estimated correction factors. This experiment was performed only once, as the electrode lost sensitivity following the EMF-pH measurements at pH -0.74 and -1.33*

pH of H_2SO_4 solution standard	EMF (mV)	T (°C)	CF
3.52	171.6	23.6	2.4
3.00	206.8	23.8	-2.6
2.69	223.7	23.4	-1.3
1.81	273.9	23.2	-1.0
0.96	320.0	23.2	2.2
0.48	341.3	23.6	8.5
-0.14	368.6	23.6	17.4
-0.45	380.7	23.6	23.2
-0.74	316.0	23.6	104.4
-1.33	328.4	23.6	126.3

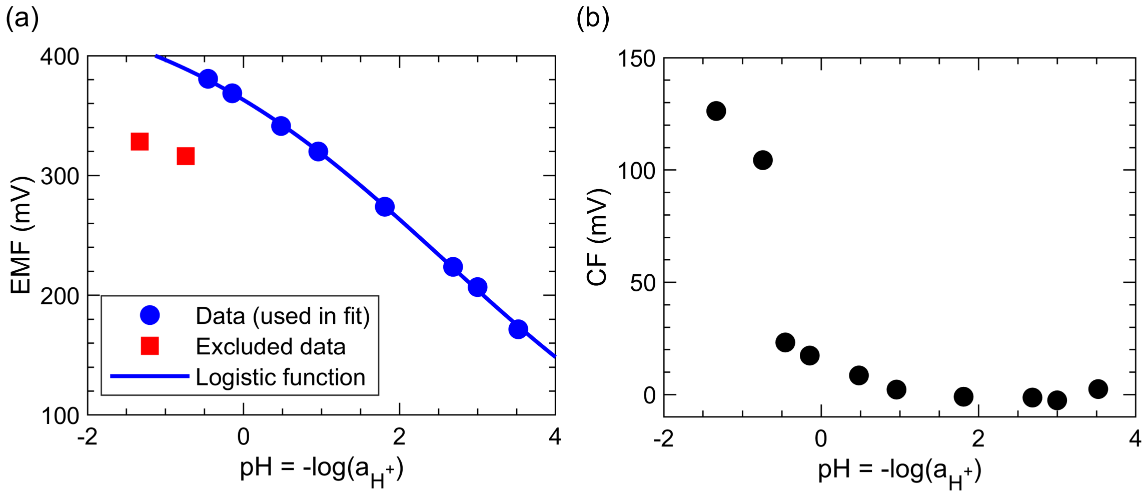

Figure S10 EMF-pH and CF data of Orion Ross Ultra pH/ATC Triode electrode. (a) Negative pH calibration curve of Orion Ross Ultra pH/ATC Triode electrode excluding the pH -0.74 and -1.33 datapoints because the electrode lost its sensitivity after these two extreme acid solutions. (b) Estimated CF value of the Orion Ross Ultra pH/ATC Triode electrode.

Table S17 *EMF (mV) and temperature (T, °C) measurements for the standard 3-point calibration of the Metrohm Primatrode H electrode across different temperatures. The corresponding pH values were recorded with 3 decimal places, as reported by the Tiamo software. This additional precision results from the software's internal calculations and temperature correction of the pH buffers, although the practical measurement uncertainty of pH determinations is typically limited to 1 or 2 decimal places.*

Temperature setpoint (°C)	Exp 1			Exp 2			Exp 3		
	pH	mV	T °C	pH	mV	T °C	pH	mV	T °C
5.0	4.000	178.6	6.9	4.000	179.1	6.9	4.000	177.6	6.9
	7.089	9.6	6.9	7.089	9.0	6.8	7.091	5.4	6.6
	10.227	-166.5	6.9	10.227	-165.8	6.7	10.226	-164.4	6.7
15.0	4.000	179.7	15.9	4.000	179.8	15.9	4.000	179.7	15.9
	7.046	6.8	15.9	7.047	6.1	15.9	7.047	7.0	15.9
	10.110	-165.8	15.8	10.110	-167.0	15.9	10.110	-167.1	15.9
25.0	4.009	182.1	24.3	4.009	181.5	24.4	4.009	182.0	24.3
	7.013	6.1	24.4	7.012	5.9	24.4	7.013	6.0	24.4
	10.015	-171.0	24.5	10.015	-170.9	24.5	10.015	-170.7	24.5
35.0	4.023	184.3	31.4	4.023	184.3	31.4	4.023	184.3	31.5
	6.997	5.3	31.4	6.997	5.4	31.5	6.997	5.4	31.6
	9.948	-173.2	31.5	9.948	-172.9	31.6	9.947	-172.8	31.6
40.0	4.038	185.5	38.8	4.038	187.0	38.8	4.038	185.3	38.8
	6.982	3.3	38.8	6.982	4.5	38.9	6.982	4.4	39.0
	9.889	-176.1	38.9	9.888	-175.8	39.0	9.888	-176.1	39.0
50.0	4.056	186.3	47.9	4.057	189.1	48.5	4.056	189.4	48.2
	6.980	1.3	48.0	6.980	4.1	48.6	6.980	3.3	48.4
	9.832	-180.9	48.0	9.829	-177.1	48.6	9.829	-176.8	48.6
60.0	4.085	189.5	57.6	4.085	189.3	57.5	4.085	188.0	57.7
	6.980	0.6	57.8	6.980	-0.2	57.7	6.980	0.1	58.1
	9.779	-182.2	57.8	9.778	-181.2	58.0	9.778	-182.0	58.0

Table S18 *Results of E_0 and slope from the standard 3-point calibration of the Metrohm Primatrode H electrode across different temperatures.*

Temperature setpoint (°C)	Temp (NTC, °C)	E_0 at T	Slop	%Slop
5	6.6	401.0	-55.42	99.7
	6.7	401.0	-55.39	99.8
	6.7	396.5	-54.93	98.9
15	15.8	405.7	-56.55	98.6
	15.9	406.6	-56.76	99.0
	15.9	406.8	-56.76	99.0
25	24.5	418.0	-58.79	99.5
	24.5	416.9	-58.67	99.4
	24.5	417.6	-58.72	99.4
35	31.5	427.2	-60.34	99.8
	31.6	427.0	-60.29	99.7
	31.6	426.9	-60.28	99.7
40	38.9	435.0	-61.80	99.8
	39.0	437.5	-62.02	100.1
	39.0	435.1	-61.78	99.7
50	48.0	444.4	-63.57	99.8
	48.6	446.6	-63.44	99.4
	48.6	446.5	-63.43	99.4
60	57.8	456.2	-65.28	99.4
	58.0	454.8	-65.08	99.1
	58.0	453.6	-64.99	98.9

***Table S19** EMF (mV) and temperature (T, °C) measurements of Metrohm Primatrode H electrode at 5 °C.*

pH of H_2SO_4 solution standard	Exp 1		Exp 2		Exp 3		EMF(mV)		T (°C)	
	EMF 1	T 1	EMF 2	T 2	EMF 3	T 3	Average	±SD	Average	±SD
4.00	178.6	6.9	n.d.	n.d.	n.d.	n.d.	178.6	n.d.	6.9	n.d.
3.52	202.3	7.0	203.1	7.7	203.8	7.7	203.1	0.8	7.5	0.4
3.05	229.5	7.6	n.d.	n.d.	n.d.	n.d.	229.5	n.d.	7.6	n.d.
3.12	232.6	6.6	228.1	7.6	232.7	6.7	231.1	2.6	7.0	0.6
2.68	258.2	6.6	253.6	7.1	256.5	6.8	256.1	2.3	6.8	0.3
2.29	281.1	6.5	276.0	7.2	280.7	6.7	279.3	2.8	6.8	0.4
1.81	300.2	7.2	298.1	7.3	299.4	7.3	299.2	1.1	7.3	0.1
1.67	306.0	7.3	n.d.	n.d.	n.d.	n.d.	306.0	n.d.	7.3	n.d.
1.42	325.1	6.7	319.5	7.2	326.7	6.8	323.8	3.8	6.9	0.3
0.96	342.8	7.4	344.1	7.3	347.9	6.8	344.9	2.7	7.2	0.3
0.74	359.6	6.7	355.0	7.7	358.8	6.7	357.8	2.5	7.0	0.6
0.48	364.4	7.1	367.3	7.5	366.6	7.0	366.1	1.5	7.2	0.3
0.25	373.6	7.1	375.8	7.1	371.9	6.9	373.8	2.0	7.0	0.1
-0.18	388.2	6.9	391.0	7.5	391.2	6.9	390.1	1.7	7.1	0.3
-0.48	402.7	6.8	399.1	7.0	396.6	7.2	399.5	3.1	7.0	0.2
-0.90	410.6	7.5	412.0	6.9	412.6	6.7	411.7	1.0	7.0	0.4
-1.41	435.2	6.5	425.4	6.8	433.0	7.6	431.2	5.1	7.0	0.6
-2.17	445.7	7.3	442.6	6.8	450.8	6.7	446.4	4.1	6.9	0.3
-2.85	461.8	7.4	456.6	7.0	463.6	6.7	460.7	3.6	7.0	0.4
-3.77	480.5	6.7	471.6	6.8	477.6	6.9	476.6	4.5	6.8	0.1
-4.61	490.6	6.7	483.5	6.8	488.0	7.0	487.4	3.6	6.8	0.2

Table S20 *EMF (mV) and temperature (T, °C) measurements of Metrohm Primatrode H electrode at 15 °C.*

pH of H_2SO_4 solution standard	Exp 1 EMF 1	T 1	Exp 2 EMF 2	T 2	Exp 3 EMF 3	T 3	EMF(mV) Average	±SD	T (°C) Average	±SD
4.00	179.7	15.9	n.d.	n.d.	n.d.	n.d.	179.7	n.d.	15.9	n.d.
3.52	203.5	16.5	205.0	16.0	204.2	15.7	204.2	0.8	16.1	0.4
3.12	228.7	16.3	228.9	15.9	228.8	16.2	228.8	0.1	16.1	0.2
3.02	235.7	16.4	n.d.	n.d.	n.d.	n.d.	229.5	n.d.	16.4	n.d.
2.68	254.2	16.4	254.4	15.9	254.3	15.9	254.3	0.1	16.1	0.3
2.30	277.6	16.5	280.6	15.7	277.9	15.8	278.7	1.7	16.0	0.4
1.82	302.8	15.9	306.0	15.8	304.4	16.1	304.4	1.6	15.9	0.2
1.67	315.2	15.7	n.d.	n.d.	n.d.	n.d.	306.0	n.d.	15.7	n.d.
1.44	325.4	15.8	326.6	16.1	327.7	16.2	326.6	1.2	16.0	0.2
0.97	351.7	16.1	351.4	15.9	354.0	15.7	352.4	1.4	15.9	0.2
0.76	362.9	15.8	362.7	16.0	364.3	16.2	363.3	0.9	16.0	0.2
0.50	374.1	15.9	375.4	16.2	375.3	16.0	374.9	0.7	16.0	0.2
0.27	384.6	15.9	386.1	16.0	386.3	16.0	385.7	0.9	16.0	0.1
-0.15	402.3	15.8	403.2	15.8	405.4	16.1	403.6	1.6	15.9	0.2
-0.45	411.7	15.7	413.4	15.8	413.4	16.0	412.8	1.0	15.8	0.2
-0.87	426.1	16.1	426.1	15.8	426.1	15.7	426.1	0.0	15.9	0.2
-1.37	438.4	15.7	447.2	15.9	442.6	16.0	442.7	4.4	15.9	0.2
-2.14	454	15.7	455.3	15.7	455.4	15.8	454.9	0.8	15.7	0.1
-2.83	467.4	15.7	469.1	15.7	467.4	15.7	468.0	1.0	15.7	0.0
-3.78	483	15.6	481.0	15.7	480.0	15.7	481.3	1.5	15.7	0.1
-4.65	493.1	16.1	489.1	15.7	483.3	15.7	488.5	4.9	15.8	0.2

Table S21 *EMF (mV) and temperature (T, °C) measurements of Metrohm Primatrode H electrode at 40 °C.*

pH of H_2SO_4 solution standard	Exp 1 EMF 1	T 1	Exp 2 EMF 2	T 2	Exp 3 EMF 3	T 3	EMF(mV) Average	±SD	T (°C) Average	±SD
4.04	185.5	38.8	n.d.	n.d.	n.d.	n.d.	185.5	n.d.	38.8	n.d.
3.52	216.3	38.0	226.3	38.2	216.2	38.0	219.6	5.8	38.1	0.1
3.12	249.4	38.7	249.9	38.6	242.3	38.2	247.2	4.3	38.5	0.3
2.99	251.6	37.8	n.d.	n.d.	n.d.	n.d.	229.5	n.d.	37.8	n.d.
2.68	274.2	38.6	270.3	38.5	270.4	38.3	271.6	2.2	38.5	0.2
2.30	294.5	38.4	294.5	38.5	294.7	38.4	294.6	0.1	38.4	0.1
1.82	322.3	38.4	322.2	38.4	321.2	38.0	321.9	0.6	38.3	0.2
1.69	331.9	38	n.d.	n.d.	n.d.	n.d.	306.0	n.d.	38.0	n.d.
1.44	346.4	38.4	346.6	38.5	347.2	38.3	346.7	0.4	38.4	0.1
0.97	373.4	38.3	373.9	38.4	374.3	38.3	373.9	0.5	38.3	0.1
0.76	384.9	38.3	385.7	38.6	386.2	38.5	385.6	0.7	38.5	0.2
0.50	399.6	38.4	399.6	38.4	400.4	38.5	399.9	0.5	38.4	0.1
0.27	411.5	38.6	411.8	38.5	412.1	38.7	411.8	0.3	38.6	0.1
-0.09	432	38.5	432.1	38.6	432.2	38.9	432.1	0.1	38.7	0.2
-0.39	444.4	38.7	443.9	38.7	444.2	38.8	444.2	0.3	38.7	0.1
-0.69	453.2	38.0	452.5	38.7	454.3	38.7	453.3	0.9	38.5	0.4
-1.27	469.4	38.8	468.8	38.7	471.1	38.8	469.8	1.2	38.8	0.1
-1.94	484.3	38.8	483.9	38.8	486.8	39.0	485.0	1.6	38.9	0.1
-2.67	497.6	38.7	498.9	38.8	500.9	38.9	499.1	1.7	38.8	0.1
-3.55	511.8	38.7	512.4	38.6	513.5	38.9	512.6	0.9	38.7	0.2
-4.80	526.1	38.6	526.3	38.6	527.7	38.9	526.7	0.9	38.7	0.2

Table S22 *EMF (mV) and temperature (T, °C) measurements of Metrohm Primatrode H electrode at 50 °C.*

pH of H_2SO_4 solution standard	Exp 1 EMF 1	T 1	Exp 2 EMF 2	T 2	Exp 3 EMF 3	T 3	EMF(mV) Average	±SD	T (°C) Average	±SD
4.01	186.3	47.9	n.d.	n.d.	n.d.	n.d.	186.3	n.d.	47.9	n.d.
3.52	220.5	47.7	215.8	47.6	215.8	47.2	217.4	2.7	47.5	0.3
3.14	247.9	47.7	239.0	47.5	241.9	47.6	242.9	4.5	47.6	0.1
2.99	254	48.0	n.d.	n.d.	n.d.	n.d.	229.5	n.d.	48.0	n.d.
2.71	273.1	47.5	271.3	47.6	268.0	47.3	270.8	2.6	47.5	0.2
2.34	298.4	47.5	296.7	47.2	295.4	47.5	296.8	1.5	47.4	0.2
1.86	329.1	47.5	325.0	47.1	326.8	47.5	327.0	2.1	47.4	0.2
1.71	338	48.0	n.d.	n.d.	n.d.	n.d.	306.0	n.d.	48.0	n.d.
1.49	352.5	47.4	349.3	47.2	348.2	47.2	350.0	2.2	47.3	0.1
1.05	379.6	47.4	377.6	47.1	376.7	47.2	378.0	1.5	47.2	0.2
0.80	393.9	47.4	391.6	47.3	389.1	47.4	391.5	2.4	47.4	0.1
0.54	409.3	47.7	408.1	46.6	403.7	47.3	407.0	2.9	47.2	0.6
0.31	420.9	48.1	420.6	47.3	418.8	47.3	420.1	1.1	47.6	0.5
-0.10	443.4	47.6	438.5	47.3	439.0	47.5	440.3	2.7	47.5	0.2
-0.38	454	47.9	451.5	47.4	450.2	47.6	451.9	1.9	47.6	0.3
-0.79	469.5	47.6	463.8	47.1	464.1	47.4	465.8	3.2	47.4	0.3
-1.27	484.3	47.9	477.9	47.3	477.9	47.8	480.0	3.7	47.7	0.3
-2.04	501	47.8	498.7	47.3	494.6	47.6	498.1	3.2	47.6	0.3
-2.76	515.7	47.8	506.9	47.4	510.2	47.8	510.9	4.4	47.7	0.2
-3.79	534	47.8	519.2	47.3	528.2	47.8	527.1	7.5	47.6	0.3
-4.76	548.5	48.1	531.4	47.4	540.4	47.5	540.1	8.6	47.7	0.4

Table S23 *EMF (mV) and temperature (T, °C) measurements of Metrohm Primatrode I electrode at 65 °C. Experiments were feasible only down to pH -0.69 as pH the electrode lost sensitivity at the pH -1.27 H_2SO_4 standard. The Metrohm Primatrode H was originally intended for these experiments, but it lost sensitivity during a negative-pH Fe(II) oxidation run at 65 °C on 6 June 2025. Consequently, it could not be used and the Primatrode I was used to conduct the negative pH calibration at 65 °C instead.*

pH standard (Hanna buffers & H_2SO_4 solutions)	EMF (mV)	T (°C)	CF (mV)
9.77	-173.3	59.6	-1.3
6.98	4.0	59.0	2.6
4.09	195.7	58.4	-1.3
3.52	224.2	58.4	6.9
3.12	252.9	58.5	4.0
3.00	270.0	60.4	-5.0
2.68	283.8	60.8	1.9
1.82	341.1	60.9	0.2
1.44	367.1	61.8	-0.6
0.97	395.8	61.3	0.7
0.76	410.3	61.6	0.1
0.50	425.1	61.3	2.4
0.27	438.5	61.1	3.8
-0.09	459.4	61.6	5.9
-0.39	473.0	62.0	12.2
-0.69	495.3	62.0	9.1

S8 pH glass electrode performance monitoring

The pH probes were calibrated at the start of each day prior to conducting experiments with very low and negative pH solutions. The percent slope of the pH calibration curve was monitored to be within the acceptable range of 95-103%. The zero point, pH(0), the pH at which the probe output voltage is zero, was also monitored over time. These values remained stable at approximately pH 6.94 ± 0.03 for the Unitrode electrode and pH 7.12 ± 0.02 for the Primatrode electrode. The % slope was 98.9 ± 0.9 for the Unitrode and 98.7 ± 0.6 for the Primatrode. Both the % slope and pH(0) showed no significant drift or signs of contamination from the reference electrolyte. Freshly opened Hanna pH buffer solutions were used starting on 12 March 2025.

Table S24 *Standard 3-point pH calibrations of Primatrode H electrode collected between October 2024 and May 2025.*

Date	pH 4.01 buffer			pH 7.01 buffer			pH 10.01 buffer		
	pH	EMF (mV)	T (°C)	pH	EMF (mV)	T (°C)	pH	EMF (mV)	T (°C)
16/10/2024	4.009	181.5	24.6	7.012	7.1	24.6	10.014	-165.8	24.6
04/11/2024	4.009	180.8	24.4	7.012	6.4	24.5	10.015	-166.3	24.5
08/11/2024	4.009	182.2	24.5	7.012	7.0	24.5	10.015	-166.7	24.5
11/11/2024	4.009	181.9	24.6	7.012	6.6	24.6	10.015	-167.4	24.5
15/11/2024	4.009	181.7	24.4	7.012	6.9	24.5	10.015	-166.8	24.5
20/11/2024	4.009	181.4	24.6	7.012	6.4	24.6	10.015	-167.2	24.5
27/11/2024	4.009	181.8	24.5	7.012	6.1	24.4	10.015	-168.2	24.5
17/12/2024	4.009	181.7	24.3	7.012	5.9	24.4	10.015	-167.2	24.5
07/02/2025	4.009	181.0	24.5	7.012	6.0	24.5	10.014	-167.1	24.5
10/02/2025	4.009	183.0	24.7	7.011	7.6	24.6	10.014	-165.8	24.6
17/02/2025	4.009	182.2	24.7	7.011	7.3	24.7	10.013	-165.9	24.7
18/02/2025	4.009	182.2	24.3	7.013	6.9	24.3	10.016	-166.4	24.4
19/02/2025	4.008	182.3	24.1	7.013	7.0	24.2	10.018	-166.2	24.2
20/02/2025	4.009	181.3	24.4	7.012	6.0	24.5	10.014	-167	24.6
21/02/2025	4.009	181.4	24.7	7.011	6.3	24.7	10.013	-167	24.7
24/02/2025	4.009	181.1	24.6	7.011	5.8	24.7	10.013	-167.4	24.7
25/02/2025	4.009	181.5	24.7	7.011	6.0	24.7	10.013	-167	24.7
12/03/2025	4.004	180.8	21.9	7.021	5.9	22.1	10.038	-171.1	22.2
17/03/2025	4.009	180.4	24.3	7.013	4.5	24.3	10.017	-171.7	24.3
18/03/2025	4.009	180.7	24.4	7.012	4.6	24.4	10.016	-172.1	24.4
19/03/2025	4.009	180.2	24.3	7.012	4.1	24.4	10.016	-172.2	24.4
07/04/2025	4.009	179.9	24.4	7.012	4.1	24.5	10.015	-172.3	24.5
08/04/2025	4.009	179.9	24.5	7.012	4.0	24.5	10.015	-172.2	24.5
09/04/2025	4.009	181.5	24.4	7.012	5.4	24.4	10.016	-170.6	24.4
10/04/2025	4.009	181.6	24.4	7.012	5.6	24.5	10.015	-170.4	24.5
01/05/2025	4.009	181.1	24.4	7.012	5.1	24.5	10.014	-168.5	24.6

Table S25 *Standard 3-point pH calibrations of Orion Ross Ultra pH/ATC Triode electrode collected between November 2022 and March 2025 from the Kucernak Group (dataset provided by Yuchen Sun).*

Date	pH 10 buffer			pH 7 buffer			pH 4 buffer		
	pH	EMF (mV)	T (°C)	pH	EMF (mV)	T (°C)	pH	EMF (mV)	T (°C)
29/11/2022	10.045	-169.1	21.8	7.008	1.0	21.6	4.006	175.7	21.6
05/01/2023	10.049	-172.0	21.5	7.008	1.2	21.4	4.006	175.7	21.5
06/02/2023	10.049	-170.2	21.4	7.008	-0.2	21.4	4.006	170.4	21.5
06/03/2023	10.044	-160.3	21.9	7.007	-2.9	21.9	4.006	173.3	22.0
26/05/2023	10.055	-174.9	20.8	7.011	-6.4	20.8	4.005	168.9	20.9
23/06/2023	10.013	-177.1	25.0	6.997	-8.4	25.0	4.010	168.0	25.2
11/08/2023	10.067	-177.7	19.8	7.014	-7.5	19.7	4.040	167.4	19.5
25/08/2023	10.066	-179.5	19.9	7.013	-9.3	20.0	4.005	167.0	20.1
29/09/2023	10.046	-179.4	21.7	7.007	-11.9	21.7	4.006	162.7	21.8
26/10/2023	10.038	-179.7	22.5	7.005	-11.8	22.5	4.007	163.1	22.5
30/11/2023	10.051	-178.9	21.2	7.008	-12.9	21.4	4.006	165.3	21.5
25/01/2024	10.035	-181.2	22.8	7.004	-12.1	22.8	4.007	159.8	22.6
29/02/2024	10.038	-182.8	22.5	7.004	-16.2	22.6	4.007	159.3	22.7
25/03/2024	10.049	-184.7	21.5	7.008	-15.6	21.5	4.006	156.6	21.6
10/05/2024	10.056	-186.4	20.8	7.010	-17.8	20.9	4.005	156.2	20.8
30/05/2024	10.049	-185.4	21.4	7.009	-17.4	21.1	4.006	155.6	21.0
27/06/2024	10.065	-178.1	19.9	7.013	-17.4	20.0	4.005	154.4	20.1
01/08/2024	10.050	-178.5	21.3	7.010	-19.1	21.0	4.006	153.7	21.0
29/08/2024	10.005	-180.0	21.5	7.008	-18.2	21.4	4.006	154.5	21.4
26/09/2024	10.056	-180.2	20.8	7.010	-19.1	20.8	4.005	151.2	20.7
31/10/2024	10.053	-181.5	21.1	7.011	-20.7	20.7	4.005	150.2	20.9
27/11/2024	10.058	-182.8	20.6	7.012	-21.6	20.5	4.005	146.7	20.6
31/01/2025	10.061	-184.2	20.3	7.012	-25.3	20.2	4.005	144.5	20.3
27/02/2025	10.045	-182.9	21.8	7.007	-22.5	21.9	4.006	147.7	21.6
28/03/2025	10.045	-182.9	21.8	7.007	-22.5	21.9	4.006	147.7	21.6

Table S26 *Standard 3-point pH calibrations of Orion Ross Sure-Flow electrode collected between June 2023 and April 2025 from the Kucernak Group (dataset provided by Yuchen Sun).*

Date	pH 7 buffer			pH 4 buffer			pH 1.68 buffer		
	pH	EMF (mV)	T (°C)	pH	EMF (mV)	T (°C)	pH	EMF (mV)	T (°C)
26/06/2023	6.997	-5.2	25.0	4.010	167.4	25.0	1.678	304.0	25.0
11/08/2023	7.015	-6.8	19.5	4.004	167.3	19.5	1.674	299.9	19.5
13/09/2023	7.015	-10.4	19.5	4.004	163.0	19.5	1.674	296.3	19.5
09/10/2023	7.008	-12.8	21.4	4.006	164.0	21.4	1.676	293.7	21.4
13/11/2023	7.006	-18.8	22.3	4.004	157.0	19.4	1.674	290.9	19.8
29/11/2023	7.003	-18.4	22.9	4.007	157.2	22.3	1.676	290.6	22.4
03/01/2024	7.006	-21.2	22.1	4.007	152.5	22.1	1.676	283.4	22.1
08/01/2024	7.006	-24.2	22.7	4.007	152.4	22.7	1.677	285.0	22.7
26/02/2024	7.006	-25.2	21.2	4.006	149.6	22.0	1.675	277.9	21.2
11/04/2024	7.006	-26.5	22.4	4.005	147.7	22.4	1.675	275.3	22.4
29/04/2024	7.009	-37.1	21.2	4.006	140.0	21.4	1.675	265.4	21.2
07/05/2024	7.012	-32.7	20.4	4.005	140.4	20.3	1.675	265.4	20.2
19/06/2024	7.011	-36.8	20.8	4.005	136.6	20.7	1.675	258.9	20.7
23/07/2024	7.009	-39.5	21.2	4.006	134.4	21.2	1.675	260.3	21.1
29/08/2024	7.008	-40.9	21.5	4.006	129.9	21.5	1.867	250.3	21.5
27/09/2024	7.015	-45.0	19.5	4.004	127.6	19.5	1.935	244.8	19.5
01/11/2024	7.013	-45.2	20.0	4.005	124.8	20.1	2.034	240.0	20.1
06/12/2024	7.014	-48.3	19.8	4.005	120.3	19.9	1.675	260.5	19.9
14/01/2025	7.012	-34.7	20.3	4.005	118.5	20.3	1.778	254.7	20.3
26/02/2025	7.012	-54.8	20.5	4.008	114.4	20.5	1.868	249.4	20.5
28/03/2025	7.008	-53.0	21.5	4.416	111.6	21.5	1.871	250.3	21.5
30/04/2025	7.132	-60.2	21.4	4.212	107.9	21.5	1.966	24.5	21.5

***Table S27** Summary of calibration data for Metrohm Primatrode H electrode collected between October 2024 and May 2025, including temperature at calibration, standard electrode potential (E_0), electrode slope (S), percentage of the theoretical Nernstian slope (%Slope), Isopotential point or Zero point (pH(0)), and the offset potential (dU).*

Date	Temp (°C)	E_0 (mV)	S (mV·pH^{-1})	%Slope	pH(0)	dU (mV)
16/10/2024	24.6	57.84	413.1	97.9	7.14	8.3
04/11/2024	24.5	57.79	412.2	97.8	7.13	7.7
07/11/2024	24.6	58.08	415.0	98.3	7.14	8.4
08/11/2024	24.5	58.09	414.8	98.3	7.14	8.2
11/11/2024	24.6	58.16	414.8	98.4	7.13	7.7
15/11/2024	24.5	58.03	414.1	98.2	7.14	8.0
20/11/2024	24.6	58.04	413.9	98.2	7.13	7.6
27/11/2024	24.5	58.28	415.2	98.7	7.12	7.3
17/12/2024	24.4	58.09	414.1	98.4	7.13	7.5
07/02/2025	24.5	57.97	413.1	98.1	7.13	7.3
10/02/2025	24.6	58.08	415.5	98.3	7.15	8.9
17/02/2025	24.7	57.98	414.4	98.1	7.15	8.5
18/02/2025	24.3	58.03	414.5	98.3	7.14	8.3
19/02/2025	24.2	57.99	414.4	98.3	7.15	8.5
20/02/2025	24.5	58.00	413.5	98.2	7.13	7.4
21/02/2025	24.7	58.03	413.7	98.2	7.13	7.5
24/02/2025	24.7	58.04	413.5	98.2	7.12	7.1
25/02/2025	24.7	58.04	413.8	98.2	7.13	7.5
12/03/2025	22.1	58.32	414.7	99.5	7.11	6.4
17/03/2025	24.3	58.61	415.4	99.3	7.09	5.2
18/03/2025	24.4	58.73	416.2	99.5	7.09	5.1
19/03/2025	24.4	58.66	415.4	99.4	7.08	4.8
07/04/2025	24.5	58.64	415.1	99.3	7.08	4.6
08/04/2025	24.5	58.62	415.0	99.2	7.08	4.6
09/04/2025	24.4	58.61	416.5	99.3	7.11	6.2
10/04/2025	24.5	58.61	416.6	99.2	7.11	6.3
01/05/2025	24.5	58.22	414.1	98.6	7.11	6.6

***Table S28** Summary of calibration data for Metrohm Unitrode N electrode collected between March 2024 and May 2025, including temperature at calibration, standard electrode potential (E_0), electrode slope (S), percentage of the theoretical Nernstian slope (%Slope), Isopotential point or Zero point (pH(0)), and the offset potential (dU).*

Date	Temp (°C)	E_0 (mV)	S (mV·pH^{-1})	%Slope	pH(0)	dU (mV)
17/03/2025	21.5			97.1	7.00	
11/04/2025	20.8			99.5	6.92	
04/04/2025	22.2			99.2	6.94	
07/04/2025	24.7			98.4	6.94	
08/04/2025	24.5			99.6	6.92	
09/04/2025	24.6			99.8	6.92	
01/05/2025	24.5			98.6	6.96	

Table S29 *Summary of calibration data for Orion Ross Ultra pH/ATC Triode electrode collected between November 2022 and March 2025, including temperature at calibration, standard electrode potential (E_0), electrode slope (S), percentage of the theoretical Nernstian slope (%Slope), Isopotential point or Zero point (pH(0)), and the offset potential (dU).*

Date	Temp (°C)	E_0 (mV)	S (mV·pH^{-1})	%Slope	pH(0)	dU (mV)
29/11/2022	21.7	403.3	57.09	97.6	7.06	3.7
05/01/2023	21.5	405.6	57.54	98.4	7.05	2.8
06/02/2023	21.4	395.7	56.36	96.4	7.02	1.2
06/03/2023	21.9	391.1	55.24	94.3	7.08	4.4
26/05/2023	20.8	395.0	56.82	97.4	6.95	-2.8
23/06/2023	25.1	396.9	57.49	97.1	6.91	-5.5
11/08/2023	19.7	397.1	57.25	98.5	6.94	-3.6
25/08/2023	20.0	394.5	57.17	98.3	6.90	-5.7
29/09/2023	21.7	388.0	56.64	96.8	6.85	-8.4
26/10/2023	22.5	389.3	56.84	96.9	6.85	-8.5
30/11/2023	21.4	390.9	56.93	97.4	6.87	-7.6
25/01/2024	22.7	385.7	56.57	96.3	6.82	-10.3
29/02/2024	22.6	384.7	56.72	96.6	6.78	-12.3
25/03/2024	21.5	382.0	56.48	96.6	6.76	-13.4
10/05/2024	20.8	381.7	56.62	97.0	6.74	-14.7
30/05/2024	21.2	380.5	56.43	96.6	6.74	-14.5
27/06/2024	20.0	371.9	54.86	94.3	6.78	-12.2
01/08/2024	21.1	371.3	54.96	94.1	6.76	-13.4
29/08/2024	21.4	376.1	55.76	95.4	6.75	-14.2
26/09/2024	20.8	368.6	54.76	93.9	6.73	-14.7
31/10/2024	20.9	367.8	54.84	94.0	6.71	-16.1
27/11/2024	20.6	363.2	54.43	93.4	6.67	-17.9
31/01/2025	20.3	359.6	54.27	93.2	6.63	-20.3
27/02/2025	21.8	365.0	54.74	93.5	6.67	-18.2
28/03/2025	21.8	365.0	54.74	93.5	6.67	-18.2

Table S30 *Summary of calibration data for Orion Ross Sure-Flow electrode collected between June 2023 and April 2025, including temperature at calibration, standard electrode potential (E_0), electrode slope (S), percentage of the theoretical Nernstian slope (%Slope), Isopotential point or Zero point (pH(0)), and the offset potential (dU).*

Date	Temp (°C)	E_0 (mV)	S (mV·pH^{-1})	%Slope	pH(0)	dU (mV)
26/06/2023	25.0	58.12	401.1	98.2	6.90	-5.7
11/08/2023	19.5	57.44	396.5	98.9	6.90	-5.6
13/09/2023	19.5	57.43	392.6	98.9	6.84	-9.4
09/10/2023	21.4	57.55	391.7	98.5	6.81	-11.1
13/11/2023	20.5	58.11	388.7	99.7	6.69	-18.0
29/11/2023	22.5	58.03	388.5	98.9	6.70	-17.7
03/01/2024	22.1	57.18	380.1	97.6	6.65	-20.2
08/01/2024	22.7	58.06	383.3	98.9	6.60	-23.1
26/02/2024	21.5	56.92	374.8	97.4	6.58	-23.6
11/04/2024	22.4	56.68	371.8	96.6	6.56	-24.9
29/04/2024	21.3	56.82	363.1	97.2	6.39	-34.6
07/05/2024	20.3	55.94	361.0	96.1	6.45	-30.5
19/06/2024	20.7	55.52	354.4	95.2	6.38	-34.2
23/07/2024	21.2	56.29	356.5	96.4	6.33	-37.5
29/08/2024	21.5	56.66	356.4	96.9	6.29	-40.2
27/09/2024	19.5	57.07	355.5	98.3	6.23	-43.9
01/11/2024	20.1	57.22	355.5	98.3	6.21	-45.1
06/12/2024	19.9	57.75	355.2	99.3	6.15	-49.1
14/01/2025	20.3	55.05	347.6	94.5	6.31	-37.7
26/02/2025	20.5	58.95	356.3	101.2	6.04	-56.4
28/03/2025	21.5	59.06	364.7	101.0	6.18	-48.7
30/04/2025	21.5	59.00	359.4	100.9	6.09	-53.7

S9 Negative pH Calibration Curve fitting Using a Logistic Function

A non-linear calibration approach is required to account for the acid error exhibited by glass pH electrodes under highly acidic conditions. Below approximately pH 2 and above pH 12, the electrode response begins to deviate from the expected Nernstian response, leading to a flattening of the curve. This behaviour is not well captured by linear or polynomial models.

In this study, a logistic function was chosen to model the relationship between pH and the measured electrode potential (in millivolts) because it can represent the sigmoidal, non-linear shape of the electrode response under extreme pH conditions. The following model is the standard 4-parameter logistic equation. Where: L is the lower asymptote, A is the amplitude, k is the steepness of the curve, and x_0 is the inflection point where the potential changes most rapidly with pH.

$$EMF(mV) = L + \frac{A}{1+e^{-k\times(pH - x_0)}} \qquad \text{Eq. S2}$$

To simplify the model, the lower asymptote (L) was set to zero and the logistic function reduces to only 3 parameters. Since the calibration only covers part of the full sigmoid curve (pH −5 to 4), fixing L = 0 allows A to directly represent the upper asymptote. This reduces model complexity while still providing a good fit, as illustrated in Fig. S11, where the red box highlights the portion of the curve being modelled. X_0

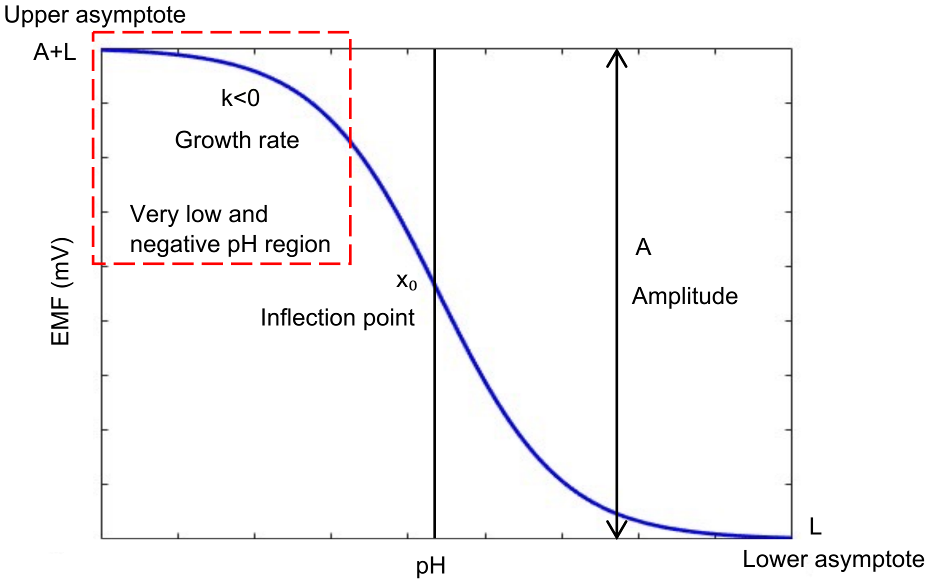

Figure S11 *Anatomy of a logistic function*

S10 Logistic fit uncertainties analysis using Monte Carlo simulation

$EMF(x) = A/(1+exp(-k*(x-x0)))$

$EMF(x) = (A±A_{SD})/(1+exp((-k±k_{SD})*(x-(x0±x0_{SD}))))$

Table S31 *Logistic fit uncertainties analysis using Monte Carlo simulation*

Coefficients (with 95% confidence bounds)	Logistic fit (n=1)	Logistic fit + Monte Carlo simulation (n = 5000)
A	518.0 (513.1, 522.7)	518.0 (513.8, 522.1)
k	-0.4659 (-0.4826, -0.4493)	-0.4660 (-0.4770, -0.4557)
X0	2.734 (2.678, 2.790)	2.734 (2.697, 2.770)

S11 Effect of Temperature on Electrode Response

The electrode response is influenced by temperature in both the normal and negative pH ranges. The standard calibration curves (Fig. S12) obtained between 6.6 °C and 57.8 °C show that the sensitivity with temperature is greater at temperature increases, in agreement with the Nernst equation. Linear regression of the slope S against temperature (Fig. S12a) gave a temperature coefficient of the Nernstian slope (k) of = (1.99 ± 0.10)·10^{-1} mV·C^{-1}·pH^{-1} with an offset (C) of -0.54 ± 3.21 mV·pH^{-1}. Similarly, linear regression of the standard potential (E_0) against temperature (Fig. S12b) indicated a value of E_0 at 25 °C of 418.9 ± 1.4 mV, with an experimentally determined temperature dependence of standard potential, $\frac{dE_0}{dT}$ = 1.13 ± 0.08 mV·C^{-1}. This measured $\frac{dE_0}{dT}$ value is slightly higher than the theoretical value of 1.00 mV·C^{-1}, which would correspond to ideal Nernstian behaviour.

The negative pH calibration curves display a non-linear response, with electrode potentials increasing systematically as temperature rises. Logistic functions fitted to the EMF-pH data at each temperature reveal that, at higher temperatures, the curves plateau at progressively higher EMF values. This plateau corresponds to the amplitude parameter (A), which was best described by a 3rd degree polynomial as a function of temperature (Fig. S13a). Below 20 °C, amplitude values remain relatively stable at around 500 mV; between 20 - 40 °C, the amplitude rises more steeply, and above 40 °C the increase slows, reaching ~550 mV at 48 °C. The growth rate parameter (k) is also expressed by a third-degree polynomial relationship with temperature (Fig. S13b). (k) increases from around 0.43 pH^{-1} at 5 °C to 0.49 pH^{-1} at 20 °C, then declines slightly between 20 to 40 °C. Beyond 40 °C, (k) rises again, with a maximum value of 0.51 pH^{-1} observed at 48 °C. In contrast, the midpoint parameter (x_0) displayed a bell-shaped dependence on temperature, best captured by a 2nd degree polynomial (Fig. S13c). (x_0)increases from pH = 2.70 at 5 °C to a maximum of pH = 2.78 around 25 °C, before gradually decreasing to pH = 2.64 by 48 °C. The polynomial degrees for each parameter were selected solely based on the basis of the AICc analysis presented in Fig. S14 (Lever et al., 2016), and the fitted coefficients are summarised in Table 2.1. For negative pH calibrations, curves are shown only between the minimum and maximum experimental temperatures to avoid extrapolation of the logistic parameters-temperature relationships.

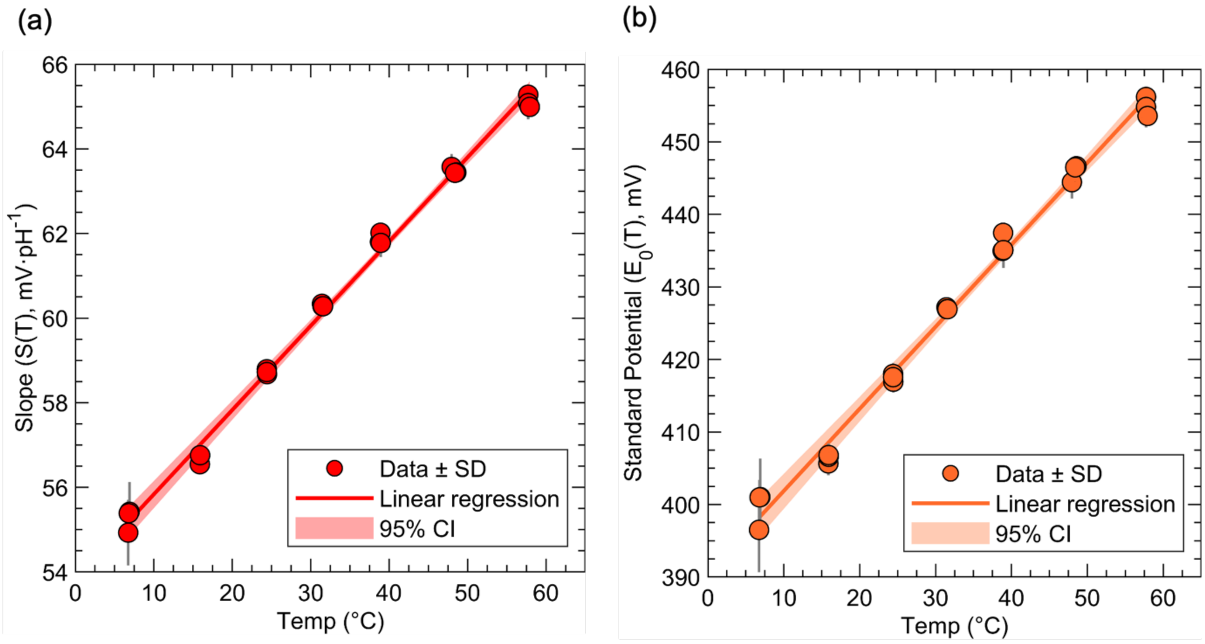

Figure S12 *Temperature dependence of Nernst equation parameters (E_0 and S) derived from the standard pH calibrations at 5 to 60 °C. (a) Standard potential $E_0(T)$ ± SD (mV) plotted against temperature in °C, fitted by linear regression (orange line) with 95% confidence interval bands (orange shading). (b) Electrode slope S(T) ± SD (mV·pH^{-1}) plotted against temperature in °C, fitted by linear regression (red line) with 95% confidence interval bands (red shading).*

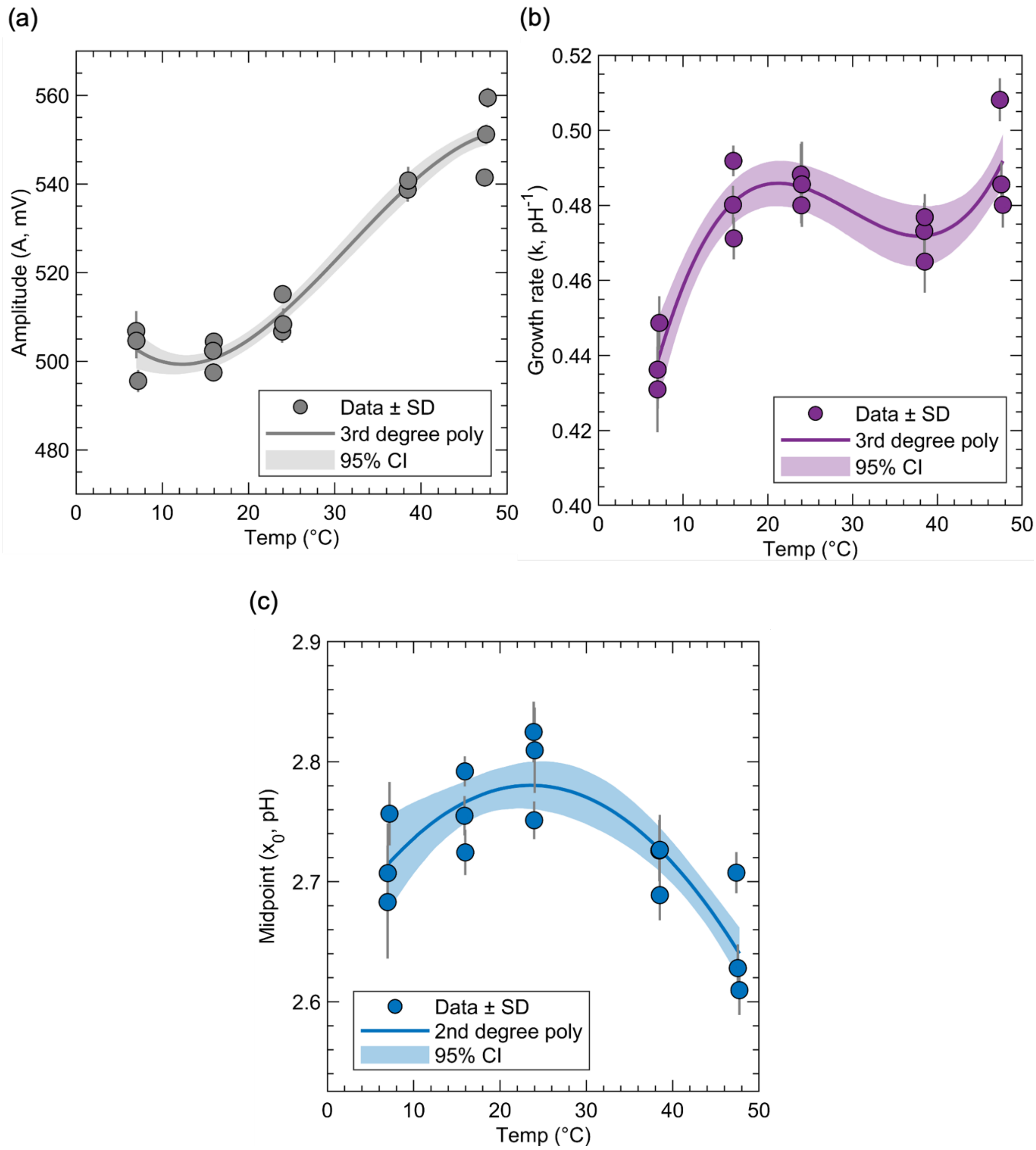

Figure S13 *Temperature dependence of Logistic equation parameters (A, k and x_0) derived from negative pH calibrations at 5 to 50 °C. The parameters of the logistic fit (a) Amplitude (A, mV), (b) Growth rate (k, pH^{-1}), and (c) Midpoint (x_0, pH) are plotted as a function of temperature. Each data point represents the best-fit parameter obtained from logistic fitting of EMF-pH data at the corresponding temperature. Polynomial functions (order selected based on AICc analysis) were applied to describe the temperature dependence, shown as solid lines with 95% confidence interval bands (shading).*

Table S32 *Results of temperature dependence of coefficients in the Nernst equation and logistic negative pH calibration.*

Equation		Coefficients	R^2
Standard pH Calibration, $EMF_{Nernst} = E_0(T) + S(T)\cdot pH$			
(2.9)	$S(T) = k\cdot(T + 273.15) + C$	$k= 1.99 \pm 0.10\cdot10^{-1}$, $C = -0.54 \pm 3.21$	1.00
(2.10)	$E_0(T)=E_{0\text{ at }25°C}+\frac{dE_0}{dT}\cdot(T - 25)$	$E_{0\text{ at }25°C} = 418.9 \pm 1.4$, $\frac{dE_0}{dT}=1.13 \pm 0.08$	0.99
Negative pH Calibration, $EMF_{Logistic} = \frac{A(T)}{1+e^{-k(T)(pH - x_0(T))}}$			
(2.11)	$A(T) = C_{3A}\cdot T3 +C_{2A}\cdot T^2 +C_{1A}\cdot T +C_{0A}$	$C_{3A} =-1.87 \pm0.75\cdot10^{-3}$, $C_{2A} =1.76 \pm 0.62\cdot10^{-1}$, $C_{1A} =-3.49 \pm 1.55$, $C_{0A} =519 \pm 12$	0.95
(2.12)	$k(T) = C_{3k}\cdot T3 +C_{2k}\cdot T^2 +C_{1k}\cdot T +C_{0k}$	$C_{3k} =5.99 \pm 2.01\cdot10^{-6}$, $C_{2k} =-5.32 \pm 1.67\cdot10^{-4}$, $C_{1k} =1.45 \pm 0.42\cdot10^{-2}$, $C_{0k} =3.61 \pm 0.32\cdot10^{-1}$	0.81
(2.13)	$x_0(T) = C_{2x0}\cdot T^2 +C_{1x0}\cdot T +C_{0x0}$	$C_{2x0} =-2.41 \pm 0.92\cdot10^{-4}$, $C_{1x0} =1.14 \pm 0.56\cdot10^{-2}$, $C_{0x0} =2.65 \pm 0.07$	0.66

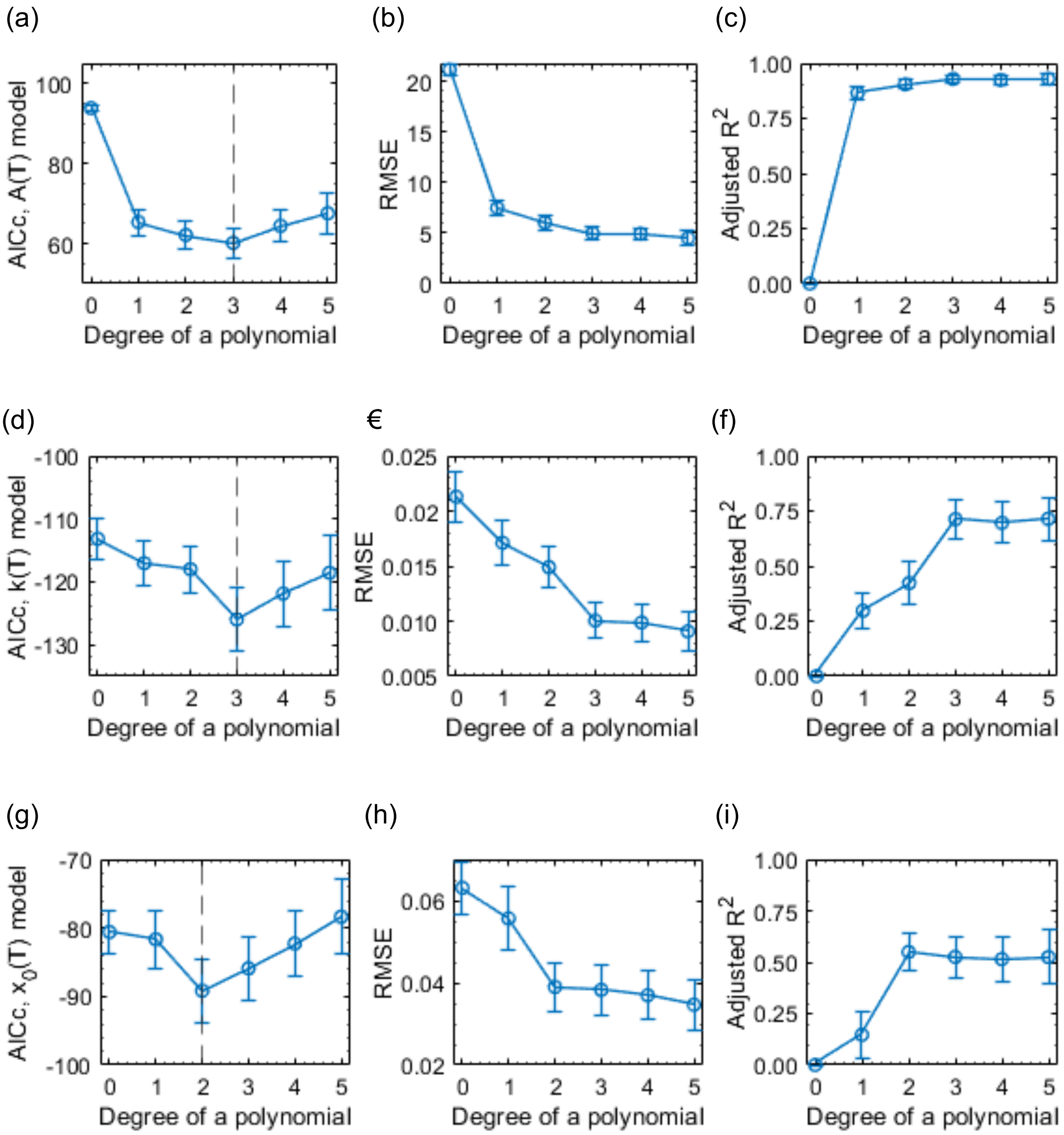

Figure S14 *Polynomial degree selection based on the Akaike Information Criterion corrected for small sample size (AICc), root mean square error (RMSE), and adjusted* R^2 *for modelling the temperature dependence of logistic coefficients.*

S12 Different non-linear calibration from different electrodes

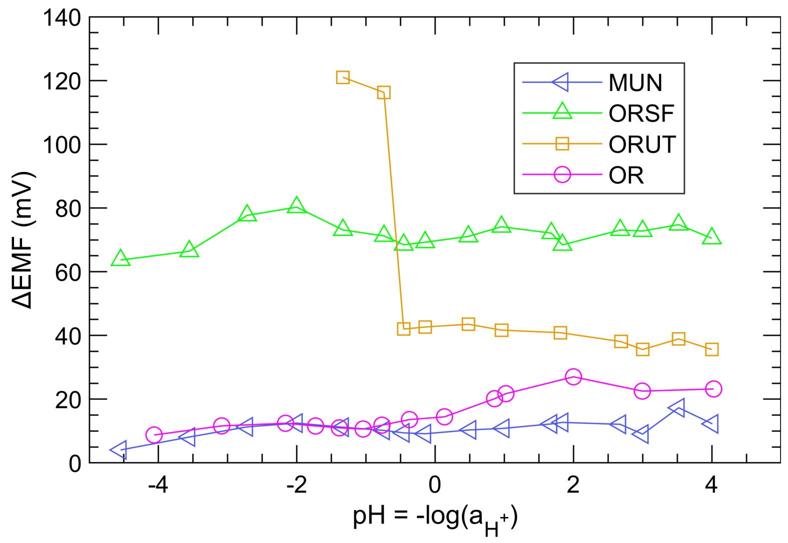

Figure S15 *Comparison of EMF measurements of each electrode relative to the Primatrode negative pH calibration curve; Unitrode (blue triangles), Orion Ross Sure-Flow (green triangles), and Orion Ross Ultra pH/ATC Triode (orange square), and Orion Ross (magenta circles).*

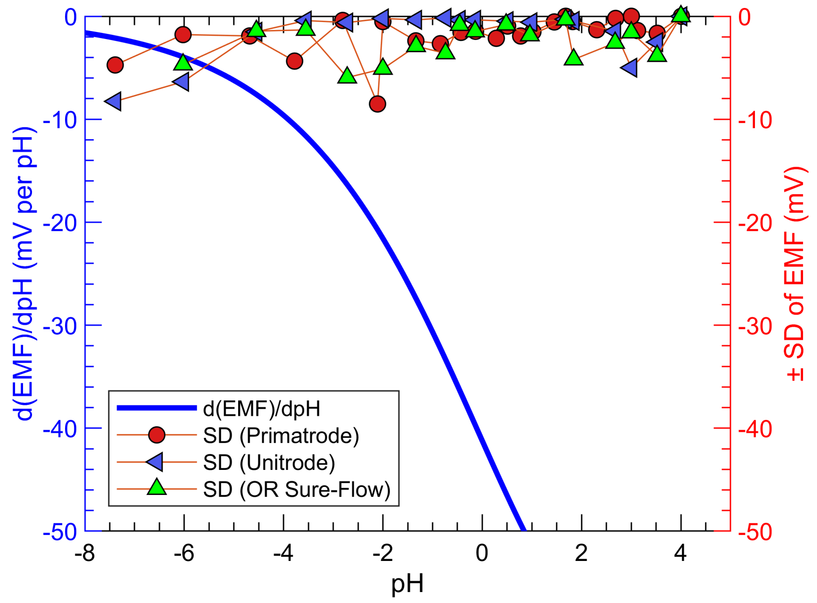

Figure S16 *Comparison of theoretical electrode sensitivity and experimental measurement uncertainty. The derivative of $EMF_{Non\text{-}linear}$ with respect to pH, shown as the solid blue line, represents the theoretical electrode sensitivity, while experimental measurement uncertainty is represented by the standard deviation (SD) of EMF measurements at each pH value (n=3) for the Primatrode (red circles), Unitrode (blue triangles), and Orion Ross Sure-Flow (green triangles) electrodes*
